# Co-occurrence of dissociation and anxiety and depressive disorders in adults: a scoping review

**DOI:** 10.3389/fpsyt.2026.1866005

**Published:** 2026-08-13

**Authors:** Anna Dunalska, Aleksandra Lewandowska, Kinga Lichtarska, Agata Szulc, Andrzej Jakubczyk, Natalia Szejko

**Affiliations:** 1 Psychiatric Clinic, Faculty of Health Sciences, Medical University of Warsaw, Warsaw, Poland; 2 Department of Child Psychiatry, Babiński Hospital, Łodz, Poland; 3 Department of Psychiatry, Medical University of Warsaw, Warsaw, Poland; 4 Clinic of Psychiatry, Social Psychiatry and Psychotherapy, Hannover Medical School, Hannover, Germany; 5 Department of Health Sociology, Education and Medical Communication, Institute of Mother and Child, Warsaw, Poland

**Keywords:** anxiety, depersonalization–derealization, depression, dissociation, functional neurological disorder, dissociative disorders, comorbidity, scoping review

## Abstract

**Background:**

Dissociation, including depersonalization, derealization, dissociative identity disorder or dissociation in the context of functional neurological disorder, is classically associated with trauma-related disorders but is increasingly reported in anxiety and depressive disorders. Despite growing evidence linking dissociation to affective symptom severity, functional impairment, and treatment outcomes, its role in anxiety and depression remains insufficiently synthesized.

**Objective:**

This scoping review aimed to map the extent, nature, and characteristics of dissociation in adults with anxiety and depressive symptoms, with a focus on conceptualization, assessment methods, prevalence, and clinical correlates.

**Methods:**

Using Joanna Briggs Institute methodology and PRISMA-ScR guidelines, PubMed and Scopus were searched from inception to January 2025. Studies assessing dissociation in adults with anxiety and/or depressive symptoms using validated instruments or clinical interviews were included. Data were charted and synthesized thematically.

**Results:**

A total of 300 studies were included. Of these, 49 studies focused on depersonalization–derealization phenomena, 11 studies examined dissociative identity disorder or complex dissociative disorders, 139 studies investigated functional neurological disorders, and 101 studies addressed mixed or transdiagnostic dissociative phenomena. Across anxiety and depressive disorders, dissociative symptoms were common and dimensionally distributed across clinical samples, with severity varying by diagnosis, setting, and assessment method. In depersonalization-focused studies, approximately 41-43% of patients met criteria for at least one anxiety disorder, and comorbid depression was reported in 62% to 84.8% of clinical series. In functional neurological disorder populations, dissociative symptoms- most commonly assessed with the DES- were elevated in approximately 30–60% of patients and were strongly associated with comorbid anxiety and depression. In FND sample, anxiety and depressive symptoms were highly prevalent, affecting approximately one-third to two-thirds of patients. Across diagnostic categories, higher dissociation was consistently associated with greater symptom severity, increased functional impairment, higher rates of suicidality, and poorer treatment outcomes.

**Conclusions:**

This scoping review demonstrates that dissociation is a frequent and clinically relevant feature of anxiety and depressive disorders, extending beyond traditional dissociative diagnoses and intersecting with functional neurological symptoms. The findings support transdiagnostic and dimensional models of psychopathology, in which dissociation reflects stress-related disturbances in self-experience and emotional processing. Routine assessment of dissociative symptoms in anxiety and depression may enhance clinical characterization and inform more targeted interventions. Future research should prioritize longitudinal and mechanistic studies to clarify causal pathways and treatment implications.

**Systematic Review Registration:**

https://osf.io/hnjpb/, identifier hnjpb.

## 1 Introduction

Dissociation, defined as a disruption in the normal integration of consciousness, memory, identity, emotion, perception, body representation, motor control, and behavior, is a complex and heterogeneous phenomenon observed across a spectrum of mental health conditions (1). While dissociative symptoms are most prominently featured in dissociative and trauma-related disorders, accumulating evidence suggests they also frequently co-occur in other psychiatric conditions, including depressive and anxiety disorders (2, 3). Despite this, the role of dissociation in these disorders remains underrecognized and under-investigated, and its clinical relevance is often overlooked in routine assessment and treatment planning (4).

Depression and anxiety are among the most prevalent mental health disorders worldwide, characterized by considerable symptom overlap, high comorbidity rates, and significant functional impairment (5–9). The presence of dissociative symptoms in individuals with depression and/or anxiety has been linked to more severe clinical presentations, poorer treatment outcomes, and diminished quality of life (10–12). Dissociation may be associated with poorer treatment response in depression and has been proposed as a potential marker of treatment resistance; it may also contribute to emotional dysregulation and avoidance behaviors commonly observed in anxiety disorders (13, 14).

Despite these important clinical implications, the literature on dissociation in depression and anxiety remains fragmented (15). Prior studies have tended to conceptualize dissociation either narrowly, focusing on severe dissociative disorders, or broadly, without differentiating among psychiatric diagnoses (16, 17). Furthermore, although dissociative symptoms are frequently assessed using instruments such as the Dissociative Experiences Scale (DES), the available studies on their prevalence, severity, and clinical relevance in depressive and anxiety disorders are heterogeneous, highlighting the need for systematic synthesis (2). It is also unclear whether particular types of dissociation- such as depersonalization-derealization disorder (DPDR), dissociative identity disorder (DID) and complex dissociative disorders (CDD) including dissociative identity disorder (DID) and partial dissociative identity disorder (pDID), or functional neurological disorder (FND), classified as a dissociative disorder in International Classification of Diseases, 11th Revision (ICD-11)- are more common among patients with anxiety and depression. As such, there is a pressing need to clarify the scope and nature of dissociation in these conditions, including its potential impact on symptomatology and treatment response (15).

This scoping review aims to comprehensively map the existing literature on dissociative phenomena in adults with depressive and anxiety symptoms. It focuses on how dissociation is conceptualized and measured in these populations, its reported prevalence and severity, and its potential impact on the clinical course and treatment outcomes of these conditions. We also map how the different types of dissociation co-occur with anxiety and depression and, where the literature permits, use this mapping to generate hypotheses about the possible directionality of these associations for future longitudinal research. By synthesizing the available evidence, this review seeks to identify existing knowledge gaps and provide direction for future research, as well as inform clinical approaches to the assessment and management of dissociative symptoms in depression and anxiety.

## 2 Methods

This scoping review was conducted to systematically map the existing literature on dissociation in depressive and anxiety disorders in adults. We followed the methodological framework outlined by the Joanna Briggs Institute (JBI) and adhered to the PRISMA Extension for Scoping Reviews (PRISMA-ScR) guidelines (18). The review protocol was developed in advance and served as the foundation for each stage of the review process. The protocol was not registered in PROSPERO but is publicly available on the Open Science Framework: https://osf.io/hnjpb/).

### 2.1 Eligibility criteria and search strategy

We included original research studies that examined dissociative symptoms in adults (aged 18 years and older) with a presence of depressive and/or anxiety symptoms. Eligible study designs encompassed quantitative, qualitative, and mixed methods approaches, including case series. To be considered, studies had to assess dissociation using clinical interviews or validated instruments such as the DES.

Given the substantial phenomenological and clinical overlap between dissociative, depressive, and anxiety symptoms, studies conducted in trauma-exposed populations were considered eligible for inclusion provided that depressive and/or anxiety symptoms were explicitly assessed alongside dissociative symptoms. This approach was chosen to reflect real-world clinical presentations, in which trauma exposure frequently co-occurs with affective and anxiety symptomatology rather than constituting an isolated diagnostic entity. In contrast, studies focusing exclusively on trauma-related outcomes without formal assessment of depressive or anxiety symptoms were excluded, as they did not address the core objective of the present review, namely the relationship between dissociative phenomena and depressive and/or anxiety symptom dimensions. Similarly, studies conducted in populations experiencing situational or stress-related conditions (e.g., occupational burnout) were included when depressive and/or anxiety symptoms were explicitly assessed alongside dissociative phenomena, reflecting the transient and context-dependent nature of dissociative experiences in response to sustained stress exposure.

We excluded studies focusing primarily on bipolar disorder, psychotic disorders, borderline personality disorder, or substance use disorders.Studies involving only children or adolescents, as well as case reports and narrative reviews, were also excluded. Systematic reviews and meta-analyses were excluded as primary sources, with the exception of a small number of syntheses providing prevalence data not available from individual studies, which were retained and are identified as such in the Results

We systematically searched PubMed and Scopus from inception to 01.2025, with language restriction to languages known by the authors. The search strategy, developed in collaboration with a research librarian, combined controlled vocabulary (e.g., MeSH terms) and free-text terms related to dissociation, depression, anxiety, and treatment outcomes. We also screened the reference lists of included studies and relevant reviews for additional sources. The full search strategy is provided in **Supplementary Material 1**.

### 2.2 Study selection

**Figure 1:**
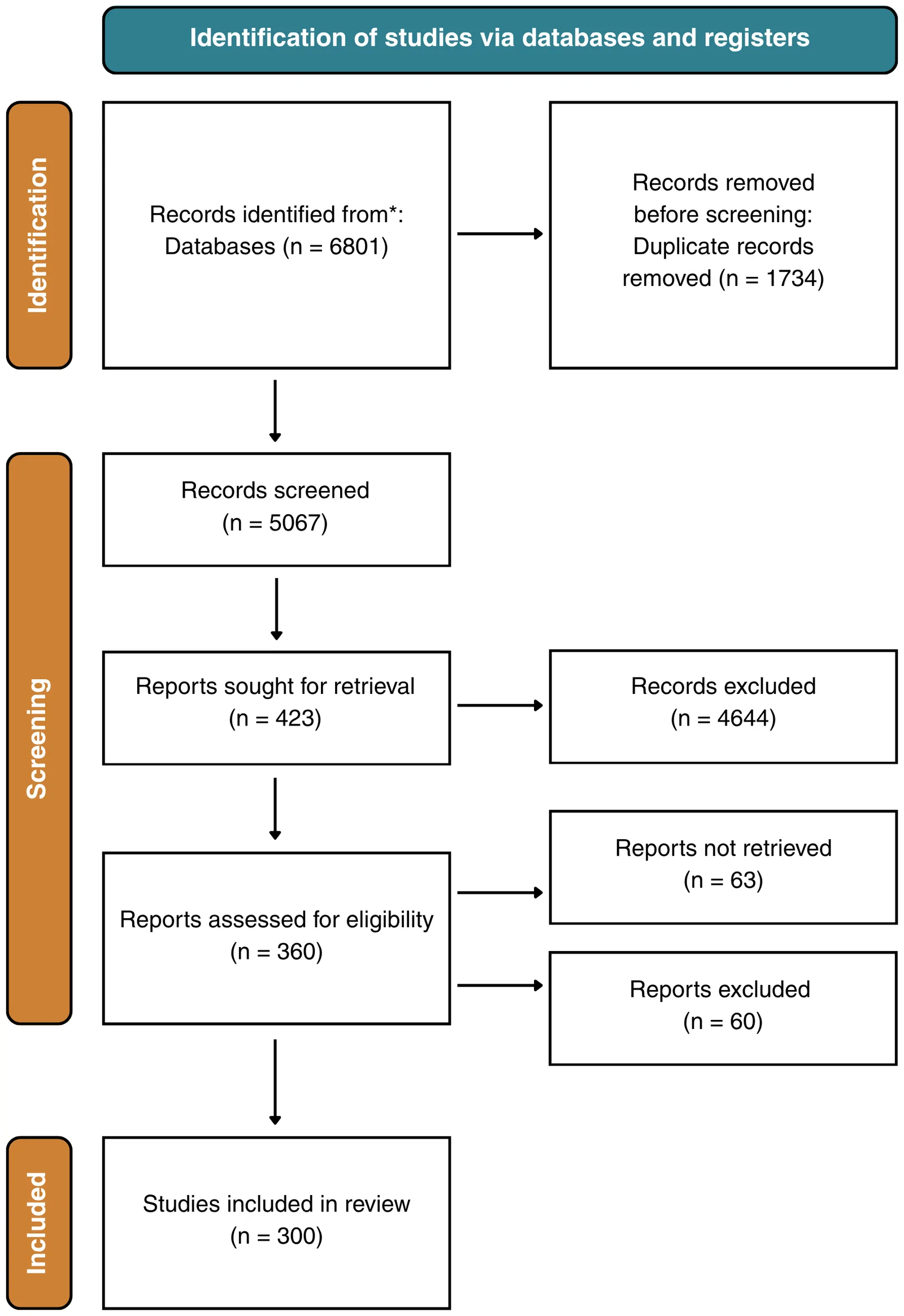
Study selection process (PRISMA-ScR flow diagram).

We imported all search results into Rayyan (Rayyan Systems Inc.) and removed duplicates (19). Two reviewers independently screened titles and abstracts to identify studies that met the inclusion criteria. Full texts of potentially eligible studies were then assessed in detail. Any disagreements were resolved through discussion, with arbitration by a third reviewer when necessary. We present the study selection process in a PRISMA-ScR flow diagram (**Figure 1***).*

### 2.3 Data extraction

We extracted data using a structured form developed in Microsoft Excel, based on the predefined variables outlined in our protocol. For each study, we recorded the same set of variables (**Table 1**). Two reviewers independently extracted the data. Discrepancies were resolved through discussion, with input from a third reviewer as needed.

**Table 1:** Data charting form: variables extracted from the included studies.

Main category	Subcategories
Publication details	Authors Title Year of publication Journal Country of origin
Study characteristics	Study type Study design Primary objective Secondary objective
Participant characteristics	Number of participants Age Gender Nationality Diagnostic information
Intervention characteristics (where applicable)	Type of intervention (e.g., pharmacotherapy, psychotherapy, or both) Duration of treatment or exposure Follow-up period Use of comparator Participant adherence Assessment tools
Outcomes	Rate of symptom improvement
Findings	Main findings Secondary findings
Study limitations	Reported limitations
Authors’ recommendations	Recommendations for clinical practice and/or future research

### 2.4 Data synthesis

We organized the findings thematically, focusing on how dissociation was defined and assessed, its reported prevalence and severity, and its relationship to symptomatology and treatment outcomes in depressive and anxiety disorders. While a symptom-based framework guided study eligibility and conceptual inclusion decisions, the synthesis and presentation of results were organized using clinically recognizable diagnostic groupings corresponding primarily to DSM-5 categories. This strategy was adopted for pragmatic and interpretative clarity given the size and heterogeneity of the included literature. Accordingly, results were structured into four main groups: DPDR, DID/pDID, FND, and mixed dissociative presentations encompassing studies that did not fit exclusively within a single diagnostic category. Although FND is classified in DSM-5 under somatic symptom and related disorders rather than dissociative disorders (1), it has been historically conceptualized as a dissociative condition and is classified within the dissociative [conversion] disorders in ICD-10 (20) and ICD-11 (21). Moreover, dissociative mechanisms are central to several contemporary theoretical and neurobiological models of FND, justifying its inclusion as a distinct category within the present review (22, 23).

DSM-5 diagnostic categories were applied at this stage solely as an organizational framework and did not constitute a diagnosis-driven analytic approach. Although ICD-11 offers a more explicitly dimensional conceptualization of dissociative phenomena, DSM-5 groupings were better suited to structuring results into clinically interpretable categories while preserving the review’s symptom-focused analytic framework (20, 21).

## 3 Results

**Figure 2:**
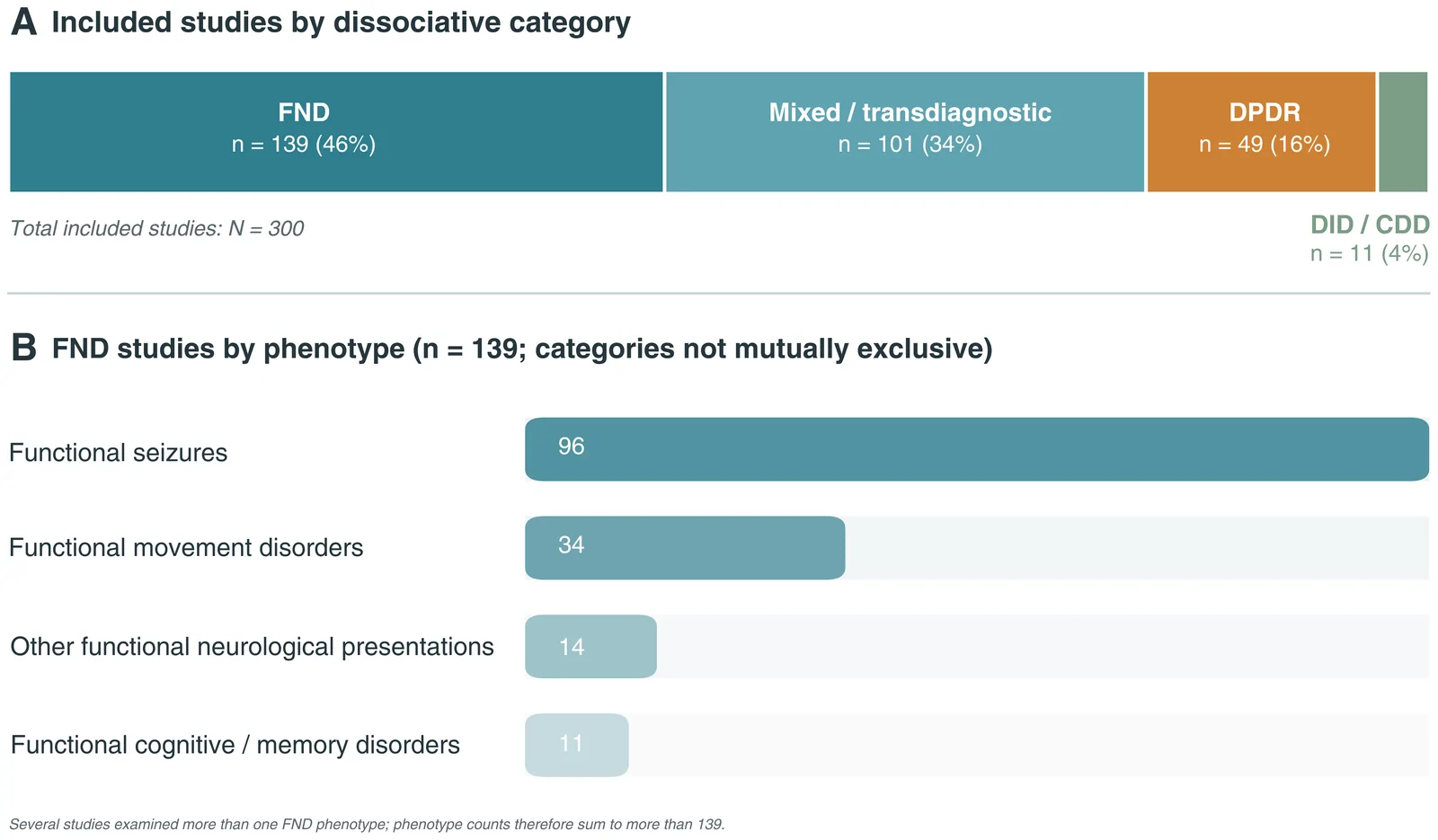
Evidence map of the included literature. **(A)** distribution of included studies across the four dissociative categories. **(B)** distribution of FND studies by phenotype; categories are not mutually exclusive, as several studies examined more than one phenotype.

Our selection of studies is summarised in the PRISMA Flow Diagram following guidelines by Page et al. (24) as shown in **Figure 1**. Our primary search led to the identification 6801 papers, from which 1734 were removed as duplicates. Following this procedure, 423 were selected based on abstract review. Eventually, 360 were included in full-text review, with 300 retained in the final review. 49 studies were identified as belonging to the DPDR category, 11 to DID and CDD, 139 to FND and 101 to Mixed/Other Dissociative Phenomena (**Figure 2**). Reported prevalence estimates, with the corresponding source studies, instruments, diagnostic cut-offs, and sample types, are summarized in **Table 2**.

**Table 2:** Traceability of reported prevalence estimates: mapping of prevalence bands and point estimates to source studies, instruments, diagnostic cut-offs, and sample types.

Band/single % value	What it estimates	Ref.	Instruments	Cut-off	Sample
DPDR					
41–43%	Comorbid anxiety disorder	(25, 26)	self-reported dx (25); clinical dx (ICD-10 F40–F41), severity via GAD-7 (26)	DSM-IV (25); ICD-10 F40–F41 (26)	204 DP-disorder cases; 41% (82/204) (25); 223 DDS outpatients, 42.6% (95/223) (26)
62%	Comorbid depression (self-report)	(25)	Self-reported dx; BDI (mean 22.0, moderate)	DSM-IV; BDI	204 DP-disorder cases, London
84.8%	Comorbid depressive disorder	(26)	Clinical dx (ICD-10); severity via PHQ-9	ICD-10 F32–F34; PHQ-9 ≥10	223 DDS outpatients, Mainz (125 M: 98 F)
80.3%	DP/DR during panic states	(27)	Semistructured interview (yes/no)	DSM-III-R panic disorder w/agoraphobia	49/61 agoraphobic PD patients
1–2%	Clinically significant DP/DR, general population	(28)	Standardized diagnostic interviews (systematic review)	Diagnostic-interview threshold	Community surveys (UK 1.2–1.7%; Canada 2.4%)
30–60%	DP/DR within specific psychiatric disorders	(28)	Clinical surveys/screening (systematic review)	Varies	30% PTSD war veterans, 60% unipolar depression
47.3%	≥1 moderate DP symptom under academic stress	(29)	Self-rated DP after oral exam	≥1 moderate symptom	203 students (Study 1)
DID/CDD					
57.1–72.7%	Panic syndrome	(30)	Mini-DIPS; DD dx via SCID-D-R	DSM-IV	DID (n=44) 72.7%/DDNOS (n=22) 57.1%, German women
47.6–50%/23.8–36.4%/27.3–38.1%	Phobias by type: agoraphobia/specific/social	(30)	Mini-DIPS	DSM-IV	DID/DDNOS
19.0–20.9%	OCD	(30)	Mini-DIPS	DSM-IV	DID 20.9%/DDNOS 19.0%
20%	Any anxiety disorder	(31)	DES (>30) + DIS-Q; diagnosis per DSM-5 (without DDIS/SCID)	DSM-5	Turkish DID outpatient series
44.3%	Any anxiety (prior dx)	(32)	Clinician-reported dx, case-series survey	DSM-III (MPD)	236 MPD cases/203 clinicians
90.5–98%	PTSD	(30)	Mini-DIPS	DSM-IV	DID 97.7–98%/DDNOS 90.5–91%
63.4% (lifetime)/~29% (point)	Major depression	(30)	Mini-DIPS (point); clinical history (lifetime)	DSM-IV	MDD group (DID+DDNOS); point: DID 29.5%/DDNOS 28.6%
63.7%	Affective disorders (prior dx)	(32)	Clinician-reported dx	DSM-III	236 MPD cases
FND					
40–70%	Clinically significant anxiety symptoms/anxiety disorders in FS	(33–35)	HADS-A, STAI, HAM-A, DSM-based clinical diagnosis (varied)	Study-specific	Adult FS cohorts; epilepsy centers
35–65%	Clinically significant depressive symptoms/depressive disorders in FS	(33–38)	BDI, BDI-II, HADS-D, HAM-D	Study-specific	Adult FS cohorts; epilepsy centers
25–50%	Co-occurring anxiety and depression	(33–34, 35)	Combined psychiatric assessment	DSM/ICD diagnosis	Adult FS cohorts; epilepsy centers
40–60%	Anxiety and/or depressive symptoms in functional movement disorders (FMD)	(39–72)	HADS, BDI, STAI	Study-specific	Adult FMD cohort
30–60%	Elevated dissociative symptoms (DES)	(73–81)	DES/DES-II	DES total score (usually ≥20–30 depending on study)	Mixed FND cohorts (predominantly FS)

### 3.1 Depersonalization and derealization (DPDR)

We identified 49 studies focusing on depersonalization and derealization phenomena, encompassing DPDR as well as depersonalization symptoms occurring across a range of clinical and non-clinical contexts. These included anxiety disorders and panic disorder (82, 83), psychosomatic and consultation–liaison settings (84, 85), dissociative and functional neurological conditions, including functional seizures (FS) (86, 87), chronic pain syndromes such as complex regional pain syndrome (88), and stress-related states, particularly academic stress (29) and high-demand occupational environments, where depersonalization was assessed as a burnout dimension rather than as a dissociative symptom (89). Several studies also addressed depersonalization in relation to internet use and digital environments (90). In addition, broader dissociation-focused literature (including clinical and community samples) has examined dissociative phenomena in relation to trauma exposure and post-traumatic stress disorder (PTSD)-focused treatment contexts (91–95), obsessive-compulsive disorder (OCD) and attachment/early-life experiences (96, 97), neurotic spectrum disorders and intensive inpatient psychotherapy (including associations with dissociation and self-stigma) (98, 99), migraine and sensory symptoms (100), eating disorders (EDs) and treatment outcome mechanisms (101), and suicidal behaviour in relation to childhood trauma (102, 103), as well as in Lesbian, Gay, Bisexual, Transgender, and Queer (LGBTQ) populations exposed to trauma and discrimination (26, 104) and in college samples exposed to gendered sexual violence (105). Beyond DPDR-focused samples, this literature underscores the breadth of dissociation-relevant contexts in which depersonalization-like phenomena may be clinically salient, supporting transdiagnostic models in which dissociation emerges across a range of psychiatric and medical conditions under conditions of stress and trauma exposure (106–108).

The majority of studies originated from Europe (n = 36), particularly Germany and UK and other Western European countries (26, 28, 84, 85, 93, 106, 109–138), followed by Asia/Middle East and Australia (117, 131, 139–145) and North America (n = 6) (132, 146–148) (multiple sites possible). Related dissociation literature also includes work in culturally diverse samples, such as studies examining clinical features of pathological dissociation in a Chinese population (140). Furthermore, research in minority and trauma-exposed populations has highlighted the role of sociocultural stressors, including discrimination and gendered violence, in shaping dissociative symptomatology (26, 102).

The identified literature comprised epidemiological and systematic reviews examining the prevalence and distribution of depersonalization and derealization symptoms (28, 146, 149), neuroimaging investigations demonstrating structural brain alterations associated with depersonalization disorder (143, 150), and studies validating or applying assessment instruments for depersonalization and dissociation (119, 121, 122, 148). Complementary neurobiological work in closely related dissociative presentations has examined resting-state functional connectivity before and after inpatient trauma treatment in complex PTSD (cPTSD) and complex dissociative disorder samples (151, 152). Related psychobiological research has also explored stress-related biomarkers as predictors of treatment outcome in trauma-focused inpatient settings, providing an additional framework for integrating stress physiology into dissociation-adjacent models without implying disorder specificity to DPDR (91). Several studies explored illness perceptions, attribution styles, and cognitive-emotional mechanisms underlying depersonalization symptoms, including associations with anxiety, shame, stress, and altered emotional processing (29, 82, 84, 115, 153, 154). Related cognitive-emotional and clinical-correlational work has also been conducted in DID, including studies linking anxiety-related constructs to depressive severity (155) and investigations of emotion perception in relation to integration-related measures (156).

Both pharmacological and psychological intervention studies were identified. Pharmacological approaches primarily investigated the effects of serotonin reuptake inhibitors (SSRI) (157, 158), while psychological interventions predominantly involved cognitive-behavioural therapy (CBT) delivered in individual or group formats (115, 136, 159), as well as experimental paradigms using interoceptive exposure to evoke depersonalization and derealization experiences (160, 161). One consecutive case series additionally evaluated repetitive transcranial magnetic stimulation of the right ventrolateral prefrontal cortex in medication-resistant depersonalization disorder, using the Cambridge Depersonalization Scale as the primary outcome and depressive and anxiety symptoms as secondary outcomes (100). In adjacent dissociation- and trauma-focused intervention literatures, randomized and non-randomized clinical trials have compared Eye Movement Desensitisation and Reprocessing (EMDR) with trauma-focused cognitive behavioural therapy in women with childhood sexual abuse histories (162), evaluated skills training followed by EMDR or narrative therapy in adult survivors of childhood abuse (163), investigated therapist-delivered web-based EMDR in adults with suicidal ideation (164), and examined group EMDR approaches in recurrent interpersonal trauma (165), alongside open trials of trauma-focused psychodynamic psychotherapy in LGBTQ individuals with PTSD (166). Across clinical and non-clinical populations, depersonalization and derealization symptoms were consistently associated with heightened anxiety, stress sensitivity, dissociative tendencies, and increased attentional focus on internal bodily and perceptual experiences (29, 82, 106, 167), supporting their transdiagnostic and stress-responsive nature.

#### 3.1.1 Study designs and samples

Sample characteristics varied widely, ranging from case series and small patient cohorts (25, 26) to larger samples of students (29, 159), community-dwelling adults (118, 149), and clinical populations with anxiety disorders (168), panic disorder (27), psychosomatic conditions (84), functional neurological symptoms (169), or chronic pain syndromes (108).

#### 3.1.2 Characteristics of the study populations

The 49 included studies employed heterogeneous methodological designs and sampled both clinical and non-clinical populations. Study designs comprised clinical case series and descriptive cohort studies (25, 26, 84, 154, 170) conducted in psychosomatic, psychiatric, and consultation-liaison settings, as well as cross-sectional survey studies in community and student samples (29, 82, 118, 149, 171, 172) examining the prevalence and correlates of depersonalization and derealization symptoms. One study was a systematic review synthesizing epidemiological data on depersonalization and derealization (28). Complementary dissociation-focused clinical epidemiology and characterization work has been reported in DID and pathological dissociation samples (31, 32, 173, 174).

Across studies focusing on depersonalization and derealization, samples were dominated by young and middle-aged adults, with symptom onset and highest prevalence typically reported in adolescence and early adulthood, most commonly between 20 and 40 years of age (28, 170). Clinical cohorts with primary DPDR generally reported mean ages in the late twenties to mid-thirties (25, 26, 115, 170), while psychosomatic and consultation–liaison samples extended into the fourth and fifth decades of life (84, 169). In contrast, studies centred on depression and/or anxiety, including those assessing depersonalization as a comorbid or dimensional phenomenon, tended to include broader adult age ranges, often with mean ages in the mid-thirties to late forties (82, 106, 107, 168). Student and academic stress samples represented a younger subgroup, with mean ages in the early twenties, in which depersonalization closely co-occurred with anxiety and depressive symptoms (29, 109, 159). Dissociation-related research in other clinical populations has similarly included broad adult age ranges, including inpatient PTSD samples (91, 92), college samples exposed to trauma (29) and adult DID cohorts followed longitudinally (91, 92).

Across studies of depersonalization and derealization, samples showed a balanced or only mildly female-predominant sex distribution, with several clinical and epidemiological studies reporting near-equal proportions of women and men (25, 26, 28, 149). This contrasts with the clear female predominance typically observed in depression and anxiety disorders (106, 107). Depersonalization-specific clinical cohorts often included comparable numbers of male and female participants (25, 26, 170), whereas psychosomatic and student stress samples showed a modest female predominance, likely reflecting referral and sampling patterns rather than disorder-specific risk (29, 84, 159). In broader dissociation research, sex distributions vary by population and recruitment context, including trauma-exposed college samples (29) and clinical DID samples (91). Overall, sex differences in depersonalization and derealization appear less pronounced than in affective disorders.

#### 3.1.3 Assessment tools

Across the 49 included studies, depersonalization and derealization were most commonly assessed using validated self-report instruments. The Cambridge Depersonalization Scale (CDS) was the most frequently applied measure, particularly in clinical depersonalization disorder samples, intervention studies, stress-related depersonalization in students, and neurobiological investigations (119, 121, 127, 170). The DES, including its German version, was used to assess dissociative symptoms and depersonalization-derealization severity in psychosomatic, epidemiological, and chronic pain populations (108, 125, 149, 169). In consultation-liaison and psychosomatic outpatient settings, depersonalization-derealization was often identified through clinical diagnostic assessment rather than a single standardized scale (25, 26, 84, 170). Broader dissociation assessment practices have been addressed in the context of DID, including dedicated discussions of psychological assessment approaches (175).

Measures of anxiety were frequently included, most commonly the State-Trait Anxiety Inventory (STAI) in studies examining panic-related depersonalization, academic stress, and cognitive-behavioural interventions (27, 29, 109, 159). Panic disorder-specific diagnostic criteria or symptom assessments were applied in studies focusing on panic and hypnosis (27, 168), while social anxiety and shame were examined using dedicated psychometric tools in one study (153). Related work in DID has examined anxiety-related constructs such as anxiety sensitivity in relation to depressive severity (155), and clinically oriented research has explored factors that may complicate treatment response in panic-focused CBT (176, 177).

Depressive symptoms were most often assessed using the Beck Depression Inventory (BDI/BDI-II), particularly in clinical depersonalization disorder cohorts, pharmacological treatment studies, and cognitive-behavioural interventions (136, 157, 170). Several additional studies captured depression using subscales embedded within broader psychosomatic or stress-related symptom inventories (29, 82, 149).

Dissociation-related constructs beyond depersonalization were evaluated using the DES total score in studies of functional seizures and psychosomatic populations (125, 149, 169), while hypnotizability scales were employed in experimental studies examining depersonalization during panic and hypnosis (27, 169). Stress exposure and psychosocial factors were assessed using academic stress questionnaires, stress inventories, or symptom-based measures in student and clinical samples (29, 82, 109, 159), and illness perceptions were specifically examined in one study testing an illness attribution model in depersonalization disorder (153). In other clinical domains, dissociation has been examined alongside constructs such as self-stigma and personality disorder comorbidity, trauma-related distress (98, 99), and mediators of suicide attempts in the context of childhood trauma (103).

#### 3.1.4 Conceptual approaches to dissociation

Across the reviewed studies, dissociation was conceptualized through partially overlapping frameworks, including anxiety-based models linking depersonalization to panic and autonomic arousal (27, 83, 109, 168), cognitive-behavioural models emphasizing maladaptive attentional focus and catastrophic misinterpretation of internal experiences (115, 153, 178), and stress-informed perspectives viewing dissociation as a response to chronic stress or emotional overload (29, 109, 118, 149). Epidemiological work supported a dimensional, transdiagnostic conceptualization, while neurobiological studies highlighted alterations in self-processing and emotion regulation networks, framing dissociation as a disorder of integrated self-experience rather than a unitary diagnostic entity (28, 118, 149, 150, 152, 179). Related transdiagnostic and stress- and trauma-informed perspectives are reflected in studies examining dissociation alongside PTSD symptomatology and trauma exposure in diverse clinical and non-clinical samples (92, 118), and in work linking dissociation to functional and clinical outcomes across other psychiatric and medical conditions (91, 118).

#### 3.1.5 Depersonalization and derealization in relation to anxiety and depression

Across the 49 included studies, depersonalization and derealization showed a strong and consistent association with anxiety and depressive symptoms, both in clinical and non-clinical populations. However, a significant heterogeneity in the relationship between DPDR and anxiety and/or depression has to be noted. This is due to the fact that in the majority of studies, DPDR has a fluctuating course independently of the established affective psychopathology. Anxiety disorders, particularly panic disorder, social anxiety, and high trait anxiety, were the most frequently reported comorbid conditions. In clinical samples of patients with depersonalization disorder or prominent depersonalization symptoms, approximately 41-43% of patients met criteria for at least one anxiety disorder (41% in a series of 204 cases and 42.6% in a series of 223 cases (25, 26), with anxiety disorders also frequently reported in other series, though aggregate rates were not consistently provided (170). In studies explicitly examining panic disorder, depersonalization and derealization were reported in approximately 80% of patients (49 of 61) during panic states (27), consistent with the wide range (7.8–82.6%) reported across panic-disorder samples in an epidemiological review (28), and their presence was associated with an earlier onset of panic disorder and greater functional impairment (83). Related findings linking anxiety-related constructs to depressive severity have also been reported in DID populations (155).

Depressive disorders were also highly prevalent: 62% of a series of 204 depersonalization-disorder cases reported comorbid depression (25), rising to 84.8% in a larger outpatient series of 223 (26). In cohorts with a primary diagnosis of depersonalization disorder, mean depression scores fell in the moderate range and exceeded population norms even when full criteria for major depressive disorder were not met (25). In the large clinical case series of 117 patients with depersonalization disorder, comorbid depression was described as one of the most frequent additional diagnoses, alongside anxiety disorders (170). Comorbid affective symptoms have similarly been discussed in dissociation-focused clinical cohorts and follow-up studies in DID (180–182).

Epidemiological and population-based studies further underscored this association. In the systematic review of depersonalization and derealization epidemiology, depersonalization or derealization symptoms affected approximately 1-2% of the general population, whereas prevalence rose to 30-60% within specific psychiatric disorders, ranging from 30% in war veterans with PTSD to 60% in unipolar depression (28). Population surveys also demonstrated that depersonalization symptoms clustered with higher levels of anxiety, depression, and stress-related complaints, particularly among younger adults and individuals with problematic internet use or high psychosocial load (149, 171). Related population-based work has examined dissociation in other community and college contexts involving trauma exposure and stress-related psychopathology (105, 174, 183).

Stress-related samples provided converging evidence. In high academic stress conditions, 47.3% students reported at least one moderate depersonalization symptom following an oral examination, with symptom severity positively associated with test anxiety (29). In randomized and non-randomized intervention studies targeting high trait anxiety, depersonalization severity decreased in parallel with reductions in anxiety and depressive symptoms, supporting a functional and potentially reversible relationship between these domains (159, 184, 185). In adjacent literatures, trauma-focused interventions including EMDR, trauma-focused CBT, narrative therapy, and trauma-focused psychodynamic approaches have been tested in PTSD and trauma-exposed populations (162, 163, 165), including LGBTQ samples (166).

Several studies also highlighted overlapping psychological risk factors for depersonalization, anxiety, and depression. Elevated anxiety sensitivity, maladaptive coping styles, shame, and negative illness perceptions were associated with higher depersonalization severity (153, 169). In patients with functional neurological symptoms, including FS, depersonalization frequently co-occurred with anxiety and depressive symptoms, suggesting shared mechanisms across dissociative, affective, and functional presentations (87, 169). Related work has linked dissociation to clinical severity and psychosocial correlates in other diagnostic groups, including neurotic spectrum disorders (98, 99), and to emotion dysregulation, attachment insecurity, and PTSD symptoms in LGBTQ adults exposed to trauma and discrimination (186).

Overall, the available evidence indicates that depersonalization and derealization are rarely isolated phenomena. Instead, they are most commonly embedded within broader anxiety-depression spectra, with prevalence and severity increasing markedly in the presence of panic, chronic stress, and affective dysregulation. These findings support conceptualizations of depersonalization as a transdiagnostic stress- and anxiety-related phenomenon rather than a stand-alone affective disorder. Evidence from broader dissociation-focused literatures spanning DID clinical characterization and treatment development, trauma and PTSD intervention research, and studies linking dissociation to outcomes across other clinical and medical domains is consistent with the view that dissociative symptoms can be meaningfully situated within transdiagnostic, stress- and trauma-responsive frameworks.

### 3.2 DID and CDD

We identified 11 studies focusing on DID and CDD, including samples described as DDNOS/Other Specified Dissociative Disorder, Type 1 (OSDD-1); the former is DSM-IV-TR terminology and the latter its DSM-5 successor (1, 187), and the labels are reported as used in the original studies, and trauma-related complex presentations, such as cPTSD with dissociative features (30–32, 151, 155, 156, 175, 181, 182, 188, 189). For the purposes of this review, studies were grouped under the DID/CDD category when dissociative identity fragmentation or complex dissociative pathology constituted the primary clinical focus, even if samples were variably labelled as DID, DDNOS/OSDD-1, CDD, or cPTSD with dissociative features. The included literature addressed DID/CDD in highly clinical contexts, most commonly in specialized inpatient, residential, or trauma-focused programs, including a residential eating disorder (ED) treatment setting integrating trauma-focused care for patients with DID and ED comorbidity (188), and phase-oriented inpatient trauma treatment samples including CDD and cPTSD presentations assessed longitudinally with neurophysiological markers (151). One study focused on diagnostic characterization and assessment practices in inpatient DID/DDNOS cohorts, highlighting the need for multimethod evaluation to distinguish dissociative pathology from primary mood, anxiety, or psychotic disorders (175). Additional work examined symptom correlates and mechanisms in DID, including anxiety sensitivity and depression as cognitive-affective correlates of dissociative symptom severity (155), as well as integration-related markers linked to depression/PTSD burden and socio-cognitive functioning in web-based DID+PTSD cohorts (156).

The majority of studies originated from North America, including the United States-based clinical and web-based cohorts (155, 156, 175, 181, 182, 188) and a large clinician-reported Canadian case series (32). European studies included a German structured-interview comorbidity study in women with DID/DDNOS (30) and an EEG resting-state connectivity study conducted in Swiss and German inpatient trauma-treatment samples including CDD/cPTSD presentations (151). Additional evidence was contributed by an observational clinical characterization study from Turkey (31) and a single-case experimental psychotherapy study from Iran (189).

#### 3.2.1 Study designs and samples

Across the included studies, designs ranged from large descriptive clinician-reported case series (32) and inpatient psychological assessment work with empirical illustration (175), through structured-interview cross-sectional comorbidity profiling in clinical outpatient samples (30) and observational demographic/clinical characterization (31), to web-based cross-sectional studies examining integration-related correlates (156) and mechanistic correlates of anxiety sensitivity, depression, and dissociation (155). Two longitudinal follow-ups tracked inpatient DID cohorts over two years with outcomes stratified by integration status (181, 182), and one neurobiological longitudinal study examined resting-state EEG connectivity and symptom change before and after phase-oriented inpatient trauma treatment in CDD/cPTSD samples (151). Intervention-oriented evidence included a retrospective pre–post residential treatment case series integrating trauma-focused care for DID+ED comorbidity (188) and a single-case experimental psychotherapy study evaluating the Unified Protocol with follow-ups up to 6 months (189). Notably, the evidence base is dominated by clinical samples and specialist treatment settings (30, 32, 151, 175, 181, 182, 188), with no community-based epidemiological studies. As a result, the available data primarily inform clinical profiles, comorbidity patterns, assessment approaches, and treatment-associated change rather than population prevalence estimates.

#### 3.2.2 Characteristics of the study populations

When it comes to setting, the majority of studies (n = 6) were conducted in specialist psychiatric inpatient or residential treatment programs, including residential ED treatment with integrated trauma therapy (188), inpatient trauma treatment units (151), and inpatient psychiatric cohorts (32, 175, 181, 182). Three studies recruited from outpatient clinical settings (30, 31, 189). One large cross-sectional study was conducted through web-based recruitment of patients meeting self-report diagnostic thresholds for DID and PTSD (156). Finally, one United States (U.S.) observational study examined a DID cohort in which participants were recruited from clinical programs, but the specific setting (inpatient versus outpatient) was not clearly specified (155). No community-based studies were identified.

Across DID/CDD studies reporting age, samples largely comprised adults spanning early adulthood to midlife. Mean ages clustered around the early-to-mid 30s in inpatient/residential cohorts (32, 175, 188), while outpatient, web-based, and mechanistic cohorts often reported mean ages around the early 40s (151, 155, 156, 181, 182). One observational outpatient sample from Turkey included a younger mean age (31).

Sex distribution was predominantly female across most DID/DDNOS samples, including inpatient assessment cohorts (175), large clinician-reported series (32), longitudinal follow-up samples (181, 182), structured-interview clinical cohorts (30), and web-based DID+PTSD recruitment (156). A more mixed distribution was reported in the Turkish clinical sample (31) and in the Swiss/German inpatient neurobiological cohort (151).

Studies have shown that DID/CDD often present through other clinical pathways, such as ED residential programs or general inpatient psychiatry, and may go unrecognised for a long time without systematic dissociation-focused assessment (32, 188). Comprehensive, multimethod evaluation is often needed to differentiate dissociative pathology from primary affective, anxiety, or psychotic-spectrum presentations (175).

#### 3.2.3 Assessment tools

Dissociation was assessed with structured diagnostic interviews in three studies: the Structured Clinical Interview for Dissociative Disorders (SCID-D) (30, 175) and the Dissociative Disorders Interview Schedule (DDIS) (182). The majority of studies relied on self-report measures, including the DES (30, 31, 182), the Dissociation Questionnaire (DIS-Q) (31), the Multidimensional Inventory of Dissociation (MID) (155), SDQ-20 (151), and the Multiscale Dissociation Inventory (MDI) (156). This combination of structured diagnostic interviews and self-report instruments reflects the need for multimethod assessment in DID/CDD, given the risk of misclassification as primary mood, anxiety, or psychotic disorders when dissociation is not systematically evaluated.

Measures of anxiety and depressive symptoms were frequently included across the identified studies, most often as secondary endpoints reflecting overall clinical severity rather than primary treatment targets. The BDI/BDI-II was the most commonly used tool, appearing in six studies (30, 151, 155, 182, 189). The Hamilton Depression Rating Scale (HDRS/HAM-D) was applied in one longitudinal follow-up (182). The Beck Anxiety Inventory (BAI) was used in one intervention study (189), while the STAI appeared in two studies (151, 188).

Additionally, trauma exposure was systematically documented in most cohorts, most frequently with the Childhood Trauma Questionnaire (CTQ (30, 151, 155, 156);), and in one study with the Life Events Checklist for DSM-5 (LEC-5) (188);.

#### 3.2.4 Conceptual approaches to dissociation

Across the included studies, dissociation in DID and CDD was conceptualized primarily as a trauma-related and clinically organizing phenomenon rather than a transient or isolated symptom. Most investigations treated dissociation as a core feature defining the clinical presentation, with DID/DDNOS or CDD constituting the principal diagnostic framework and shaping patterns of comorbidity, assessment, and treatment course (30, 32, 181, 182). Within this perspective, dissociation was closely embedded in contexts of chronic childhood trauma and associated with extensive affective, anxiety, and post-traumatic symptom burden, while remaining only partially reducible to these domains. Several studies further emphasized dissociation as a process unfolding over time, operationalized through the construct of integration or self-cohesion, with higher levels of integration consistently associated with lower depression and PTSD severity and broader clinical improvement (156, 181, 182). Other work approached dissociation from a cognitive-affective perspective, highlighting indirect pathways linking anxiety-related vulnerability- particularly anxiety sensitivity- to depressive symptoms, which in turn were associated with dissociative symptom severity (155). Finally, phase-oriented inpatient trauma-treatment models conceptualized dissociation as relatively treatment-resistant compared with mood and PTSD symptoms, showing improvements in depression and post-traumatic stress alongside persistent dissociative features (151). Together, these approaches frame dissociation in DID/CDD as a central, trauma-organized dimension of psychopathology that intersects with, but is not subsumed by, anxiety and depressive processes.

#### 3.2.5 DID/CDD in relation to anxiety and depression

Across the available DID/CDD literature, anxiety and depressive disorders emerge as highly prevalent and clinically salient comorbidities rather than ancillary features, shaping both baseline severity and longitudinal outcomes. In DID/CDD samples, depressive disorders and anxiety-spectrum comorbidity were pervasive and clinically prominent (30–32, 188). Available evidence further suggests that mood and trauma-related symptoms often improve more readily with treatment than dissociative symptomatology itself in complex dissociative presentations (151).

Across DID cohorts, comorbidity with anxiety disorders was consistently high. In the German sample, panic syndrome was diagnosed in 57.1–72.7% (DID 72.7%, DDNOS 57.1%), with agoraphobia in 47.6–50%, specific phobia in 23.8–36.4%, social phobia in 27.3–38.1%, and OCD in 19.0–20.9% (30). In the Turkish outpatient series, 20% of DID patients were diagnosed with an anxiety disorder (31). The large Canadian survey reported 44.3% with anxiety disorders overall (32). PTSD was nearly universal, with 90.5–98% of patients meeting criteria (DID 97.7–98%, DDNOS 90.5–91%) (30).

Depressive disorders were similarly common and frequently co-occurred with anxiety and trauma-related symptoms. In the German sample of women with DID/DDNOS, 63.4% had lifetime major depression, with a lower point prevalence (DID 29.5%, DDNOS 28.6%) (30). In the Turkish cohort, depression was diagnosed in 67.1% of DID patients (31). In inpatient follow-ups, depression was also very common, although exact percentages were not consistently reported (181, 182). In the Canadian multicenter clinician survey, 63.7% of DID patients had affective disorders (32).

High rates of self-harm and suicidality were consistently reported in DID/CDD samples and occurred in the context of severe comorbid depressive, anxiety, and trauma-related symptomatology. In the Canadian series, 72% had attempted suicide and 2.1% died by suicide (32). In the Turkish study, 30% reported self-mutilating behavior (31). In the two U.S. longitudinal inpatient follow-ups, suicidality was described as frequent, but percentages were not systematically reported (181, 182). Although these studies did not formally test causal pathways, the consistently high rates of self-harm and suicidality were observed alongside very high prevalence of major depression, anxiety disorders, and PTSD, suggesting that suicidality in DID/CDD is embedded within broader affective and anxiety-related clinical severity rather than representing an isolated phenomenon.

Risk factors for mood comorbidity were also documented. Severe and chronic childhood trauma was nearly universal: in the Canadian series, 79.2% reported sexual abuse and 74.9% physical abuse (32); in the Turkish cohort, 64% reported physical abuse, 49% chronic neglect, and 41% sexual abuse (31). In the U.S. residential ED cohort, all DID patients reported severe maltreatment histories (188). Insecure attachment patterns were also associated with higher risk of comorbidity (30). Beyond diagnostic comorbidity counts, one cross-sectional study highlighted anxiety sensitivity- particularly cognitive concerns (fear of losing cognitive control)- as a clinically salient correlate of depressive severity in DID. In this sample, depressive symptoms statistically mediated the association between anxiety sensitivity and dissociative symptom severity, supporting the relevance of targeting cognitive-affective vulnerability pathways alongside trauma-related symptoms in DID (155).

Across longitudinal and mechanistic studies, integration consistently emerged as a key prognostic marker differentiating trajectories of affective, anxiety-related, and dissociative symptom change in DID/CDD. Longitudinal follow-up studies showed that patients who achieved integration over two-year follow-up exhibited substantially greater reductions in depression, anxiety-related symptoms, and dissociation than non-integrated patients, with affective symptom levels in integrated individuals approaching nonclinical ranges (181, 182). Mechanistic evidence from a longitudinal inpatient trauma-treatment study further supported this dissociation between symptom domains: depression and PTSD severity improved alongside enhanced emotion regulation, whereas trait anxiety and dissociative symptoms showed little change, suggesting relative treatment resistance of dissociation compared with affective symptoms (151). Converging cross-sectional evidence from a web-based DID+PTSD cohort indicated that higher integration was associated with lower depression and PTSD severity, fewer autobiographical memory disturbances, and better socio-cognitive functioning (156).

Taken together, these findings indicate that in DID/CDD, depressive and anxiety symptoms are both highly prevalent and clinically consequential, yet closely intertwined with dissociative processes that appear to follow partially distinct trajectories of change.

### 3.3 Functional neurological disorders

FND accounted for the largest share of the included studies (139 of 300). This proportion reflects the volume of available empirical work in this field rather than a classificatory claim. In DSM-5, FND is classified under somatic symptom and related disorders, whereas ICD-10 and ICD-11, as well as a longstanding conceptual tradition, conceptualize it as closely related to dissociation (20, 21). We therefore present FND as a clearly bounded subsection and treat its findings as dissociation-adjacent rather than as a dissociative disorder in the strict nosological sense. Accordingly, the cross-category statements made in the Discussion draw on convergence across all four categories and are not based on FND data alone.

From the final selection of 139 included articles, the literature on FND covered a wide spectrum of functional neurological presentations (multiple FND phenotypes possible in one patient). The largest subgroup focused on FS, which were addressed in 96 studies (69.1%). These studies examined clinical phenomenology, psychiatric and neuropsychological profiles, psychodynamic formulations, illness perceptions, diagnostic differentiation from epilepsy, predictors of delayed diagnosis and outcome, and health-related quality of life, including frequent comorbidity with epilepsy, trauma exposure, and post-traumatic stress symptoms (73–75, 190–200).

Functional movement disorders (FMDs) constituted the second largest subgroup, represented in 34 studies (24.5%), including functional tremor, dystonia, gait disorders, and motor conversion symptoms (39–72). These studies focused on attentional and emotional processing, body-schema alterations, sensory processing and interoception, autonomic dysfunction (including impaired vagal tone), biological stress markers, neuroplasticity, cognitive performance and performance validity, and treatment outcomes following multidisciplinary rehabilitation, physical therapy, neuromodulation, and antidepressant treatment (39–72).

Functional cognitive and memory disorders were addressed in 11 studies (7.9%), while other functional neurological presentations- such as functional voice disorders, functional cough, globus symptoms, stroke mimics, and mixed FND cohorts- were included in 14 studies (10.1%) (45, 48, 201–204). As expected in a scoping review, diagnostic categories were not mutually exclusive, and several studies examined overlapping FND phenotypes.

Across FND subtypes, psychiatric and psychological mechanisms were prominently represented. Depression and/or anxiety were explicitly examined in 78 studies (56.1%) (41, 73, 191, 200, 205–207), while dissociation (psychoform and/or somatoform) was assessed in 61 studies (43.9%) (74–79). Alexithymia was examined in 18 studies (12.9%) (75, 76, 197, 208–213), trauma-related psychopathology or PTSD in 22 studies (15.8%) (199, 214–218), personality traits or profiles in 19 studies (13.7%) (213, 219, 220), attachment styles in 6 studies (4.3%) (210, 221, 222), and suicidal ideation or suicide attempts in 6 studies (4.3%) (200, 205, 206).

Treatment-focused research accounted for 52 studies (37.4%), evaluating a broad range of psychological, pharmacological, and interdisciplinary interventions (47, 214, 223–228). CBT in various formats was investigated in 27 studies (19.4%) (79, 229–232), psychodynamic or integrative psychotherapy in 9 studies (6.5%) (191, 214–216, 231, 233, 234), psychoeducational interventions in 6 studies (4.3%) (80, 223, 225), and multidisciplinary rehabilitation programmes in 18 studies (12.9%) (45, 228, 231, 235). Pharmacological treatments, primarily antidepressants and selective serotonin reuptake inhibitors, were examined in 6 studies (4.3%) (47, 77, 193, 236, 237), while neuromodulation approaches were reported in 3 studies (2.2%) (49, 238). Randomized controlled trials (RCTs) accounted for 6 studies (4.3%), including large pragmatic trials and smaller intervention studies (195, 226, 234, 239).

Contextual and health-system factors were examined in 38 studies (27.3%) addressing comorbid epilepsy (74, 217, 240, 241), 5 studies (3.6%) focusing on traumatic brain injury or post-concussion syndromes (217, 242), 4 studies (2.9%) involving veteran or military populations (243, 244), and 11 studies (7.9%) conducted in low-resource or non-Western healthcare settings (45, 244–246). The psychological impact of the COVID-19 pandemic on patients with FND was examined in one study (0.7%) (247).

Geographically, studies originated predominantly from Europe (82/139; 59.0%) and North America (34/139; 24.5%), with additional contributions from South America (9/139; 6.5%), Africa (7/139; 5.0%), and Asia or other regions (7/139; 5.0%) (245, 246, 248, 249), reflecting increasing international engagement with the classification, mechanisms, and treatment of FND across diverse healthcare contexts.

#### 3.3.1 Study designs and samples

The included studies employed a wide range of methodological designs, with a clear predominance of observational approaches, including retrospective and prospective cohort studies, cross-sectional observational designs, case–control comparisons, and descriptive clinical series (191, 192, 194, 250–253). A smaller subset of studies used interventional designs, including RCTs, non-randomized intervention studies, and pilot or feasibility trials. Controlled and randomized designs were primarily used to evaluate psychological and interdisciplinary interventions for FS and, to a lesser extent, motor functional neurological disorder, particularly in studies of CBT, psychoeducational interventions, psychodynamic or integrative psychotherapy, and standardized medical care (46, 80, 190, 195, 223, 224, 240). Observational and case–control studies constituted the majority of the literature, especially those examining clinical phenomenology, psychiatric comorbidity, dissociative symptoms, personality traits, alexithymia, attachment styles, stress physiology, autonomic function, illness perception, and cognitive or attentional processing in FS and FMDs (44, 73, 74, 191, 254). Many studies included comparison groups, most commonly patients with epilepsy, other neurological conditions, or healthy controls (74, 209, 241, 253). Samples were typically drawn from specialist neurology, epilepsy, neuropsychiatry, and psychosomatic medicine services, although several studies relied on routinely collected clinical data, registry-based cohorts, or single-centre outpatient samples (243–245, 248).

Sample sizes varied substantially, ranging from small exploratory cohorts and experimental paradigms (e.g. functional memory disorders, functional tremor, body-schema tasks, deception-based cognitive studies) (40, 48, 201, 250, 255) to large multicentre randomized trials, longitudinal cohorts, and multidisciplinary rehabilitation outcome studies (45, 195, 226, 228, 231, 239, 256). Across study designs, samples were consistently characterized by high rates of psychiatric comorbidity, most commonly mood disorders, anxiety disorders, trauma-related conditions, dissociative symptoms, and personality-related vulnerabilities (39, 73–75, 80, 191).

In studies on FND, samples were predominantly composed of adults, with symptom onset most frequently reported in late adolescence or early adulthood, often in the late teens or twenties (190). In contrast, comorbid affective conditions such as depression and anxiety- highly prevalent across FND cohorts- were reported across a broader adult age range, with mean ages commonly extending into the mid-thirties to late forties, particularly in psychosomatic, chronic disability, and veteran populations (44, 257). Overall, dissociative FND symptoms tended to emerge earlier, while affective symptoms often persisted or intensified across adulthood.

Across the reviewed literature, samples showed a marked female predominance, with women typically comprising approximately 60–80% of study populations, particularly in cohorts with FS and FMDs (190, 191). Sex was almost universally reported as a binary variable, and data on gender identity were not systematically collected, limiting conclusions regarding gender-specific mechanisms in FND-related dissociation.

#### 3.3.2 Characteristics of the study populations

Across the included literature, study samples were most commonly recruited from specialist epilepsy, neurology, neuropsychiatry, psychosomatic medicine, and tertiary referral services, reflecting the clinical contexts in which FND and FS are typically diagnosed and managed (74, 241, 251). FS cohorts frequently incorporated comparison groups of patients with epilepsy or pure epileptic seizures, enabling detailed examination of diagnostic features, psychiatric comorbidity, cognitive–emotional profiles, and illness perception across conditions (74, 241, 253). Sociodemographic characteristics varied across studies but generally reflected a predominance of women of working age, with many cohorts reporting chronic symptom duration, high levels of health-care utilization, and substantial functional impairment or disability (192, 194, 252). Several studies highlighted prolonged delays to diagnosis, recurrent emergency department attendance, and frequent inpatient admissions, particularly in FS and severe motor FND samples (206, 248).

#### 3.3.3 Assessment tools

Across the included studies, assessment of FND and associated psychiatric features relied on a combination of specialist neurological evaluation, structured psychiatric interviews, and validated psychometric instruments. Diagnostic confirmation of FS most commonly involved video-electroencephalography (video-EEG) monitoring combined with expert clinical diagnosis, whereas FMDs and other functional neurological presentations were diagnosed through detailed neurological examination and established clinical criteria (191, 250). Psychiatric diagnoses were frequently established using DSM-based clinical interviews, often supplemented by standardized self-report measures (73, 191).

Depressive symptoms were most commonly assessed using the BDI/BDI-II, the HDRS/HAM-D, and in several studies the Hospital Anxiety and Depression Scale- Depression subscale (HADS-D) (73, 191). Anxiety symptoms were evaluated using instruments such as the STAI, the Hamilton Anxiety Rating Scale (HAM-A/HARS), and the Hospital Anxiety and Depression Scale- Anxiety subscale (HADS-A), with a number of FS studies additionally assessing panic and anxiety-related symptoms using disorder-specific clinical criteria (73, 191). Alexithymia was consistently measured using the Toronto Alexithymia Scale (TAS-20) in FS and epilepsy comparison studies (208, 209), while broader psychopathology and personality traits were frequently assessed using instruments such as the Minnesota Multiphasic Personality Inventory-2 (MMPI-2) (219, 258). Somatization and dissociative features were captured using symptom inventories and structured clinical assessments tailored to functional and dissociative presentations (75–77).

In studies focusing on functional cognitive disorder and functional movement disorder, cognitive complaints were evaluated using standard neuropsychological screening tools (e.g. measures of attention, memory, and executive function), alongside experimental paradigms assessing response bias and symptom validity, including the Guilty Knowledge Task, as well as subjective cognitive symptom questionnaires (201). Several functional movement disorder (FMD) studies additionally incorporated physiological measures, such as heart rate variability and resting vagal tone, to assess autonomic function, as well as experimental tasks examining body schema representation, including the forearm bisection task (39, 40).

Treatment and outcome studies employed symptom-specific and functional outcome measures, including seizure-frequency diaries, functional disability scales, and health-related quality-of-life instruments, with repeated administration of affective symptom scales to assess response to CBT, psychoeducational interventions, integrative psychotherapy, and interdisciplinary treatment programmes (80). More recent studies also applied standardized affective measures longitudinally to capture changes in psychological burden during external stressors, including the COVID-19 pandemic, in patients with FND (247).

Severity of dissociative symptoms was systematically assessed in a substantial subset of studies, reflecting the central role of dissociation in FND, particularly in FS and mixed functional/dissociative seizure presentations. Dissociation was most commonly measured using validated self-report instruments, notably DES and its revised versions, which were widely applied to quantify both psychoform dissociation (e.g. depersonalization, derealization, absorption) and dissociative tendencies associated with seizure-like phenomena (73, 81). Several studies reported significantly higher DES scores in FS compared with epilepsy and healthy control groups, supporting the diagnostic and phenomenological relevance of dissociation in these populations (80).

In addition to global dissociation measures, some investigations employed broader psychopathology and personality inventories, such as the MMPI-2, which includes dissociation-related and conversion-relevant profiles, to characterize dissociative and somatoform symptom patterns in FS and conversion disorder cohorts (217, 258). Dissociative features were also examined in relation to trauma exposure, affect regulation, and alexithymia, with multiple studies demonstrating strong associations between dissociative symptom severity and comorbid anxiety, depression, and trauma-related psychopathology (199).

#### 3.3.4 Conceptualization of dissociation in studies on FND

Across studies of FNDs, dissociation was most commonly conceptualized as a disruption in the integration of motor, sensory, cognitive, and affective processes, consistent with its classification in ICD-11 as a dissociative disorder (190, 191). In this framework, dissociation is understood as a mechanism underlying functional neurological symptoms, particularly in FS and FMDs, rather than as a secondary or epiphenomenal feature.

Earlier psychodynamic and stress-based models emphasized the role of unconscious conflict, affective dysregulation, trauma exposure, and maladaptive coping strategies in the development and maintenance of dissociative neurological symptoms (191, 214, 234). Within these models, dissociation was frequently interpreted as a defensive response to overwhelming emotional states, with symptom expression serving an intrapsychic or interpersonal regulatory function.

Cognitive-behavioural formulations conceptualized dissociation as a learned and maintained alteration in attention, bodily awareness, and symptom expectation, reinforced through avoidance behaviours, illness beliefs, hypervigilance to bodily sensations, and anxiety-related mechanisms (79, 224, 232). In this context, dissociative experiences were viewed as dynamically interacting with cognitive appraisals and emotional responses, contributing to symptom persistence.

More recent neuropsychiatric, experimental, and biopsychosocial models adopted a transdiagnostic perspective, conceptualizing dissociation as a dimensional process linking affective symptoms, stress responsivity, and functional neurological manifestations rather than as a discrete categorical entity (39, 40, 43, 44, 259). These studies highlighted alterations in self-representation, interoception, body schema, emotion regulation, and autonomic functioning, suggesting that dissociation in FND reflects distributed disruptions across neural and psychological systems involved in self-monitoring and bodily control.

Overall, the reviewed literature indicates a shift from narrowly defined psychodynamic explanations toward integrated models that situate dissociation within a broader biopsychosocial and neurocognitive framework, emphasizing its role as a core, transdiagnostic mechanism in FND rather than a disorder-specific construct.

#### 3.3.5 FND in relation to anxiety and depression

Across the reviewed literature, anxiety and depressive disorders were highly prevalent across all major subtypes of FNDs, including FS, FMDs, functional cognitive and memory disorders, and mixed medically unexplained neurological symptoms. In FS cohorts, clinically significant anxiety symptoms or anxiety disorders were reported in 40-70% of patients, while depressive disorders or clinically relevant depressive symptoms were observed in 35–65%, often reaching moderate to severe levels (33–38). A substantial proportion of FS patients- estimated at 25-50%- presented with co-occurring anxiety and depression, indicating prominent affective multimorbidity rather than isolated diagnoses (33–38). In addition, this association between anxiety, depression and dissociation should be interpreted as co-occurring dimensions belonging to the same biopsychosocial model and not only co-occurring phenomena. Comparative studies consistently demonstrated higher rates of both anxiety and depression in FS than in epilepsy or other neurological control groups, supporting their disorder-specific relevance (33–38).

Elevated affective symptom burden was not confined to seizure-related presentations. In FMD cohorts, clinically significant anxiety and/or depressive symptoms were reported in 40-60% of patients and were associated with greater disability, autonomic dysregulation (including impaired vagal tone), altered body schema, and prominent subjective cognitive complaints, despite relatively preserved objective neurological function (39–72). Studies of functional cognitive and memory disorders similarly reported high rates of comorbid anxiety and depression, frequently exceeding those observed in neurological comparator groups and often linked to health anxiety, impaired emotional processing, and persistent symptom-related distress (260–262).

Across FND subtypes, higher prevalence and severity of anxiety and depression were most consistently observed in patients with chronic symptom duration, comorbid epilepsy, history of traumatic brain injury, persistent severe disability, and high psychosocial stress, all of which were associated with poorer functional outcomes and prognosis (192, 217, 241, 242, 252). More recent studies further indicated that affective symptom burden in FND populations is sensitive to external stressors, with increased prevalence and severity of anxiety and depression reported during the COVID-19 pandemic, accompanied by worsening of functional neurological symptoms.

Depressive symptoms were frequently described as moderate to severe, commonly co-occurring with generalized anxiety, panic symptoms, and heightened somatic and dissociative distress (191, 200, 205, 211). Anxiety disorders- particularly panic disorder and trauma-related anxiety presentations- were especially prominent in FS cohorts and were associated with earlier symptom onset, higher seizure frequency, and greater functional impairment (81, 191, 199, 200). Overall, the literature supports a pattern of affective multimorbidity, rather than discrete anxiety or depressive disorders, as a characteristic feature of FND.

Intervention studies reinforced the clinical relevance of these findings, as baseline severity of anxiety and depression was frequently associated with treatment response and long-term outcome, with higher affective burden associated with poorer prognosis in untreated or standard-care groups (192, 216, 222, 263). Conversely, psychological interventions- particularly CBT and integrative psychotherapeutic approaches- were associated with reductions in both functional neurological symptoms and comorbid anxiety and depressive symptoms, underscoring the modifiable nature of affective burden in FND (207, 214, 227, 239).

Across studies of FND, other dissociative symptoms- most extensively examined in FS- were consistently elevated compared with neurological and healthy control groups. Using standardized measures, most commonly DES, clinically significant dissociative symptoms were reported in 30-60% of FS patients, with a substantial subgroup meeting thresholds suggestive of pathological dissociation (73–81). Dissociative symptom severity was strongly associated with higher levels of anxiety and depression, trauma-related psychopathology, alexithymia, somatoform symptoms, greater seizure frequency, symptom chronicity, and functional impairment.

Beyond FS, dissociative features were also reported in FMDs and functional cognitive/memory disorders, although assessed less systematically. In these subgroups, dissociation was typically identified through clinical interviews, psychopathology inventories, or associated affective and somatic symptom profiles and was linked to subjective cognitive complaints, altered bodily self-representation, and poorer clinical outcomes. Taken together, the literature supports dissociation as a core transdiagnostic feature of FND, most prominent in seizure-related presentations but also relevant across motor and cognitive functional syndromes, with heightened expression under conditions of psychological stress, including during the COVID-19 pandemic.

### 3.4 Mixed dissociative phenomena

A total of 101 studies investigated dissociative phenomena across a wide range of clinical and non-clinical samples in relation to depressive and/or anxiety symptoms (2, 12, 13, 96–99, 101, 103, 105, 162–166, 173, 174, 176, 177, 183, 186, 232, 264–342). This category was retained to capture the substantial body of literature in which dissociation was examined in relation to depressive and/or anxiety symptoms without being confined to a single dissociative diagnosis or clearly delineated dissociative subtype. In these studies, dissociation was variably conceptualized as a symptom dimension, process, or comorbid feature across heterogeneous psychiatric, medical, and non-clinical contexts. Excluding this literature would have resulted in an artificial narrowing of the review scope and a distorted representation of how dissociation is most commonly operationalized and studied in relation to depression and anxiety. Importantly, PTSD-focused samples were included in this section not as a primary diagnostic category, but as a frequent empirical context in which dissociation and its associations with depressive and anxiety symptoms were systematically assessed.

The studies included in the review derived from various countries, with particularly high research activity observed in Central and Eastern Europe: most notably in the Czech Republic and Slovakia (n = 15) (96–99, 270, 272, 273, 288, 300, 301, 303, 326, 337, 339, 342) and Turkey (n=17) (12, 103, 174, 268, 283–285, 287, 293, 294, 296, 297, 316, 319, 331–333), alongside numerous studies from Western Europe: 10 from Italy (165, 266, 271, 299, 304, 306, 322, 325, 336, 338), 3 from Spain (101, 162, 165), 13 from Germany (2, 163, 267, 269, 279, 280, 295, 307, 314, 315, 323, 328, 341), 2 from the Netherlands (163, 318), 4 from the United Kingdom (232, 275, 308, 329), and 3 from Switzerland (282, 295, 324), North America: 21 from United States and Canada (105, 164, 166, 176, 177, 186, 264, 265, 278, 289, 309–313, 318, 320, 324, 327, 330, 340), and East and South Asia: 3 from China and Hong Kong (13, 173, 277), 1 from Japan (290), 1 from South Korea (276) 1 from India (335), 1 from Pakistan (183), and 1 from Iran (282). Individual studies also originated from Australia (281, 292, 305), Israel (274), Romania (298), Belgium (334), Brazil (302), Serbia (321), Sweden (286), Denmark (291), Austria (323), and South Africa (317).

#### 3.4.1 Study designs and samples

Study designs were heterogeneous across the 101 included studies, encompassing randomized, longitudinal, cross-sectional, and open-label approaches.

The majority of investigations (n=55) were cross-sectional or observational in design (12, 13, 97, 99, 103, 105, 173, 174, 183, 186, 264, 266, 268, 271, 272, 275, 276, 278, 281, 282, 284, 285, 287, 289–293, 295, 296, 299, 302, 305, 306, 311–314, 316, 317, 319, 321–323, 327–330, 332–338), typically assessing associations between dissociative symptoms and depression or anxiety in both clinical and community samples. A smaller subset of studies (n=22) employed longitudinal or prospective designs, examining the temporal dynamics of dissociation and affective symptoms or treatment outcomes (96, 98, 101, 267, 269, 270, 273, 274, 277, 286, 294, 300, 301, 303, 307, 320, 324, 326, 331, 339, 341, 342). Several papers (n=6) reported RCTs or structured treatment evaluations (162–164, 176, 177, 310), including trauma-focused and transdiagnostic interventions evaluating depression, anxiety, and dissociation concurrently. A group (n=16) of predominantly open-label (165, 166, 265, 288, 297, 325), case series (232, 280, 304, 309), experimental (283, 298, 308, 315, 318, 340), and pilot studies (165, 166, 265, 325) further explored dissociation within clinical or neurobiological contexts. Finally, a smaller number of studies (n=2) used mixed/psychometric validation (279) or meta-analytic (2) methodology typically focusing on dissociative measurement or interventional synthesis rather than primary empirical data. Importantly, these methodological categories were not mutually exclusive, as several studies employed hybrid designs, and therefore category counts should not be interpreted as summing to the total number of included studies.

#### 3.4.2 Characteristics of the study populations

Across studies classified under mixed dissociation, samples were highly heterogeneous, spanning psychiatric, medical, and community-based populations. Most studies recruited participants with affective and anxiety disorders, trauma-related conditions, or mixed psychiatric diagnoses, while a smaller subset examined dissociative symptoms in medical or non-clinical samples. This heterogeneity reflects the transdiagnostic positioning of dissociation in the included literature rather than discrete diagnostic boundaries.

Across the included studies, age characteristics were reported heterogeneously (means with or without standard deviations, medians, ranges, or subgroup-specific summaries), but the review primarily captured adult populations, in line with the predefined eligibility criteria. Most psychiatric clinical samples clustered in early-to-mid adulthood, with mean ages typically in the third to fifth decade of life, particularly in trauma-related, affective, anxiety, obsessive–compulsive, and neurotic spectrum disorder cohorts. Non-clinical samples, especially university student populations, were generally younger, with mean ages in late adolescence to early adulthood, whereas a smaller subset of studies focused on older or medically ill populations. Across studies reporting explicit age bounds, the reported age range extended from 14 to 88 years. The upper age limit reflected inclusion of older and medically ill cohorts, most notably patients following transient ischemic attack (269). Importantly, although several reports presented lower age limits below 18 years (14–15 years) (266, 290, 321), these studies did not constitute pediatric or adolescent cohorts. Rather, they represented adult-dominant samples in which the inclusion or reporting of a small minority of younger participants reflected broad recruitment frames or descriptive reporting practices. Accordingly, these studies were retained and interpreted within the scope of an adult-focused scoping review, and no included study explicitly targeted a child or adolescent clinical population.

Sex/gender reporting was also variable and often non-comparable across studies (binary sex ratios, percentage female, or broader gender identity categories). Overall, samples were female-predominant, and single-sex recruitment was common, including 19 all-female studies (101, 162, 165, 174, 267, 279, 285–287, 296, 304, 308, 309, 329–331, 334, 338, 340) and one all-male study (315). Many mixed-sex clinical cohorts still showed clear female overrepresentation, particularly in trauma-related and general outpatient samples. Several populations were male-dominant in line with the source population, most notably military/veteran/first responder PTSD (83.9% male) (264), transient ischaemic attack (TIA) (62.3% male) (269), and U.S. Army soldiers with first documented major depressive disorder (MDD) (78% male) (327); male-majority patterns also appeared in some psychiatric outpatient samples (305, 313, 325) and one MDD cohort (282). A minority of studies explicitly reported gender-diverse identities (e.g., inclusion of non-binary and transgender participants in suicidal ideation treatment trials and LGBTQ samples) (13, 105, 164, 166, 186, 278), while one internal medicine inpatient study lacked baseline gender data (291) and one meta-analysis did not specify gender composition (2).

Across the included studies, sample characteristics were highly heterogeneous, spanning a broad spectrum of clinical and non-clinical populations. Psychiatric clinical samples constituted the largest group and included individuals with trauma-related disorders such as PTSD and cPTSD, often accompanied by depression, anxiety, emotion dysregulation, or suicidality (n=21) (162–166, 186, 264, 265, 267, 275, 277–280, 284, 286, 305, 316, 319, 320, 331). Another substantial subset comprised affective disorder cohorts, primarily major depressive disorder, frequently with comorbid anxiety, personality pathology, or trauma exposure (n=9) (13, 103, 282, 296, 299, 300, 306, 327, 341). In 38 studies depression was one of several Axis I diagnoses or high-prevalence comorbidity (13, 98, 99, 164, 165, 173, 174, 264, 265, 273, 275–278, 280–298, 324, 328, 331, 337, 341). In 31 studies other clinical populations, such as with PTSD, OCD, ED, anxiety, somatic, dissociative disorders, and with secondary or comorbid depressive symptoms were evaluated (96, 97, 101, 162, 163, 166, 176, 186, 232, 266–270, 272, 279, 314, 316, 317, 319, 322, 323, 325, 329, 332–336, 339, 342). Several (n=7) studies assessed non-clinical samples with self-reported depressive symptoms (12, 105, 183, 274, 302, 330, 340). Numerous studies focused on anxiety spectrum conditions, including panic disorder, agoraphobia, generalized anxiety disorder (GAD), and social anxiety, with many samples enriched for treatment resistance or high comorbidity (n=15) (176, 177, 270, 272, 273, 294, 295, 297, 301, 310, 312, 332, 335, 339, 342). Anxiety disorders and anxiety symptoms were represented more granularly across the included studies (2, 12, 13, 96–99, 101, 103, 105, 162–166, 173, 174, 176, 177, 183, 186, 232, 264–342). Cohorts diagnosed with primary anxiety disorders were present in 12 studies, divided into specific: panic disorder (with/without agoraphobia) (n=6) (176, 270, 272, 310, 335, 339), social anxiety disorder/generalized social phobia (n=2) (332, 342), GAD and mixed International Statistical Classification of Diseases and Related Health Problems, 10th Revision (ICD-10) anxiety disorders (n=4) (98, 99, 273, 337). Anxiety disorders, as a major outcome or covariate, were present alongside depressive disorders, PTSD, somatoform disorders, ED, personality and dissociative disorders in 39 studies (13, 98, 99, 164, 165, 173, 174, 264, 265, 273, 275–278, 280–298, 320, 324, 328, 331, 337, 341). Other clinical populations, such as those with PTSD/cPTSD, trauma, ED, dissociative disorders, somatic/FND, neurological, or medical conditions as the primary diagnosis with secondary or comorbid anxiety symptoms, were investigated in 42 studies (97, 101, 103, 105, 162, 163, 166, 183, 186, 232, 266–272, 274–276, 279, 299, 300, 303, 306, 314, 316, 317, 319, 321–323, 325, 329, 332–336, 339, 340, 342). Non-clinical/community samples with self-reported anxiety (no formal anxiety diagnosis required) were investigated in 10 studies (2, 12, 105, 183, 186, 274, 279, 302, 330, 340). A further group involved OCD, frequently pharmacoresistant and associated with elevated depressive, anxiety, and personality features (n=10) (96, 97, 271, 288, 307, 317, 325, 326, 333, 336). These anxiety-related categories were not mutually exclusive, as many studies examined anxiety either as a primary diagnosis, a comorbid condition, or a transdiagnostic outcome. Several studies examined EDs, including anorexia nervosa, bulimia nervosa, binge-eating disorder, and not otherwise specified, often addressing dissociation in relation to binge episodes, self-injury, or treatment response (n=6) (101, 304, 308, 322, 329, 334). A distinct subset comprised neurotic spectrum disorders and mixed anxiety–depressive presentations, characterized by pervasive comorbidity and chronic impairment (n=5) (98, 99, 290, 303, 337).

Across clinical and community samples, a group of studies focused on mixed dissociative disorder samples- populations in which dissociation was prominent or used as an inclusion criterion, but diagnostic presentations spanned numerous comorbid conditions, including affective, trauma-related, somatoform, and personality pathology. These heterogeneous dissociative samples highlight the transdiagnostic nature of dissociation and its frequent entanglement with complex clinical profiles (n=12) (173, 174, 277, 278, 280, 284, 289, 292, 293, 309, 316, 319). One study examined functional neurological disorder, also marked by frequent comorbid PTSD, depression, and anxiety, further illustrating the overlap between dissociative and neuropsychiatric phenomena (232).

Beyond psychiatry, several studies investigated non-psychiatric clinical groups, including patients with neurological or somatic conditions in which dissociation, trauma exposure, or psychopathology were evaluated as correlates or outcomes. These included migraine (268), fibromyalgia (285), epilepsy and FS (314), TIA (269), COVID-19 recovery without prior psychiatric history (266), and medically ill patients with comorbid depressive symptoms (291, 306).

Finally, a set of studies relied on non-clinical or community populations, 3 including healthy volunteers (283, 315, 318), 5 including university students (12, 105, 183, 330, 340), and 3 on general-population samples assessed for dissociative or trauma-related phenomena (174, 302, 328). These groups contributed important data on dissociation in normative or subclinical contexts and enabled comparisons with clinical presentations.

Collectively, these heterogeneous dissociative samples highlight the transdiagnostic nature of dissociation and explain why rigid diagnostic grouping would inadequately capture its clinical relevance.

#### 3.4.3 Assessment tools

Across mixed dissociation studies, assessment strategies were notably heterogeneous, reflecting substantial variability in how dissociation was conceptualized, operationalized, and positioned relative to depression and anxiety.

Dissociation was assessed using a wide range of self-report questionnaires, clinician-administered instruments, and structured diagnostic interviews, reflecting substantial methodological heterogeneity. The most frequently applied measure was the DES, including the original version, DES-II, and validated language adaptations, which was used across the majority of clinical and non-clinical samples (2, 12, 96–99, 101, 103, 163, 177, 183, 267, 268, 270–273, 276, 278, 282–285, 287–289, 292–295, 298–301, 303, 305, 307, 311, 313–317, 319, 320, 324–326, 328–331, 333, 335, 337–339, 341, 342). The DES-Taxon (DES-T) was employed to identify pathological or taxon-level dissociation in a smaller subset of studies, primarily those focusing on dissociative depression, highly dissociative samples, or panic and trauma-related disorders (173, 270, 272, 277, 279, 282, 339, 342). Several studies used alternative self-report dissociation measures, including the Dissociative Symptom Scale (DSS) (162, 275, 298), MDI (186, 232, 264), SDQ-20 and its abbreviated versions (13, 284, 288, 324, 337), and DIS-Q (297, 322, 332, 334). State or peritraumatic dissociation was specifically assessed using the Clinician-Administered Dissociative States Scale (CADSS) (274, 284, 308), the Peritraumatic Dissociative Experiences Questionnaire (PDEQ) (269, 302, 318, 340), and the Acute Dissociation Inventory (ADI-D) (315), highlighting process-oriented and experimentally informed approaches to dissociation. Diagnostic assessment of dissociative disorders relied on structured interviews, including DDIS (174, 284, 289, 293, 296, 319), the Self-Report Dissociative Disorders Interview Schedule (SR-DDIS) (13, 173, 277), and SCID-D/Structured Clinical Interview for DSM-IV Dissociative Disorders- Revised (SCID-D-R) (293, 316, 324). Less frequently used instruments included the Heidelberg Dissociation Inventory (HDI) (280), the MID (324), and FDS (328, 341).

Anxiety symptoms were assessed using a wide range of self-report questionnaires, clinician-rated scales, and disorder-specific severity measures. The most frequently employed instrument was the BAI, which was applied across heterogeneous clinical populations including panic disorder, obsessive–compulsive disorder, depressive disorders, neurotic spectrum disorders, somatic conditions, and mixed psychiatric samples (96–99, 163, 165, 268, 270–273, 285, 287, 288, 294, 300–303, 307, 312, 317, 325, 326, 333, 335, 337, 339, 342). Clinician-rated assessments of anxiety severity were commonly conducted using the HAM-A/HARS, particularly in mood, trauma-related, panic, somatoform, and mixed inpatient samples (96, 97, 99, 166, 287, 299, 312, 321, 331, 335, 338). Brief screening instruments were also widely used, including the Generalized Anxiety Disorder Scale (GAD-7) in outpatient, neurological, and functional disorder samples (164, 232, 265, 269), as well as HADS-A in medical, trauma-related, and ED populations (266, 275, 308, 323). Several studies relied on anxiety measures tailored to specific diagnostic contexts. These included the Liebowitz Social Anxiety Scale (LSAS) in social anxiety and neurotic spectrum disorder samples (98, 99, 332, 342), the Panic Disorder Severity Scale (PDSS) in panic disorder cohorts (176, 270, 272, 297), and additional panic- and agoraphobia-focused instruments such as the Panic and Agoraphobia Scale and Panic Attack Form (177, 294). Disorder-specific anxiety measures were also used in dental phobia (323) and ED populations (329). State-trait approaches to anxiety were implemented using the STAI in experimental, daily-diary, and laboratory-based studies (101, 274, 298, 315). Less frequently applied instruments included the Penn State Worry Questionnaire (325), Taylor Manifest Anxiety Scale (177), Zung Self-Rating Anxiety Scale (330), and anxiety-related scoring from the General Health Questionnaire (335). Anxiety was additionally indexed through broader psychopathology inventories, including SCL-90/SCL-90-R and Brief Symptom Inventory (BSI) (162, 176, 267, 288, 295, 311, 312, 314, 323, 324, 328, 338, 341), or inferred from structured diagnostic interviews without a dedicated severity scale (291, 327).

Depressive symptoms were assessed using a broad range of self-report questionnaires, clinician-rated scales, and diagnostic classifications. The most frequently employed instrument was the BDI, including both the original and BDI-II versions, which was applied across diverse clinical and non-clinical samples encompassing trauma-related disorders, affective disorders, anxiety disorders, EDs, somatic conditions, and community populations (96–99, 101, 103, 163–165, 177, 183, 267, 268, 270–273, 278, 282, 283, 285, 286, 288, 294, 298–303, 307, 311–313, 316, 320, 325, 326, 328–331, 335, 337–342). Clinician-rated measures of depressive severity were also commonly used, most notably the HDRS/HAM-D, particularly in trauma-focused, somatoform, panic disorder, and mood disorder studies (166, 286, 287, 299, 312, 321, 331, 335, 338). Several studies relied on brief screening instruments, including the Patient Health Questionnaire-9 (PHQ-9) (164, 173, 232, 265, 269, 275, 277–279) and the depression subscale of the HADS-D (266, 275, 308, 323), facilitating assessment in medical, neurological, and mixed psychiatric samples. Less frequently used measures included the Center for Epidemiological Studies Depression Scale (CES-D) (321), the Zung Self-Rating Depression Scale (330), and the depression subscale of the Depression Anxiety Stress Scale (DASS) (183, 264). In addition, depressive symptomatology was sometimes derived from broader psychopathology instruments, such as the Symptom Checklist-90 (SCL-90/SCL-90-R) or its Global Severity Index (162, 267, 288, 291, 295, 311, 312, 314, 325, 338, 341), or from diagnostic interviews and medical records without a dedicated severity scale (291, 296, 306, 327).

#### 3.4.4 Conceptual approaches to dissociation

Across the included studies, dissociation was conceptualized in several distinct yet complementary ways, reflecting its heterogeneous theoretical status across psychiatric and psychological research.

A first group of studies positioned dissociation as the primary clinical construct of interest, treating it as a central organizing dimension shaping clinical presentation, risk profiles, or treatment response, regardless of underlying diagnostic categories (173, 174, 277, 278, 280, 284, 287, 289, 292, 293, 296, 309, 316, 319). In these studies, dissociation was not merely a secondary symptom but the main explanatory variable, often examined in clinically complex and highly comorbid samples. Although dissociation constituted the primary analytic focus in these studies, they were not assigned to the DID/CDD, DPDR, or FND categories because they did not involve samples formally defined by a specific dissociative diagnosis or phenomenologically distinct dissociative subtype, but rather examined dissociation as a dominant clinical dimension within heterogeneous primary diagnostic groups.

A second group conceptualized dissociation primarily within a trauma-related or post-traumatic framework, examining it as a component, correlate, or mediator of post-traumatic stress reactions in populations exposed to interpersonal trauma, childhood abuse, discrimination-related trauma, or other adverse experiences (105, 162, 163, 165, 166, 183, 186, 264, 267, 275, 276, 279, 286, 302, 305, 320, 328, 330, 331). In these studies, dissociation was embedded within broader models of PTSD or complex trauma and was frequently linked to affective dysregulation, attachment insecurity, or suicidality.

In the largest subset of studies, dissociation was approached as a transdiagnostic symptom dimension, assessed dimensionally and examined in relation to symptom severity, personality traits, self-stigma, functional impairment, or treatment response across a wide range of psychiatric diagnoses, including depressive, anxiety, obsessive-compulsive, eating, and neurotic spectrum disorders (2, 13, 96–99, 101, 103, 164, 176, 177, 265, 270–273, 282, 288, 290, 294, 295, 297, 299–301, 303, 304, 307, 310–313, 317, 322–327, 329, 331–339, 341, 342). Within this framework, dissociation functioned as a marker of clinical complexity rather than a diagnosis-specific phenomenon.

A smaller group of studies emphasized dissociation as a state-dependent or process-related phenomenon, focusing on peritraumatic dissociation, experimentally induced dissociative states, or short-term fluctuations related to stress, sleep deprivation, or cognitive avoidance processes (274, 283, 298, 315, 318, 340). These approaches highlighted dissociation as a dynamic response rather than a stable trait.

Finally, several studies examined dissociation in somatic, neurological, or medically ill populations, or within consultation-liaison and neuropsychiatric contexts, conceptualizing dissociation as a psychological correlate of physical illness or neurological conditions rather than a primary psychiatric disorder (232, 266, 268, 269, 281, 285, 291, 306, 314, 321).

Together, these diverse conceptualizations underscore the transdiagnostic and context-sensitive nature of dissociation across clinical and non-clinical settings.

#### 3.4.5 Mixed dissociation in relation to anxiety and depression

Across studies examining mixed dissociative phenomena, dissociation consistently co-occurred with elevated depressive and anxiety symptoms across a wide range of clinical and non-clinical populations. In the majority of studies, higher dissociative symptom severity was moderately to strongly correlated with greater depressive and/or anxiety symptoms, regardless of the primary diagnosis or recruitment setting (13, 97–99, 101, 103, 105, 162, 163, 165, 166, 183, 232, 264–270, 272–275, 282, 285, 296, 299–301, 305, 311, 313, 314, 320, 321, 324, 327, 328, 330, 331, 333, 334, 341, 342). At the same time, the magnitude, direction, and clinical significance of these associations varied substantially across diagnostic and contextual frameworks.

In trauma-related and stress-linked conditions, dissociation showed particularly robust associations with both depression and anxiety and often acted as a mediator between trauma exposure and affective symptom severity (101, 105, 162, 163, 173, 183, 186, 264, 266, 269, 274, 275, 286, 305, 320, 328, 330). Several intervention studies further demonstrated that reductions in dissociation during trauma-focused treatments paralleled improvements in depression and anxiety, suggesting partially overlapping therapeutic mechanisms across these symptom domains (162, 163, 165, 166, 320, 331).

Findings were more heterogeneous within anxiety-spectrum disorders, particularly panic disorder and social phobia. Some studies reported moderate to strong associations between dissociation and anxiety severity, with higher dissociation associated with poorer treatment response or greater functional impairment (270, 272, 273, 294, 297, 301, 332, 342). In contrast, other investigations observed weak or absent associations between dissociation and depressive or anxiety symptom severity, indicating that panic-bound or situational dissociation may reflect a state-dependent phenomenon rather than a marker of generalized affective distress (176, 312, 335, 337). Experimental and process-oriented studies supported this distinction, showing that acute or trait dissociation could attenuate physiological anxiety responses without corresponding changes in mood (308, 315, 318).

Across mood disorder samples, dissociation was consistently linked to depressive severity, suicidality, and poorer clinical outcomes. Higher dissociative symptom levels were associated with greater hopelessness, increased suicide risk, and reduced treatment response among individuals with depressive symptoms (103, 299, 300, 311, 321, 327, 339). Importantly, several studies identified dissociation as independently associated with adverse outcomes even after controlling for depressive and anxiety symptom severity, underscoring its clinical relevance beyond global affective burden (103, 311, 327, 333). Related conceptualizations of “dissociative depression” further suggested that depressive presentations marked by emotional numbing, detachment, and identity disturbance may constitute a distinct clinical subtype with specific prognostic and therapeutic implications (12, 13, 173, 296).

Taken together, the mixed dissociation literature suggests that dissociation functions not merely as a correlate of depressive and anxiety symptom severity, but as a transdiagnostic modifier of clinical course, treatment response, and prognostic trajectories across diagnostic categories. These findings support the routine assessment of dissociative symptoms in patients presenting with depression and anxiety and suggest that targeted interventions addressing dissociation may be necessary to optimize affective outcomes, particularly in trauma-exposed and treatment-resistant populations.

## 4 Discussion

**Figure 3:**
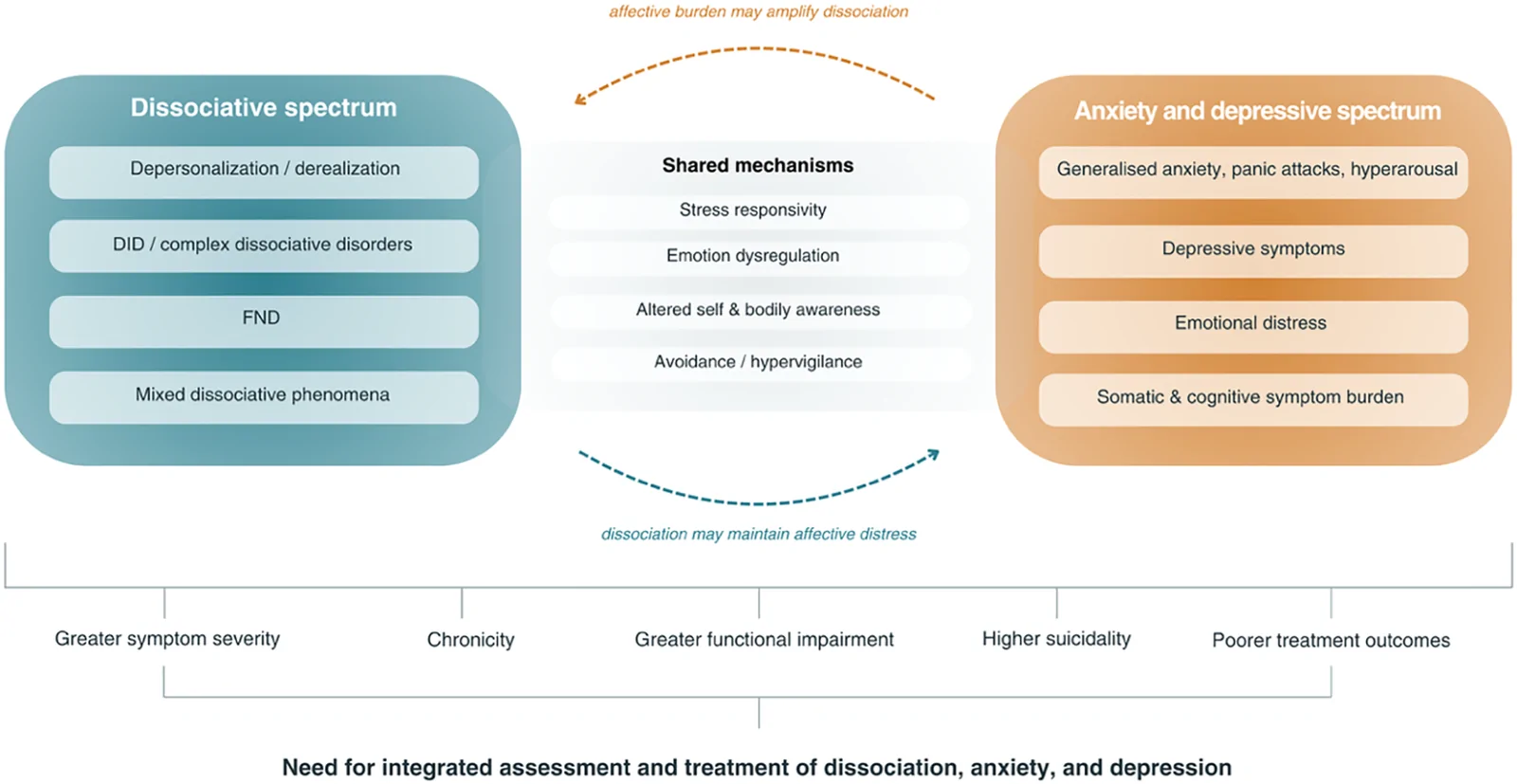
Hypothesized reciprocal pattern of co-occurrence between dissociation and anxiety and/or depression, generated by this scoping review and proposed for testing in future longitudinal research.

Across dissociative subtypes, marked differences emerged in the prevalence and severity of comorbid anxiety and depression. The highest affective burden was observed in DID and CDD, where lifetime major depression was reported in roughly two-thirds of patients across cohorts and anxiety disorders affected approximately half to three-quarters of patients, often with severe, chronic, and trauma-related presentations. FND showed intermediate but substantial affective comorbidity, with clinically significant anxiety and depressive symptoms present in roughly one-third to two-thirds of patients, particularly pronounced in dissociative seizures. In contrast, DPDR was characterized by very high rates of anxiety-especially panic disorder- while depressive symptoms were frequent but typically of mild to moderate severity. Conversely, among patients with anxiety and/or depressive disorders, dissociative symptoms were common and dimensionally distributed, with severity increasing in the presence of affective comorbidity, chronicity, and trauma exposure. Depersonalization–derealization predominated in anxiety disorders, whereas broader psychoform and somatoform dissociation was more characteristic of chronic depression and anxiety-depression comorbidity. Together, these findings indicate a graded pattern of co-occurrence in which greater dissociative complexity is associated with more severe and persistent affective psychopathology, supporting transdiagnostic models that position dissociation as a marker of clinical severity and mechanism of symptom persistence rather than a diagnosis-specific phenomenon (**Figure 3**).

**Figure 4:**
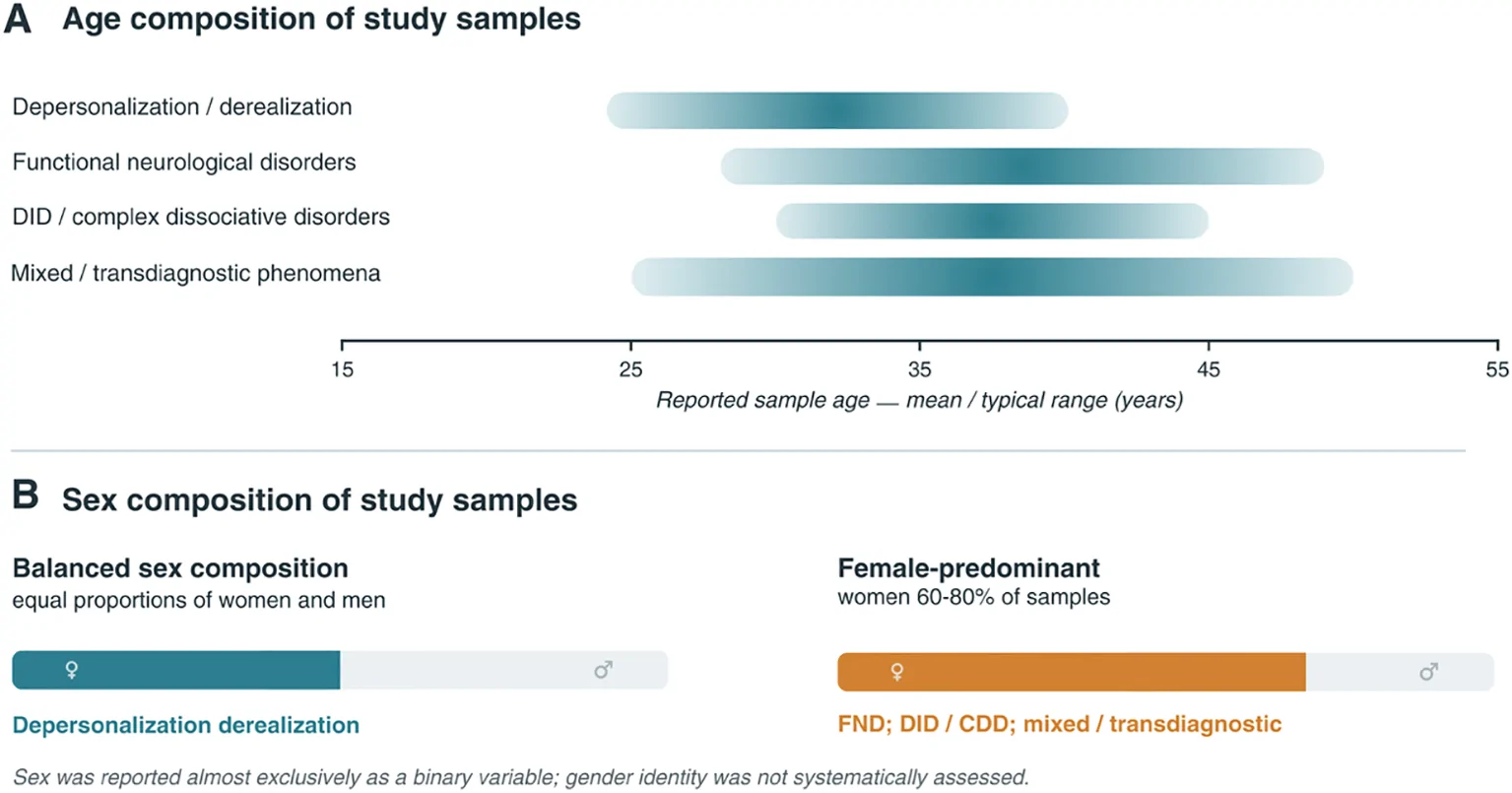
Age and sex composition of the included study samples. **(A)** reported sample age (mean/typical range) by dissociative category. **(B)** sex composition. Sex was reported almost exclusively as a binary variable; gender identity was not systematically assessed.

Across the reviewed literature, dissociative phenomena in anxiety and depressive disorders showed consistent age- and sex-related patterns (**Figure 4**). Depersonalization–derealization and functional neurological symptoms typically emerged in adolescence or early adulthood, with peak onset in the late teens to twenties, whereas depression and anxiety displayed a broader age distribution, with substantial clinical burden extending into mid- and late adulthood (5, 6, 28, 170). Sex differences varied by dissociative subtype: depersonalization-derealization disorder showed relatively balanced sex distributions (25, 26, 28, 149), while functional neurological disorder and dissociative seizures demonstrated a clear female predominance, closely mirroring patterns observed in anxiety and depressive disorders (5, 33, 35, 37). Across studies, sex was predominantly operationalized in binary terms, and gender identity was rarely assessed, limiting conclusions regarding gender-related vulnerability or resilience (1, 20). Overall, these findings support a developmental and transdiagnostic interpretation, in which dissociation may represent an early-emerging stress-related process that interacts over time with affective psychopathology and is differentially shaped by sex-related risk factors.

In the reviewed literature, dissociative phenomena in individuals with anxiety and/or depression were associated with partially overlapping but distinct risk factor profiles, varying by dissociative subtype. Depersonalization and derealization were most strongly linked to panic symptoms, anxiety sensitivity, heightened interoceptive focus, and acute or chronic stress, often emerging in the context of hyperarousal and threat-related processing. In contrast, broader psychoform dissociation was more frequently associated with chronic or recurrent depression, emotional numbing, alexithymia, and cumulative trauma exposure, particularly in individuals with early-life adversity or prolonged affective illness (343). Somatoform dissociation and functional neurological symptoms were linked to a combination of affective dysregulation, trauma-related psychopathology, maladaptive illness beliefs, and persistent psychosocial stress, with symptom chronicity and comorbid anxiety–depression amplifying risk (344). Across dissociative types, greater dissociation was consistently associated with higher affective severity, comorbidity, and functional impairment, supporting a dimensional model in which dissociation reflects the interaction between affective vulnerability, stress exposure, and difficulties in integrating emotional and bodily experience.

Across the reviewed literature, dissociation and affective symptoms were consistently associated, with important implications for clinical course and prognosis (**Figure 3**). On one hand, the presence and complexity of dissociation influenced affective outcomes: depersonalization-derealization was associated with persistent anxiety and reduced quality of life (345), while broader psychoform and somatoform dissociation, including FND, was associated with greater chronicity, functional impairment, and poorer treatment response in both depression and anxiety (346). On the other hand, comorbid anxiety and depressive symptoms substantially shaped dissociative outcomes, with higher affective burden linked to symptom persistence, relapse, treatment resistance, and increased disability across dissociative subtypes. This pattern was particularly pronounced in complex or trauma-associated dissociative presentations, where affective comorbidity was associated with more frequent relapses and elevated suicidality (4). Taken together, these associations suggest that dissociation and affective psychopathology may form a self-perpetuating clinical pattern, a hypothesis that warrants confirmation in longitudinal studies. They also underscore the need for integrated assessment and treatment strategies that address both domains concurrently (**Figure 3**).

In the reviewed studies, dissociation, depression, and anxiety were assessed using a heterogeneous set of self-report questionnaires and clinician-rated instruments, reflecting differences in conceptual focus and study design. Dissociation was most commonly measured with broad screening tools such as the DES and its variants, alongside disorder-specific measures for depersonalization–derealization, while somatoform and functional dissociation were often inferred from symptom profiles or neurological assessments rather than captured with dedicated scales. Depression and anxiety were typically assessed using standardized affective symptom measures (e.g. BDI, Hamilton Scales, HADS), enabling cross-study comparison of affective severity. However, the frequent use of self-report instruments and variability in cut-offs limited comparability across studies and constrained conclusions regarding diagnostic thresholds. Overall, the literature highlights a need for integrated and harmonized assessment approaches that jointly capture dissociative and affective dimensions to better characterize their interaction and prognostic significance.

Treatment approaches for dissociation in patients with anxiety and depression, and for anxiety and depression in patients with dissociative presentations, largely overlapped and reflected a integrated treatment strategy. Cognitive–behavioural interventions targeting anxiety, panic, and maladaptive attentional focus were most consistently associated with improvement in depersonalization–derealization, particularly when anxiety was the dominant driver of dissociative symptoms (347). In contrast, broader psychoform and somatoform dissociation, including FND, often required multimodal interventions combining psychotherapy, psychoeducation, and rehabilitation, with outcomes strongly influenced by concurrent treatment of depressive and anxiety symptoms. Pharmacological treatment was primarily directed at comorbid anxiety and depression rather than dissociation per se, with symptom improvement in dissociation appearing secondary to affective stabilization. Across dissociative subtypes, untreated or persistent affective symptoms were associated with poorer dissociative outcomes, while reductions in anxiety and depression were linked to improved functioning and symptom remission, underscoring the need for coordinated treatment addressing both domains simultaneously.

**Figure 5:**
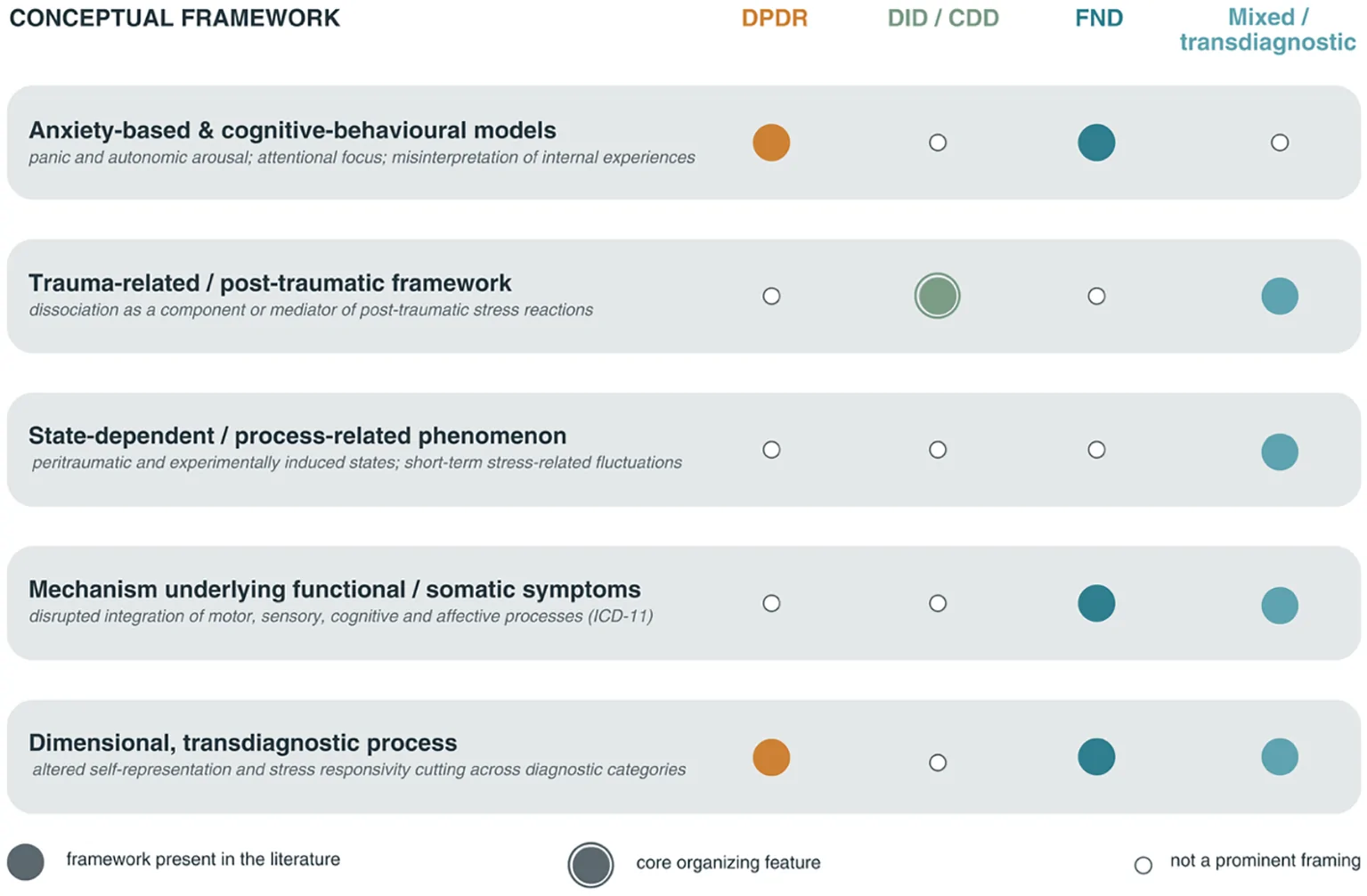
Conceptual approaches to dissociation across the included studies, mapped by dissociative category.

**Figure 6:**
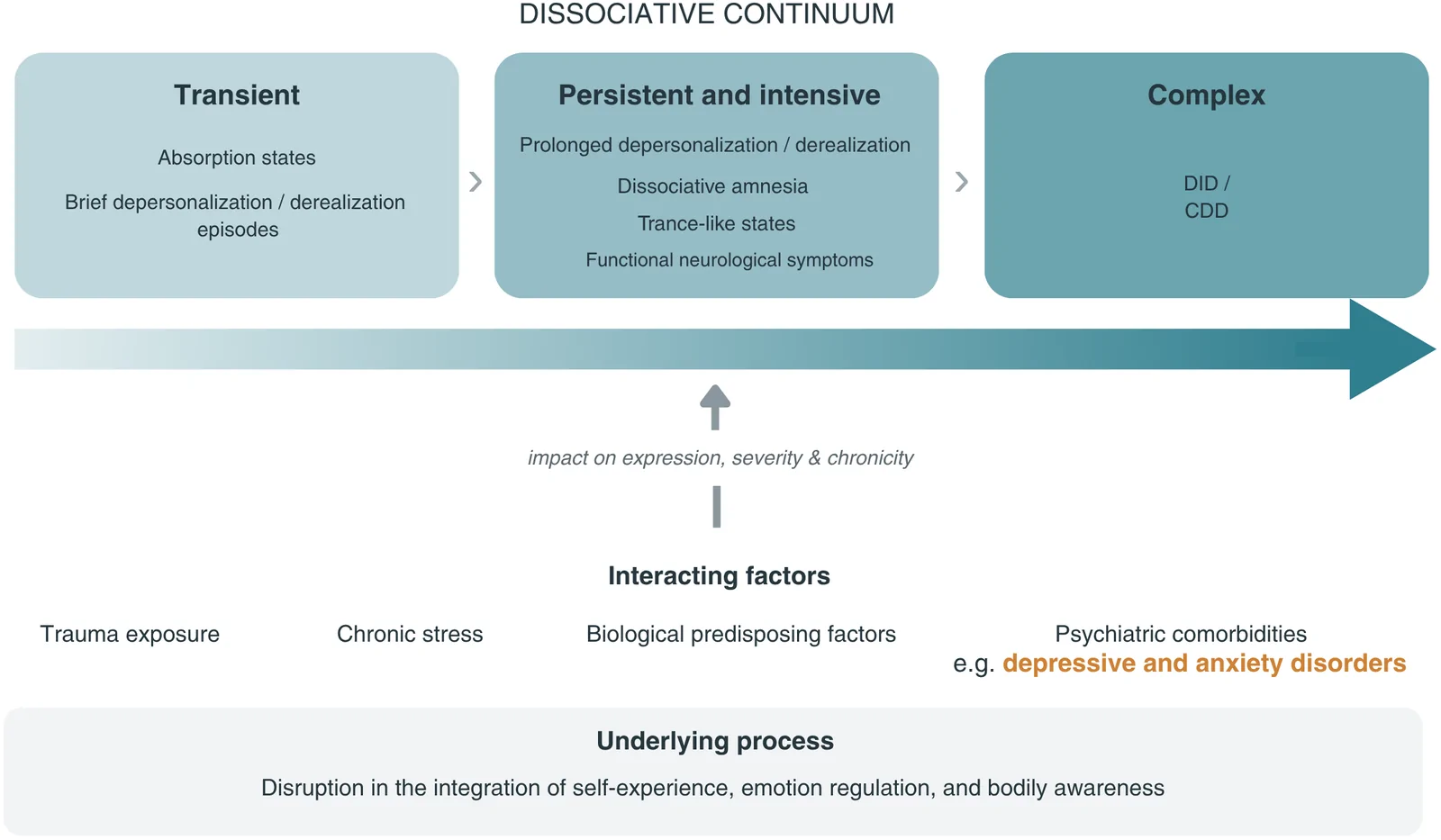
Transdiagnostic dimensional framework: the dissociative continuum and interacting factors shaping dissociative expression, severity, and chronicity.

The included studies framed dissociation in several distinct ways: as an anxiety-related and cognitive-behavioural phenomenon, a post-traumatic process, a state-dependent reaction, a mechanism underlying functional symptoms, or a dimensional transdiagnostic construct, with the dominant framing differing markedly across dissociative categories (**Figure 5**). Taken together, the reviewed evidence supports a transdiagnostic and dimensional conceptual framework for dissociation, in which dissociative phenomena reflect stress-related disruptions in the integration of self-experience, emotion regulation, and bodily awareness. Within this framework, dissociation spans a continuum from transient depersonalization and derealization commonly observed in anxiety and depressive disorders, to more persistent and complex dissociative presentations, including FND as classified in ICD-11 (**Figure 6**). Affective symptoms, trauma exposure, and chronic stress emerge as key interacting factors that modulate dissociative expression, severity, and chronicity (**Figure 6**). This model moves beyond categorical distinctions and emphasizes dissociation as a dynamic process linking affective psychopathology with functional neurological manifestations across the lifespan (**Figure 6**).

Several limitations should be considered when interpreting the findings of this scoping review. First, the included studies were highly heterogeneous in design, populations, and outcome measures, limiting direct comparison and precluding quantitative synthesis. Second, dissociation, anxiety, and depression were predominantly assessed using self-report instruments, often with varying cut-offs and conceptual definitions, which may have led to misclassification and inflated prevalence estimates. Third, most studies were cross-sectional, restricting conclusions about temporal or causal relationships between dissociation and affective symptoms. Fourth, sex was usually reported in binary terms and gender identity was rarely assessed, limiting insights into gender-related vulnerability. Fifth, studies of FND accounted for the largest share of the included literature; cross-category claims may therefore be disproportionately influenced by findings from this category, and conclusions drawn across dissociative categories should be interpreted with this imbalance in mind. Sixth, the literature search was completed in January 2025; studies published thereafter are not represented in this review, and given the pace of research in this field, more recent evidence may refine some of the patterns described here. Finally, the literature was dominated by clinical and Western samples, raising concerns about selection bias and generalizability to community or non-Western populations. These limitations highlight the need for longitudinal, methodologically harmonized, and more inclusive research designs.

## 5 Conclusions

This scoping review synthesizes a broad and heterogeneous body of literature to clarify the prevalence, conceptualization, and clinical significance of dissociation across anxiety and depressive disorders, including depersonalization-derealization and functional neurological presentations as classified in ICD-11. Across diagnostic categories, dissociation emerged as a common, dimensional, and clinically meaningful phenomenon associated with greater affective severity, functional impairment, and poorer prognosis. The review highlights consistent age and sex-related patterns, substantial co-occurrence between dissociation and affective symptoms, and considerable methodological variability in assessment and study design. Together, these findings support transdiagnostic and integrative models in which dissociation represents a key process linking affective psychopathology with functional and somatic symptom expression.

Future research should prioritize longitudinal and mechanistic studies to clarify the temporal relationships between dissociation, anxiety, and depression and to identify causal pathways. Greater methodological harmonization is needed, including the use of integrated assessment tools that simultaneously capture dissociative and affective dimensions across diagnostic categories. Studies should also adopt developmentally informed and sex- and gender-inclusive designs, and extend beyond Western clinical samples to improve generalizability. Finally, randomized trials examining integrated, mechanism-based interventions are needed to determine how targeting dissociation alongside anxiety and depression influences long-term clinical outcomes.

## Data Availability

The original contributions presented in the study are included in the article/**Supplementary Material**. The review protocol is publicly available on the Open Science Framework (https://osf.io/hnjpb/). Further inquiries can be directed to the corresponding author.

## References

[1] American Psychiatric Association . Diagnostic and Statistical Manual of Mental Disorders : DSM-5: Fifth Edition. Arlington, VA: American Psychiatric Association (2013).

[2] Lyssenko L Schmahl C Bockhacker L Vonderlin R Bohus M Kleindienst N . Dissociation in psychiatric disorders: A meta-analysis of studies using the dissociative experiences scale. Am J Psychiatry. (2018) 175:37–46. doi: 10.1176/appi.ajp.2017.17010025 28946763

[3] Maaranen P Tanskanen A Honkalampi K Haatainen K Hintikka J Viinamaki H . Factors associated with pathological dissociation in the general population. Aust N Z J Psychiatry. (2005) 39:387–94. doi: 10.1080/j.1440-1614.2005.01586.x 15860027

[4] Boyer SM Caplan JE Edwards LK . Trauma-related dissociation and the dissociative disorders:: Neglected symptoms with severe public health consequences. Dela J Public Health. (2022) 8:78–84. doi: 10.4324/9781003666424-29 PMC9162402 35692991

[5] Javaid SF Hashim IJ Hashim MJ Stip E Samad MA Ahbabi AA . Epidemiology of anxiety disorders: Global burden and sociodemographic associations. Middle East Curr Psychiatry. (2023) 30:44. doi: 10.1186/s43045-023-00315-3 38164791

[6] GBD 2019 Mental Disorders Collaborators . Global, regional, and national burden of 12 mental disorders in 204 countries and territories, 1990-2019: A systematic analysis for the global burden of disease study 2019. Lancet Psychiatry. (2022) 9:137–50. doi: 10.1016/s2215-0366(21)00395-3 35026139 PMC8776563

[7] Choi KW Kim YK Jeon HJ . Comorbid anxiety and depression: Clinical and conceptual consideration and transdiagnostic treatment. Adv Exp Med Biol. (2020) 1191:219–35. doi: 10.1007/978-981-32-9705-0_14 32002932

[8] Zhou J Zhou J Feng L Feng Y Xiao L Chen X . The associations between depressive symptoms, functional impairment, and quality of life, in patients with major depression: Undirected and bayesian network analyses. Psychol Med. (2023) 53:6446–58. doi: 10.1017/s0033291722003385 36349712 PMC10600944

[9] Rapaport MH Clary C Fayyad R Endicott J . Quality-of-life impairment in depressive and anxiety disorders. Am J Psychiatry. (2005) 162:1171–8. doi: 10.1176/appi.ajp.162.6.1171 15930066

[10] Prasko J Grambal A Kasalova P Kamardova D Ociskova M Holubova M . Impact of dissociation on treatment of depressive and anxiety spectrum disorders with and without personality disorders. Neuropsychiatr Dis Treat. (2016) 12:2659–76. doi: 10.2147/ndt.s118058 27799774 PMC5074730

[11] Warshaw MG Fierman E Pratt L Hunt M Yonkers KA Massion AO . Quality of life and dissociation in anxiety disorder patients with histories of trauma or PTSD. Am J Psychiatry. (1993) 150:1512–6. doi: 10.1176/ajp.150.10.1512 8379556

[12] Sar VM Turk-Kurtca TP . The vicious cycle of traumatic narcissism and dissociative depression among young adults: A trans-diagnostic approach. J Trauma Dissociation. (2021) 22:502–21. doi: 10.1080/15299732.2020.1869644 33427111

[13] Fung HW Chan C . A preliminary study of the clinical differences between dissociative and nondissociative depression in Hong Kong: Implications for mental health practice. Soc Work Health Care. (2019) 58:564–78. doi: 10.1080/00981389.2019.1597006 30958123

[14] Powers A Cross D Fani N Bradley B . PTSD, emotion dysregulation, and dissociative symptoms in a highly traumatized sample. J Psychiatr Res. (2015) 61:174–9. doi: 10.1016/j.jpsychires.2014.12.011 25573648 PMC4308496

[15] Cernis E Evans R Ehlers A Freeman D . Dissociation in relation to other mental health conditions: An exploration using network analysis. J Psychiatr Res. (2021) 136:460–7. doi: 10.1016/j.jpsychires.2020.08.023 PMC8039185 33092867

[16] Spiegel D Lewis-Fernandez R Lanius R Vermetten E Simeon D Friedman M . Dissociative disorders in DSM-5. Annu Rev Clin Psychol. (2013) 9:299–326. doi: 10.1146/annurev-clinpsy-050212-185531 23394228

[17] Lynn SJ Lilienfeld SO Merckelbach H Giesbrecht T van der Kloet D . Dissociation and dissociative disorders: Challenging conventional wisdom. Curr Dir psychol Sci. (2012) 21:48–53. doi: 10.1177/0963721411429457

[18] Tricco AC Lillie E Zarin W O'Brien KK Colquhoun H Levac D . PRISMA extension for scoping reviews (PRISMA-ScR): Checklist and explanation. Ann Intern Med. (2018) 169:467–73. doi: 10.7326/m18-0850 30178033

[19] Ouzzani M Hammady H Fedorowicz Z Elmagarmid A . Rayyan-a web and mobile app for systematic reviews. Syst Rev. (2016) 5:210. doi: 10.1186/s13643-016-0384-4 27919275 PMC5139140

[20] Organization WH . International statistical classification of diseases and related health problems (10th revision). (2016).

[21] Organization WH . International classification of diseases, eleventh revision (ICD-11). (2022).

[22] Edwards MJ Adams RA Brown H Pareés I Friston KJ . A bayesian account of 'hysteria'. Brain. (2012) 135:3495–512. doi: 10.1093/brain/aws129 22641838 PMC3501967

[23] Brown RJ . Dissociation and functional neurologic disorders. Handb Clin Neurol. (2016) 139:85–94. doi: 10.1016/B978-0-12-801772-2.00008-4 27719880

[24] Page MJ McKenzie JE Bossuyt PM Boutron I Hoffmann TC Mulrow CD . The PRISMA 2020 statement: An updated guideline for reporting systematic reviews. Bmj. (2021) 372:n71. doi: 10.31222/osf.io/v7gm2_v1 33782057 PMC8005924

[25] Baker D Hunter E Lawrence E Medford N Patel M Senior C . Depersonalisation disorder: Clinical features of 204 cases. Br J Psychiatry. (2003) 182:428–33. doi: 10.1192/bjp182.5.428 12724246

[26] Michal M Adler J Wiltink J Reiner I Tschan R Wölfling K . A case series of 223 patients with depersonalization-derealization syndrome. BMC Psychiatry. (2016) 16. doi: 10.1186/s12888-016-0908-4 27349226 PMC4924239

[27] Van Dyck R Spinhoven P . Depersonalization and derealization during panic and hypnosis in low and highly hypnotizable agoraphobics. Int J Clin Exp Hypn. (1997) 45:41–54. doi: 10.1080/00207149708416105 8991295

[28] Hunter EC Sierra M David AS . The epidemiology of depersonalisation and derealisation. A systematic review. Soc Psychiatry Psychiatr Epidemiol. (2004) 39:9–18. doi: 10.1007/s00127-004-0701-4 15022041

[29] Schweden TLK Wolfradt U Jahnke S Hoyer J . Depersonalization under academic stress: Frequency, predictors, and consequences. Psychopathology. (2018) 51:252–61. doi: 10.1159/000489468 29945133

[30] Rodewald F Wilhelm-Gößling C Emrich HM Reddemann L Gast U . Axis-I comorbidity in female patients with dissociative identity disorder and dissociative disorder not otherwise specified. J Nervous Ment Dis. (2011) 199:122–31. doi: 10.1097/nmd.0b013e318208314e 21278542

[31] Atilan Fedai Ü Asoğlu M . Analysis of demographic and clinical characteristics of patients with dissociative identity disorder. Neuropsychiatr Dis Treat. (2022) 18:3035–44. doi: 10.2147/ndt.s386648 36597464 PMC9805736

[32] Ross CA Norton GR Wozney K . Multiple personality disorder: an analysis of 236 cases. Can J Psychiatry. (1989) 34:413–8. doi: 10.1037/e609942012-007 2766193

[33] Asadi-Pooya AA Farazdaghi M Asadi-Pooya H Fazelian K . Depression, anxiety, stress, and suicide risk in patients with functional seizures vs. those with epilepsy. Acta Neurol Belg. (2024) 124:169–73. doi: 10.1007/s13760-023-02365-0 37642895

[34] Nawfal O Nasreddine W Hmaimess G Dassouki M Beydoun A Toufaili H . Depression and anxiety in patients from Lebanon with new onset functional seizures. Seizure. (2021) 88:22–8. doi: 10.1016/j.seizure.2021.03.014 33799136

[35] Capitaine P Thomas B Gradel A Ferté T Branchard O Frison E . Evaluation of quality of life's prognostic factors in people with functional seizures. Rev Neurol (Paris). (2024) 180:524–31. doi: 10.1016/j.neurol.2023.09.007 38040548

[36] Walsh S Levita L Reuber M . Comorbid depression and associated factors in PNES versus epilepsy: Systematic review and meta-analysis. Seizure. (2018) 60:44–56. doi: 10.1016/j.seizure.2018.05.014 29906707

[37] Myers L Trobliger R Bonafina M Vazquez-Casals G Lancman M Lancman M . Depression, anxiety, and clinical history in Spanish-speaking American patients with psychogenic nonepileptic seizures (PNES) compared with Spanish-speaking American patients with epilepsy. Epilepsy Behav. (2020) 102:106694. doi: 10.1016/j.yebeh.2019.106694 31760198

[38] Martino I Bruni A Labate A Vasta R Cerasa A Borzì G . Psychopathological constellation in patients with PNES: A new hypothesis. Epilepsy Behav. (2018) 78:297–301. doi: 10.1016/j.yebeh.2017.09.025 29092782

[39] Maurer CW Liu VD LaFaver K Ameli R Wu T Toledo R . Impaired resting vagal tone in patients with functional movement disorders. Parkinsonism Relat Disord. (2016) 30:18–22. doi: 10.1016/j.parkreldis.2016.06.009 27334304 PMC5007207

[40] Nisticò V Ilia N Conte F Broglia G Sanguineti C Lombardi F . Forearm bisection task suggests an alteration in body schema in patients with functional movement disorders (motor conversion disorders). J Psychosom Res. (2024) 178:111610. doi: 10.1016/j.jpsychores.2024.111610 38359638

[41] Macchi ZA Kletenik I Olvera C Holden SK . Psychiatric comorbidities in functional movement disorders: a retrospective cohort study. Mov Disord Clin Pract. (2021) 8:725–32. doi: 10.1002/mdc3.13226 34307745 PMC8287186

[42] Demartini B Nisticò V Benayoun C Cigognini AC Ferrucci R Vezzoli A . Glutamatergic dysfunction, neuroplasticity, and redox status in the peripheral blood of patients with motor conversion disorders (functional movement disorders): a first step towards potential biomarkers discovery. Transl Psychiatry. (2023) 13:212. doi: 10.1038/s41398-023-02500-8 37330537 PMC10276860

[43] Ricciardi L Nisticò V Andrenelli E Cunha JM Demartini B Kirsch LP . Exploring three levels of interoception in people with functional motor disorders. Parkinsonism Relat Disord. (2021) 86:15–8. doi: 10.1016/j.parkreldis.2021.03.029 33819899

[44] Marotta A Fiorio M Riello M Demartini B Tecilla G Dallocchio C . Attentional avoidance of emotions in functional movement disorders. J Psychosom Res. (2020) 133:110100. doi: 10.1016/j.jpsychores.2020.110100 32224346

[45] Hebert C Behel JM Pal G Kasi R Kompoliti K . Multidisciplinary inpatient rehabilitation for functional movement disorders: a prospective study with long term follow up. Parkinsonism Relat Disord. (2021) 82:50–5. doi: 10.1016/j.parkreldis.2020.11.018 33248393

[46] Hubschmid M Aybek S Maccaferri GE Chocron O Gholamrezaee MM Rossetti AO . Efficacy of brief interdisciplinary psychotherapeutic intervention for motor conversion disorder and nonepileptic attacks. Gen Hosp Psychiatry. (2015) 37:448–55. doi: 10.1016/j.genhosppsych.2015.05.007 26099544

[47] Voon V Lang AE . Antidepressant treatment outcomes of psychogenic movement disorder. J Clin Psychiatry. (2005) 66:1529–34. doi: 10.4088/jcp.v66n1206 16401153

[48] Demartini B Ferrucci R Goeta D Ruggiero F D'Agostino A Priori A . The truth about cognitive impairment in functional motor symptoms: An experimental deception study with the guilty knowledge task. J Clin Neurosci. (2019) 64:174–9. doi: 10.1016/j.jocn.2019.03.005 30940452

[49] Taib S Ory-Magne F Brefel-Courbon C Moreau Y Thalamas C Arbus C . Repetitive transcranial magnetic stimulation for functional tremor: A randomized, double-blind, controlled study. Mov Disord. (2019) 34:1210–9. doi: 10.1002/mds.27727 31180620

[50] Lidstone SC Costa-Parke M Robinson EJ Ercoli T Stone J . Functional movement disorder gender, age and phenotype study: A systematic review and individual patient meta-analysis of 4905 cases. J Neurol Neurosurg Psychiatry. (2022) 93:609–16. doi: 10.1136/jnnp-2021-328462 35217516

[51] Gilmour GS Langer LK Lang AE MacGillivray L Lidstone SC . Neuropsychiatric phenotypes in functional movement disorder. CNS Spectr. (2023) 28:747–55. doi: 10.1017/s1092852923002353 37424291

[52] Serranová T Slovák M Kemlink D Šonka K Hallett M Růžička E . Prevalence of restless legs syndrome in functional movement disorders: A case-control study from the Czech Republic. BMJ Open. (2019) 9:e024236. doi: 10.3233/978-1-60750-096-4-23 PMC6347872 30670516

[53] Gilmour GS Langer LK Bhatt H MacGillivray L Lidstone SC . Factors influencing triage to rehabilitation in functional movement disorder. Mov Disord Clin Pract. (2024) 11:515–25. doi: 10.1002/mdc3.14007 38385766 PMC11078488

[54] Garcin B Villain N Mesrati F Naccache L Roze E Degos B . A single-center series of 482 patients with functional motor disorders. J Psychosom Res. (2021) 148:110565. doi: 10.1016/j.jpsychores.2021.110565 34252796

[55] Wang JT Cheng OM Xu G Xu SL Liu XD Ma S . Characteristics and prognosis of functional movement disorders in China: FMD-China registry study. Eur J Neurol. (2025) 32:e70372. doi: 10.1111/ene.70372 41074481 PMC12514011

[56] Perez DL Dworetzky BA Dickerson BC Leung L Cohn R Baslet G . An integrative neurocircuit perspective on psychogenic nonepileptic seizures and functional movement disorders: Neural functional unawareness. Clin EEG Neurosci. (2015) 46:4–15. doi: 10.1177/1550059414564521 25432161 PMC4363170

[57] Gelauff JM Rosmalen JGM Gardien J Stone J Tijssen MAJ . Shared demographics and comorbidities in different functional motor disorders. Parkinsonism Relat Disord. (2020) 70:1–6. doi: 10.1016/j.parkreldis.2019.11.018 31785442

[58] Binzer M Andersen PM Kullgren G . Clinical characteristics of patients with motor disability due to conversion disorder: A prospective control group study. J Neurol Neurosurg Psychiatry. (1997) 63:83–8. doi: 10.1136/jnnp.63.1.83 9221972 PMC2169635

[59] Defazio G Pastore A Pellicciari R Pierri G Gigante AF Fabio G . Personality disorders and somatization in functional and organic movement disorders. Psychiatry Res. (2017) 257:227–9. doi: 10.1016/j.psychres.2017.07.068 28780279

[60] Luca A Lo Castro T Mostile G Donzuso G Cicero CE Nicoletti A . Personality and psychopathological characteristics in functional movement disorders. PloS One. (2024) 19:e0303379. doi: 10.1371/journal.pone.0303379 38728293 PMC11086865

[61] Ercoli T Defazio G Geroin C Marcuzzo E Fabbrini G Bono F . Sudden onset, fixed dystonia and acute peripheral trauma as diagnostic clues for functional dystonia. Mov Disord Clin Pract. (2021) 8:1107–11. doi: 10.1002/mdc3.13322 34631946 PMC8485608

[62] Baizabal-Carvallo JF Alonso-Juarez M Jankovic J . Functional gait disorders, clinical phenomenology, and classification. Neurol Sci. (2020) 41:911–5. doi: 10.1007/s10072-019-04185-8 31832998

[63] Chouksey A Pandey S . Functional movement disorders in elderly. Tremor Other Hyperkinet Mov (N Y). (2019) 9. doi: 10.5334/tohm.463 PMC6691911 31413900

[64] Tinazzi M Geroin C Marcuzzo E Cuoco S Ceravolo R Mazzucchi S . Functional motor phenotypes: To lump or to split? J Neurol. (2021) 268:4737–43. doi: 10.1007/s00415-021-10583-w 33961091 PMC8563631

[65] Geroin C Petracca M Di Tella S Marcuzzo E Erro R Cuoco S . Elderly onset of functional motor disorders: Clinical correlates from the Italian registry. Mov Disord Clin Pract. (2024) 11:38–44. doi: 10.1002/mdc3.13916 38291844 PMC10828615

[66] Ercoli T Tinazzi M Geroin C Marcuzzo E Erro R Cuoco S . Do demographic and clinical features and comorbidities affect the risk of spread to an additional body site in functional motor disorders? J Neural Transm (Vienna). (2022) 129:1271–6. doi: 10.1007/s00702-022-02537-x 35972697 PMC9468120

[67] Batla A Stamelou M Edwards MJ Pareés I Saifee TA Fox Z . Functional movement disorders are not uncommon in the elderly. Mov Disord. (2013) 28:540–3. doi: 10.1002/mds.25350 23418043

[68] Schrag A Trimble M Quinn N Bhatia K . The syndrome of fixed dystonia: An evaluation of 103 patients. Brain. (2004) 127:2360–72. doi: 10.1093/brain/awh262 15342362

[69] Ertan S Uluduz D Ozekmekçi S Kiziltan G Ertan T Yalçinkaya C . Clinical characteristics of 49 patients with psychogenic movement disorders in a tertiary clinic in Turkey. Mov Disord. (2009) 24:759–62. doi: 10.1002/mds.22114 19205065

[70] Baik JS Lang AE . Gait abnormalities in psychogenic movement disorders. Mov Disord. (2007) 22:395–9. doi: 10.1002/mds.21283 17216648

[71] Issak S Williams G Kanaan RA Fini NA Nielsen G . Self-reported motor and non-motor symptoms in people with functional gait disorder: A cross-sectional study. Brain Behav. (2025) 15:e70208. doi: 10.1002/brb3.70208 39915104 PMC11802242

[72] Sandri A Bonetto C Fiorio M Salaorni F Bonardi G Geroin C . Unraveling the mechanisms of high-level gait control in functional gait disorders. J Neural Transm (Vienna). (2025) 132:95–104. doi: 10.1007/s00702-024-02829-4 39237791 PMC11735589

[73] Owczarek K . Psychological profile of patients with psychogenic pseudoepileptic seizures. Neurol Neurochir Pol. (2000) 34:1155–63. 11317492

[74] Sobregrau P Baillès E Carreño M Donaire A Boget T Setoain X . Psychiatric and psychological assessment of Spanish patients with drug-resistant epilepsy and psychogenic nonepileptic seizures (PNES) with no response to previous treatments. Epilepsy Behav. (2023) 145:109329. doi: 10.1016/j.yebeh.2023.109329 37453292

[75] Cohen ML Testa SM Pritchard JM Zhu J Hopp JL . Overlap between dissociation and other psychological characteristics in patients with psychogenic nonepileptic seizures. Epilepsy Behav. (2014) 34:47–9. doi: 10.1016/j.yebeh.2014.03.001 24681385

[76] Hendrickson R Popescu A Ghearing G Bagic A . Thoughts, emotions, and dissociative features differentiate patients with epilepsy from patients with psychogenic nonepileptic spells (PNESs). Epilepsy Behav. (2015) 51:158–62. doi: 10.1016/j.yebeh.2015.07.016 26283304

[77] Mazza M Della Marca G Martini A Scoppetta M Vollono C Valenti MA . Non-epileptic seizures (NES) are predicted by depressive and dissociative symptoms. Epilepsy Res. (2009) 84:91–6. doi: 10.1016/j.eplepsyres.2008.12.008 19201163

[78] Kerous B Barteček R Roman R Sojka P Bečev O Liarokapis F . Social environment simulation in VR elicits a distinct reaction in subjects with different levels of anxiety and somatoform dissociation. Int J Hum Comput Interact. (2020) 36:505–15. doi: 10.1080/10447318.2019.1661608

[79] Rosales R Dworetzky B Baslet G . Cognitive-emotion processing in psychogenic nonepileptic seizures. Epilepsy Behav. (2020) 102:106639. doi: 10.1016/j.yebeh.2019.106639 31731107

[80] Metin SZ Ozmen M Metin B Talasman S Yeni SN Ozkara C . Treatment with group psychotherapy for chronic psychogenic nonepileptic seizures. Epilepsy Behav. (2013) 28:91–4. doi: 10.1016/j.yebeh.2013.03.023 23680576

[81] Goldstein LH Mellers JD . Ictal symptoms of anxiety, avoidance behaviour, and dissociation in patients with dissociative seizures. J Neurol Neurosurg Psychiatry. (2006) 77:616–21. doi: 10.1136/jnnp.2005.066878 16614021 PMC2117432

[82] Sánchez de las Matas Dávila J . Anxiety, depression, stress in "depersonalization" and "derealization. Actas Luso Esp Neurol Psiquiatr Cienc Afines. (1994) 22:270–6. 7887208

[83] Hidalgo Rodrigo MI Díaz González RJ Lana Moliner A Pena García I Hidalgo Rodrigo MA . Depersonalization in panic disorders. Actas Luso Esp Neurol Psiquiatr Cienc Afines. (1997) 25:167–71. 9381960

[84] Michal M Wiltink J Zwerenz R Knebel A Schäfer A Nehring C . Depersonalization-derealization in the psychosomatic outpatient and consultation-liaison service. Z Psychosom Med Psychother. (2009) 55:215–28. 10.13109/zptm.2009.55.3.215 19886591

[85] Michal M Beutel ME . Depersonalisation/derealization - clinical picture, diagnostics and therapy. Z Psychosom Med Psychother. (2009) 55:113–40. doi: 10.13109/zptm.2009.55.2.113 19402018

[86] Heydrich L Marillier G Evans N Seeck M Blanke O . Depersonalization- and derealization-like phenomena of epileptic origin. Ann Clin Transl Neurol. (2019) 6:1739–47. doi: 10.1002/acn3.50870 31437864 PMC6764488

[87] Crellin A Millman LSM Hotopf M Pick S . Momentary predictors of dissociation in functional neurological disorder: An ecological momentary assessment-based pilot study. Front Psychiatry. (2026) 17:2026. doi: 10.3389/fpsyt.2026.1798482 42147025 PMC13171792

[88] Gerrans P . Pain asymbolia as depersonalization for pain experience. An interoceptive active inference account. Front Psychol. (2020) 11:523710. doi: 10.3389/fpsyg.2020.523710 33192765 PMC7658103

[89] Lebares CC Greenberg AL Ascher NL Delucchi KL Reilly LM van der Schaaf M . Exploration of individual and system-level well-being initiatives at an academic surgical residency program: A mixed-methods study. JAMA Netw Open. (2021) 4:e2032676. doi: 10.1001/jamanetworkopen.2020.32676 33404621 PMC7788470

[90] Ciaunica A McEllin L Kiverstein J Gallese V Hohwy J Woźniak M . Zoomed out: Digital media use and depersonalization experiences during the COVID-19 lockdown. Sci Rep. (2022) 12:3888. doi: 10.1038/s41598-022-07657-8 35273200 PMC8913838

[91] Lebois LAM Harnett NG van Rooij SJH Ely TD Jovanovic T Bruce SE . Persistent dissociation and its neural correlates in predicting outcomes after trauma exposure. Am J Psychiatry. (2022) 179:661–72. doi: 10.1176/appi.ajp.21090911 35730162 PMC9444876

[92] Hansen M Hyland P Armour C Andersen TE . Assessing the existence of dissociative PTSD in sub-acute patients of whiplash. J Trauma Dissociation. (2019) 20:16–31. doi: 10.1080/15299732.2018.1451805 29547063

[93] Carvalho T Pinto-Gouveia J Cunha M da Motta C . Experiential avoidance, uncompassionate self-responding, and peritraumatic depersonalization/derealization: A novel mediation model for war-related PTSD symptomatology. J Clin Psychol. (2022) 78:1074–92. doi: 10.1002/jclp.23303 34993963

[94] Schrader C Ross A . A review of PTSD and current treatment strategies. Mo Med. (2021) 118:546–51. 34924624 PMC8672952

[95] Brewin CR Atwoli L Bisson JI Galea S Koenen K Lewis-Fernández R . Post-traumatic stress disorder: evolving conceptualization and evidence, and future research directions. World Psychiatry. (2025) 24:52–80. doi: 10.1002/wps.21269 39810662 PMC11733483

[96] Hodny F Ociskova M Prasko J Vanek J Visnovsky J Sollar T . Early life experiences and adult attachment in obsessive-compulsive disorder: Part 2: Therapeutic effectiveness of combined cognitive behavioural therapy and pharmacotherapy in treatment-resistant inpatients. Neuroendocrinol Lett. (2022) 43:345–58. 36716392

[97] Hodny F Ociskova M Prasko J Houdkova M Vanek J Sollar T . Early life experiences and adult attachment in obsessive-compulsive disorder Part 1: Relationships between demographic, clinical, and psychological factors in pharmacoresistant OCD. Neuroendocrinol Lett. (2022) 43:333–44. 36716391

[98] Holubova M Prasko J Hodny F Vanek J Slepecky M Nesnidal V . Self-stigma, severity of psychopatology, dissociation, parental style and comorbid personality disorder in patient with neurotic spectrum disorders Part 2: Therapeutic efficacy of intensive psychotherapeutic inpatients program. Neuroendocrinol Lett. (2021) 42:185–99. 34279862

[99] Holubova M Prasko J Vaněk J Hodny F Nesnidal V Kasalova P . Self-stigma, severity of psychopatology, dissociation, parental style and comorbid personality disorder in patient with neurotic spectrum disorders Part 1: Relationships between self-stigma and clinical, psychosocial and demographic factors in neurotic spectrum disorder patients. Neuroendocrinol Lett. (2021) 42:99–112. 34217167

[100] Jay EL Nestler S Sierra M McClelland J Kekic M David AS . Ventrolateral prefrontal cortex repetitive transcranial magnetic stimulation in the treatment of depersonalization disorder: A consecutive case series. Psychiatry Res. (2016) 240:118–22. doi: 10.1016/j.psychres.2016.04.027 27104926 PMC4906152

[101] Beato-Fernandez L Muñoz-Martinez V Mata-Saenz B Gimeno-Clemente N Rojo-Moreno L Vaz-Leal FJ . Attitudes towards change mediate the effect of dissociation on psychopathological outcome in the treatment of eating disorders. Eur Eating Disord Rev. (2020) 28:724–38. doi: 10.1002/erv.2774 32770610

[102] Michal M Wiltink J Till Y Wild PS Münzel T Blankenberg S . Type-D personality and depersonalization are associated with suicidal ideation in the German general population aged 35-74: Results from the Gutenberg Heart Study. J Affect Disord. (2010) 125:227–33. doi: 10.1016/j.jad.2010.02.108 20206385

[103] Demirkol ME Uğur K Tamam L . The mediating effects of psychache and dissociation in the relationship between childhood trauma and suicide attempts. Anadolu Psikiyatri Dergisi. (2020) 21:453–60. doi: 10.5455/apd.82990 31622318

[104] Sierra M Gomez J Molina JJ Luque R Muñoz JF David AS . Depersonalization in psychiatric patients: A transcultural study. J Nerv Ment Dis. (2006) 194:356–61. doi: 10.1017/cbo9780511730023 16699385

[105] Gómez JM . Gendered sexual violence: Betrayal trauma, dissociation, and PTSD in diverse college students. J Aggression Maltreatment Trauma. (2021) 30:625–40. doi: 10.1080/10926771.2020.1783737 PMC9075698 35527804

[106] Čolić J Bassett TR Latysheva A Imboden C Bader K Hatzinger M . Depersonalization and derealization in embarrassing social interactions: An experience sampling study in social phobia, major depression and controls. J Anxiety Disord. (2020) 70:102189. doi: 10.1016/j.janxdis.2020.102189 32070861

[107] Mula M Pini S Cassano GB . The neurobiology and clinical significance of depersonalization in mood and anxiety disorders: A critical reappraisal. J Affect Disord. (2007) 99:91–9. doi: 10.1016/j.jad.2006.08.025 16997382

[108] Michal M Adler J Reiner I Wermke A Ackermann T Schlereth T . Association of neglect-like symptoms with anxiety, somatization, and depersonalization in complex regional pain syndrome. Pain Med. (2017) 18:764–72. doi: 10.1093/pm/pnw214 27605590

[109] Hoyer J Braeuer D Crawcour S Klumbies E Kirschbaum C . Depersonalization/derealization during acute social stress in social phobia. J Anxiety Disord. (2013) 27:178–87. doi: 10.1016/j.janxdis.2013.01.002 23434546

[110] Barreda-Ángeles M Hartmann T . Experiences of depersonalization/derealization among users of virtual reality applications: A cross-sectional survey. Cyberpsychol Behav Soc Netw. (2023) 26:22–7. doi: 10.1089/cyber.2022.0152 36595349

[111] Pons E Galante J Van Dam NT Lindner A . A cross-sectional survey on depersonalization/derealization and meditation-induced alterations of the self. Sci Rep. (2026) 16. doi: 10.31234/osf.io/6kyz9_v1 PMC13156312 42103836

[112] Fino E Jemmett-Skinner T Evans-Miller R Perkins J Malik M Robinson M . Dispositional traits, characteristic adaptations, and narrative identity reconstructions in individuals with depersonalization and derealization. J Pers. (2025) 93:796–810. doi: 10.1111/jopy.12976 39417577 PMC12053820

[113] Hunter EC Phillips ML Chalder T Sierra M David AS . Depersonalisation disorder: a cognitive-behavioural conceptualisation. Behav Res Ther. (2003) 41:1451–67. doi: 10.1016/s0005-7967(03)00066-4 14583413

[114] Michal M Wiltink J Tibubos AN Wild PS Münzel T Lackner K . Impact of depersonalization on the course of depression: longitudinal observations from the gutenberg health study. BMC Psychiatry. (2024) 24:196. doi: 10.1186/s12888-024-05658-7 38459472 PMC10924423

[115] Hunter EC Baker D Phillips ML Sierra M David AS . Cognitive-behaviour therapy for depersonalisation disorder: an open study. Behav Res Ther. (2005) 43:1121–30. doi: 10.1016/j.brat.2004.08.003 16005701

[116] Gillmeister H Šmate I Savva D Li H Parapadakis C Adler J . Confrontation with others' emotions changes bodily resonance differently in those with low and high levels of depersonalization. Philos Trans R Soc Lond B Biol Sci. (2024) 379:20230248. doi: 10.1098/rstb.2023.0248 39005042 PMC11444244

[117] Kusztor A Mulay N Yamada M Hohwy J Tsuchiya N . Lifting the veil: probing altered visual perception in derealization. Neurosci Conscious. (2025) 2025:niaf045. doi: 10.1093/nc/niaf045 41287817 PMC12640545

[118] Schlax J Wiltink J Beutel ME Münzel T Pfeiffer N Wild P . Symptoms of depersonalization/derealization are independent risk factors for the development or persistence of psychological distress in the general population: Results from the Gutenberg health study. J Affect Disord. (2020) 273:41–7. doi: 10.1016/j.jad.2020.04.018 32421621

[119] Michal M Zwerenz R Tschan R Edinger J Lichy M Knebel A . Screening for depersonalization-derealization with two items of the cambridge depersonalization scale. Psychother Psychosom Med Psychol. (2010) 60:175–9. 10.1055/s-0029-1224098 19544244

[120] Sierra M Lopera F Lambert MV Phillips ML David AS . Separating depersonalisation and derealisation: the relevance of the "lesion method. J Neurol Neurosurg Psychiatry. (2002) 72:530–2. 10.1136/jnnp.72.4.530 PMC1737835 11909918

[121] Fagioli F Telesforo L Dell'Erba A Consolazione M Migliorini V Patanè M . Depersonalization: An exploratory factor analysis of the Italian version of the Cambridge Depersonalization Scale. Compr Psychiatry. (2015) 60:161–7. doi: 10.1016/j.comppsych.2014.06.007 25863646

[122] Michal M Kaufhold J Engelbach U Lenz C Lischke M Overbeck G . The validity of the depersonalisation-derealisation scale of the narcissism--inventory. Psychother Psychosom Med Psychol. (2005) 55:512–6. doi: 10.1055/s-2005-866944 16342024

[123] Büetiger JR Hubl D Kupferschmid S Schultze-Lutter F Schimmelmann BG Federspiel A . Trapped in a glass bell jar: Neural correlates of depersonalization and derealization in subjects at clinical high-risk of psychosis and depersonalization-derealization disorder. Front Psychiatry. (2020) 11:535652. doi: 10.3389/fpsyt.2020.535652 33024435 PMC7516266

[124] Kirchhoff C Riedl D Kampling H Nolte T Kruse J Eke S . Beyond the mind: Depersonalization and derealization as risk factors for physical health in the German general population. J Psychosom Res. (2026) 208:112850. doi: 10.1016/j.jpsychores.2026.112850 42166955

[125] Michal M Sann U Niebecker M Lazanowski C Aurich S Kernhof K . Assessment of the depersonalization-derealization syndrome using the German version of the Dissociative Experiences Scale. Z Psychosom Med Psychother. (2004) 50:271–87. 10.13109/zptm.2004.50.3.271 15510349

[126] Moyano O Claudon P Colin V Svatos J Thiébaut E . Study of dissociative disorders and depersonalization in a sample of young adult French population. Encephale. (2001) 27:559–69. 11865563

[127] Millman LSM Hunter ECM Orgs G David AS Terhune DB . Symptom variability in depersonalization-derealization disorder: A latent profile analysis. J Clin Psychol. (2022) 78:637–55. doi: 10.1002/jclp.23241 34487354

[128] Millman LSM Hunter ECM David AS Orgs G Terhune DB . Assessing responsiveness to direct verbal suggestions in depersonalization-derealization disorder. Psychiatry Res. (2022) 315:114730. doi: 10.1016/j.psychres.2022.114730 35870293

[129] Elyoseph Z Geisinger D Zaltzman R Gordon CR Mintz M . How vestibular dysfunction transforms into symptoms of depersonalization and derealization? J Neurol Sci. (2023) 444:120530. doi: 10.1016/j.jns.2022.120530 36586207

[130] Millman LSM Hunter ECM Terhune DB Orgs G . Online structured dance/movement therapy reduces bodily detachment in depersonalization-derealization disorder. Complement Ther Clin Pract. (2023) 51:101749. doi: 10.1016/j.ctcp.2023.101749 37018935

[131] Zheng S Zhang FX Shum HPH Zhang H Song N Song M . Unraveling the brain dynamics of depersonalization-derealization disorder: a dynamic functional network connectivity analysis. BMC Psychiatry. (2024) 24:685. doi: 10.1186/s12888-024-06096-1 39402459 PMC11475637

[132] Pini S Nardi B Carpita B Lorenzi G Mula M Milrod B . Relationships between depersonalization-derealization symptoms and separation anxiety in adult patients with mood and anxiety disorders. Front Psychiatry. (2025) 16:1565217. doi: 10.3389/fpsyt.2025.1565217 40405883 PMC12095167

[133] Toupet M Van Nechel C Hautefort C Heuschen S Duquesne U Cassoulet A . Influence of visual and vestibular hypersensitivity on derealization and depersonalization in chronic dizziness. Front Neurol. (2019) 10:69. doi: 10.3389/fneur.2019.00069 30814972 PMC6381029

[134] Tibubos AN Grammes J Beutel ME Michal M Schmutzer G Brähler E . Emotion regulation strategies moderate the relationship of fatigue with depersonalization and derealization symptoms. J Affect Disord. (2018) 227:571–9. doi: 10.1016/j.jad.2017.11.079 29172049

[135] Hunter ECM Ring L Gafoor R Morant N Lewis G Perkins J . Cognitive behavior therapy for depersonalization-derealization disorder (CBT-f-DDD): a feasibility randomized trial. Pilot Feasibility Stud. (2025) 12:9. doi: 10.1186/s40814-025-01742-1 41373023 PMC12801544

[136] Hunter ECM Wong CLM Gafoor R Lewis G David AS . Cognitive behaviour therapy (CBT) for depersonalization derealization disorder (DDD): a self-controlled cross-over study of waiting list vs. active treatment. Cognit Behav Ther. (2023) 52:672–85. doi: 10.1080/16506073.2023.2255744 37711065

[137] Dewe H Watson DG Kessler K Braithwaite JJ . The depersonalized brain: New evidence supporting a distinction between depersonalization and derealization from discrete patterns of autonomic suppression observed in a non-clinical sample. Conscious Cognit. (2018) 63:29–46. doi: 10.1016/j.concog.2018.06.008 29929064

[138] Lemche E Surguladze SA Brammer MJ Phillips ML Sierra M David AS . Dissociable brain correlates for depression, anxiety, dissociation, and somatization in depersonalization-derealization disorder. CNS Spectr. (2016) 21:35–42. doi: 10.1017/s1092852913000588 24059962

[139] Sutar R Chaturvedi SK . Symptom profile and diagnostic utility of depersonalization-derealization disorder: A retrospective critical review from India. Indian J Psychiatry. (2020) 62:91–4. doi: 10.1007/978-3-319-73332-6_32 32001937 PMC6964445

[140] Song M Zheng S Song N Zhu H Jia Y Dai Z . Clinical characteristics of 217 Chinese cases with depersonalization/derealization disorder. BMC Psychiatry. (2024) 24:597. doi: 10.1186/s12888-024-06028-z 39232691 PMC11373400

[141] Zheng S Song N Wang S Ning Y Zhu H Song M . Potential targets for noninvasive brain stimulation on depersonalization-derealization disorder. Brain Sci. (2022) 12. doi: 10.3390/brainsci12081112 36009174 PMC9406113

[142] Liu S Jia Y Zheng S Feng S Zhu H Wang R . An experimental study of subliminal self-face processing in depersonalization-derealization disorder. Brain Sci. (2022) 12. doi: 10.3390/brainsci12121598 36552058 PMC9775423

[143] Ning Y Song N Zhu H Zheng S Jia Y Yin D . White matter abnormalities in first-episode patients with depersonalization/derealization disorder: A tract-based spatial statistics study. J Affect Disord. (2022) 309:19–26. doi: 10.1016/j.jad.2022.04.127 35469908

[144] Jia Y Song N Ning Y Zhu H Dong L Feng S . Altered self-referential-related brain regions in depersonalization-derealization disorder. Brain Behav. (2025) 15:e70314. doi: 10.1002/brb3.70314 39935045 PMC11813808

[145] Zheng S Song M Song N Zhu H Li X Yin D . Dysfunctional large-scale brain networks in drug-naïve depersonalization-derealization disorder patients. BMC Psychiatry. (2025) 25:59. doi: 10.1186/s12888-025-06497-w 39833729 PMC11749103

[146] Simeon D Stein DJ . Depersonalization/derealization and its relationship to mood and anxiety disorders in the National Comorbidity Survey-Replication (NCS-R). Soc Psychiatry Psychiatr Epidemiol. (2025). doi: 10.1007/s00127-025-02915-2 40317341

[147] Fleiss JL Gurland BJ Goldberg K . Independence of depersonalization-derealization. J Consult Clin Psychol. (1975) 43:110–1. doi: 10.1037/h0076319 1114229

[148] Cox BJ Swinson RP . Instrument to assess depersonalization-derealization in panic disorder. Depress Anxiety. (2002) 15:172–5. doi: 10.1002/da.10051 12112722

[149] Michal M Wiltink J Subic-Wrana C Zwerenz R Tuin I Lichy M . Prevalence, correlates, and predictors of depersonalization experiences in the German general population. J Nerv Ment Dis. (2009) 197:499–506. doi: 10.1097/nmd.0b013e3181aacd94 19597357

[150] Daniels JK Gaebler M Lamke JP Walter H . Grey matter alterations in patients with depersonalization disorder: a voxel-based morphometry study. J Psychiatry Neurosci. (2015) 40:19–27. doi: 10.1503/jpn.130284 25285875 PMC4275327

[151] Schlumpf YR Nijenhuis ERS Klein C Jäncke L Bachmann S . Resting-state functional connectivity in patients with a complex PTSD or complex dissociative disorder before and after inpatient trauma treatment. Brain Behav. (2021) 11:e02200. doi: 10.1002/brb3.2200 34105902 PMC8323038

[152] Paul ER Farmer M Kämpe R Cremers HR Hamilton JP . Functional connectivity between extrastriate body area and default mode network predicts depersonalization symptoms in major depression: Findings from an a priori specified multinetwork comparison. Biol Psychiatry Cognit Neurosci Neuroimaging. (2019) 4:627–35. doi: 10.1016/j.bpsc.2019.03.007 31103548

[153] Baker D Earle M Medford N Sierra M Towell A David A . Illness perceptions in depersonalization disorder: Testing an illness attribution model. Clin Psychol Psychother. (2007) 14:105–16. doi: 10.1002/cpp.518 41531421

[154] Simeon D Gross S Guralnik O Stein DJ Schmeidler J Hollander E . Feeling unreal: 30 cases of DSM-III-R depersonalization disorder. Am J Psychiatry. (1997) 154:1107–13. doi: 10.3928/0048-5713-19930701-09 9247397

[155] Pan X Palermo CA Kaplan CS Harnett NG Winternitz SR Kaufman ML . Anxiety sensitivity predicts depression severity in individuals with dissociative identity disorder. J Psychiatr Res. (2022) 155:263–8. doi: 10.1016/j.jpsychires.2022.09.003 36126396 PMC9588735

[156] Lebois LAM Palermo CA Scheuer LS Lebois EP Winternitz SR Germine L . Higher integration scores are associated with facial emotion perception differences in dissociative identity disorder. J Psychiatr Res. (2020) 129:197–204. doi: 10.1016/j.jpsychires.2020.02.007 32070885 PMC7057292

[157] Simeon D Guralnik O Schmeidler J Knutelska M . Fluoxetine therapy in depersonalisation disorder: Randomised controlled trial. Br J Psychiatry. (2004) 185:31–6. doi: 10.1192/bjp.185.1.31 15231553

[158] Hollander E Liebowitz MR DeCaria C Fairbanks J Fallon B Klein DF . Treatment of depersonalization with serotonin reuptake blockers. J Clin Psychopharmacol. (1990) 10:200–3. doi: 10.1097/00004714-199006000-00008 2115893

[159] Schweden TLK Konrad AC Wekenborg MK Hoyer J . Evaluation of a brief cognitive behavioral group intervention to reduce depersonalization in students with high levels of trait test anxiety: A randomized controlled trial. Anxiety Stress Coping. (2020) 33:266–80. doi: 10.1080/10615806.2020.1736936 32160798

[160] Lickel J Nelson E Lickel AH Deacon B . Interoceptive exposure exercises for evoking depersonalization and derealization: A pilot study. J Cogn Psychother. (2008) 22:321–30. doi: 10.1891/0889-8391.22.4.321 41472467

[161] Capron DW Norr AM Albanese BJ Schmidt NB . Fear reactivity to cognitive dyscontrol via novel head-mounted display perceptual illusion exercises. J Affect Disord. (2017) 217:138–43. doi: 10.1016/j.jad.2017.03.068 28410476 PMC12338040

[162] Molero-Zafra M Fernandez-Garcia O Mitjans-Lafont MT Perez-Marin M Hernandez-Jimenez MJ . Psychological intervention in women victims of childhood sexual abuse: A randomized controlled clinical trial comparing EMDR psychotherapy and trauma-focused cognitive behavioral therapy. Front Psychiatry. (2024) 15:1360388. doi: 10.3389/fpsyt.2024.1360388 38868491 PMC11167727

[163] Wigard I Meyerbroker K Ehring T Topper M Arntz A Emmelkamp P . Skills training followed by either EMDR or narrative therapy for posttraumatic stress disorder in adult survivors of childhood abuse: A randomized controlled trial. Eur J Psychotraumatol. (2024) 15:2332104. doi: 10.1080/20008066.2024.2332104 38629403 PMC11025408

[164] Burback L Yap S Purdon SE Abba-Aji A O’Shea K Brémault-Phillips S . Randomized controlled trial investigating web-based, therapist delivered eye movement desensitization and reprocessing for adults with suicidal ideation. Front Psychiatry. (2024) 15. doi: 10.3389/fpsyt.2024.1361086 38435978 PMC10904458

[165] Mazzoni GP Miglietta E Ciull T Rotundo L Pozza A Gonzalez A . Group eye movement desensitization reprocessing (EMDR) psychotherapy and recurrent interpersonal traumatic episodes: A pilot follow-up study. Clin Neuropsychiatry. (2022) 19:379–89. doi: 10.36131/cnfioritieditore20220605 PMC9807115 36627946

[166] Keefe JR Louka C Moreno A Spellun J Zonana J Milrod BL . Open trial of trauma-focused psychodynamic psychotherapy for posttraumatic stress disorder among LGBTQ individuals. Am J Psychother. (2023) 76:115–23. doi: 10.1176/appi.psychotherapy.20220037 37203147

[167] Schabinger N Gillmeister H Berti S Michal M Beutel ME Adler J . Detached and distracted: ERP correlates of altered attentional function in depersonalisation. Biol Psychol. (2018) 134:64–71. doi: 10.1016/j.biopsycho.2018.02.014 29486234

[168] Michal M Kaufhold J Grabhorn R Krakow K Overbeck G Heidenreich T . Depersonalization and social anxiety. J Nerv Ment Dis. (2005) 193:629–32. doi: 10.1016/j.eurpsy.2007.01.954 16131947

[169] Goldstein LH Drew C Mellers J Mitchell-O'Malley S Oakley DA . Dissociation, hypnotizability, coping styles and health locus of control: Characteristics of pseudoseizure patients. Seizure. (2000) 9:314–22. doi: 10.1053/seiz.2000.0421 10933985

[170] Simeon D Knutelska M Nelson D Guralnik O . Feeling unreal: a depersonalization disorder update of 117 cases. J Clin Psychiatry. (2003) 64:990–7. doi: 10.4088/jcp.v64n0903 14628973

[171] Beutel ME Brähler E Glaesmer H Kuss DJ Wölfling K Müller KW . Regular and problematic leisure-time Internet use in the community: results from a German population-based survey. Cyberpsychol Behav Soc Netw. (2011) 14:291–6. doi: 10.1089/cyber.2010.0199 21067277

[172] Michal M Glaesmer H Zwerenz R Knebel A Wiltink J Brähler E . Base rates for depersonalization according to the 2-item version of the Cambridge Depersonalization Scale (CDS-2) and its associations with depression/anxiety in the general population. J Affect Disord. (2011) 128:106–11. doi: 10.1016/j.jad.2010.06.033 20674036

[173] Fung HW Chan C Ross CA Wang EKS . Clinical features of a Chinese sample with self-reported symptoms of pathological dissociation. J Trauma Dissociation. (2021) 22:378–93. doi: 10.1080/15299732.2020.1869651 33427126

[174] Sar V Akyüz G Doğan O . Prevalence of dissociative disorders among women in the general population. Psychiatry Res. (2007) 149:169–76. doi: 10.1016/j.psychres.2006.01.005 17157389

[175] Brand BL Armstrong JG Loewenstein RJ . Psychological assessment of patients with dissociative identity disorder. Psychiatr Clinics North America. (2006) 29:145–68. doi: 10.1016/j.psc.2005.10.014 16530591

[176] Schwartz RA Chambless DL Barber JP Milrod B . Testing clinical intuitions about barriers to improvement in cognitive-behavioral therapy for panic disorder. Behav Ther. (2021) 52:956–69. doi: 10.1016/j.beth.2020.12.004 34134834 PMC8217733

[177] Michelson L June K Vives A Testa S Marchione N . The role of trauma and dissociation in cognitive-behavioral psychotherapy outcome and maintenance for panic disorder with agoraphobia. Behav Res Ther. (1998) 36:1011–50. doi: 10.1016/s0005-7967(98)00073-4 9737056

[178] Weiner E McKay D . A preliminary evaluation of repeated exposure for depersonalization and derealization. Behav Modif. (2013) 37:226–42. doi: 10.1177/0145445512461651 23118274

[179] Michal M Reuchlein B Adler J Reiner I Beutel ME Vögele C . Striking discrepancy of anomalous body experiences with normal interoceptive accuracy in depersonalization-derealization disorder. PloS One. (2014) 9:e89823. doi: 10.1371/journal.pone.0089823 24587061 PMC3937420

[180] Purcell JB Brand B Browne HA Chefetz RA Shanahan M Bair ZA . Treatment of dissociative identity disorder: leveraging neurobiology to optimize success. Expert Rev Neurother. (2024) 24:273–89. doi: 10.1080/14737175.2024.2316153 38357897 PMC10950423

[181] Ellason JW Ross CA . Millon Clinical Multiaxial Inventory-II follow-up of patients with dissociative identity disorder. psychol Rep. (1996) 78:707–18. doi: 10.2466/pr0.1996.78.3.707 8711025

[182] Ellason JW Ross CA . Two-year follow-up of inpatients with dissociative identity disorder. Am J Psychiatry. (1997) 154:832–9. doi: 10.1176/ajp.154.6.832 9167512

[183] Ullah S Khalily MT Ahmad I Hallahan B . The association of dissociative symptoms with exposure to trauma. Irish J psychol Med. (2018). doi: 10.1017/ipm.2017.84 30115198

[184] Schweden TLK Pittig A Bräuer D Klumbies E Kirschbaum C Hoyer J . Reduction of depersonalization during social stress through cognitive therapy for social anxiety disorder: A randomized controlled trial. J Anxiety Disord. (2016) 43:99–105. doi: 10.1016/j.janxdis.2016.09.005 27648752

[185] McNamara P Moore KH Papelis Y Diallo S Wildman WJ . Virtual reality-enabled treatment of nightmares. Dreaming. (2018) 28:205–24. doi: 10.1037/drm0000088

[186] Keating L Muller RT . LGBTQ+ based discrimination is associated with PTSD symptoms, dissociation, emotion dysregulation, and attachment insecurity among LGBTQ+ adults who have experienced trauma. J Trauma Dissociation. (2020) 21:124–41. doi: 10.1080/15299732.2019.1675222 31581904

[187] Segal DL . Diagnostic and statistical manual of mental disorders (DSM-IV-TR). In: The Corsini Encyclopedia of Psychology. Hoboken, NJ: John Wiley & Sons (2010). p. 1–3.

[188] Brewerton TD Perlman MM Gavidia I Suro G . The treatment of dissociative identity disorder in an eating disorder residential treatment setting. Int J Eating Disord. (2023) 56:815–28. doi: 10.1002/eat.24106 38041242

[189] Mohajerin B Lynn SJ Bakhtiyari M Dolatshah B . Evaluating the unified protocol in the treatment of dissociative identity disorder. Cogn Behav Pract. (2020) 27:447–58. doi: 10.1016/j.cbpra.2019.07.012 38826717

[190] Khattak T Farooq S Jan B . Behavior therapy in dissociative convulsions disorder. J Coll Physicians Surg Pak. (2006) 16:359–63. 16756783

[191] Bowman ES Markand ON . Psychodynamics and psychiatric diagnoses of pseudoseizure subjects. Am J Psychiatry. (1996) 153:57–63. 8540592 10.1176/ajp.153.1.57

[192] Kanner AM Parra J Frey M Stebbins G Pierre-Louis S Iriarte J . Psychiatric and neurologic predictors of psychogenic pseudoseizure outcome. Neurology. (1999) 53:933–8. doi: 10.1212/wnl.53.5.933 10496249

[193] Bravo TP Hoffman-Snyder CR Wellik KE Martin KA Hoerth MT Demaerschalk BM . The effect of selective serotonin reuptake inhibitors on the frequency of psychogenic nonepileptic seizures: a critically appraised topic. Neurologist. (2013) 19:30–3. doi: 10.1097/nrl.0b013e31827c6bfd 23269105

[194] Ozenli Y Ozisik HI Tugal O Yoldascan E . Health-related quality of life in patients with conversion disorder with seizures. Int J Psychiatry Clin Pract. (2008) 12:105–13. doi: 10.1080/13651500701679379 24916620

[195] LaFrance WC Jr. Baird GL Barry JJ Blum AS Frank Webb A Keitner GI . Multicenter pilot treatment trial for psychogenic nonepileptic seizures: a randomized clinical trial. JAMA Psychiatry. (2014) 71:997–1005. doi: 10.1001/jamapsychiatry.2014.817 24989152

[196] Alsaadi TM Marquez AV . Psychogenic nonepileptic seizures. Am Fam Physician. (2005) 72:849–56. doi: 10.1016/j.yebeh.2003.10.019 16156345

[197] O'Brien FM Fortune GM Dicker P O'Hanlon E Cassidy E Delanty N . Psychiatric and neuropsychological profiles of people with psychogenic nonepileptic seizures. Epilepsy Behav. (2015) 43:39–45. doi: 10.1016/j.yebeh.2014.11.012 25553390

[198] Rodríguez-Urrutia A Toledo M Eiroa-Orosa FJ Salas-Puig X Santamarina E Fidel-Kinori SG . Psychosocial factors and antiepileptic drug use related to delayed diagnosis of refractory psychogenic nonepileptic seizures. Cognit Behav Neurol. (2014) 27:199–205. doi: 10.1097/wnn.0000000000000045 25539039

[199] Grenevald L Gagny M Maillard L Chrusciel J Sanche S Schwan R . Post-traumatic factors are involved in the evolution of the number of seizures in patients with PNES. Epilepsy Behav. (2021) 115:107544. doi: 10.1016/j.yebeh.2020.107544 33423016

[200] Scévola L Wolfzun C Sarudiansky M Pico MMA Ponieman M Stivala EG . Psychiatric disorders, depression and quality of life in patients with psychogenic non-epileptic seizures and drug resistant epilepsy living in Argentina. Seizure. (2021) 92:174–81. doi: 10.1016/j.seizure.2021.09.004 34536854

[201] Schmidtke K . Functional memory disorders. A study of 25 patients. Nervenarzt. (1995) 66:338–46. 7609814

[202] Tezcaner Z Gökmen MF Yıldırım S Dursun G . Clinical features of psychogenic voice disorder and the efficiency of voice therapy and psychological evaluation. J Voice. (2019) 33:250–4. doi: 10.1016/j.jvoice.2017.09.022 29122418

[203] Bhatia MS Chandra R Vaid L . Psychogenic cough: A profile of 32 cases. Int J Psychiatry Med. (2002) 32:353–60. doi: 10.2190/9ctg-8fr7-fbqj-181v 12779185

[204] Mehta S Vora N Edgell RC Allam H Alawi A Koehne J . Stroke mimics under the drip-and-ship paradigm. J Stroke Cerebrovasc Dis. (2014) 23:844–9. doi: 10.1016/j.jstrokecerebrovasdis.2013.07.012 23954600

[205] Lenio S Baker S Watson M Libbon R Sillau S Strom L . Assessing the hidden burden of psychiatric disease in patients with nonepileptic seizures. Epilepsy Behav. (2021) 125:108382. doi: 10.1016/j.yebeh.2021.108382 34794013

[206] Faiman I Hodsoll J Jasani I Young AH Shotbolt P . Sociodemographic and clinical risk factors for suicidal ideation and suicide attempt in functional/dissociative seizures and epilepsy: A large cohort study. BMJ Ment Health. (2024) 27. doi: 10.1101/2023.11.27.23298811 38642918 PMC11033658

[207] Tilahun BBS Thompson NR Sankary LR Laryea F Trunick CM Jehi LE . Outcomes in the treatment of psychogenic nonepileptic seizures (PNES) with CBTip: Response in seizure frequency, depression, anxiety, and quality of life. Epilepsy Behav. (2021) 123:108277. doi: 10.1016/j.yebeh.2021.108277 34492542

[208] Myers L Matzner B Lancman M Perrine K Lancman M . Prevalence of alexithymia in patients with psychogenic non-epileptic seizures and epileptic seizures and predictors in psychogenic non-epileptic seizures. Epilepsy Behav. (2013) 26:153–7. doi: 10.1016/j.yebeh.2012.11.054 23314302

[209] Trobliger R Myers L Simpson T Krámská L . A comparison of patients with epileptic seizures (ES) versus those with psychogenic non-epileptic seizures (PNES) on measures of alexithymia, mood, and anxiety. Seizure. (2024) 120:33–40. doi: 10.1016/j.seizure.2024.06.010 38897162

[210] Cuoco S Nisticò V Cappiello A Scannapieco S Gambini O Barone P . Attachment styles, identification of feelings and psychiatric symptoms in functional neurological disorders. J Psychosom Res. (2021) 147:110539. doi: 10.1016/j.jpsychores.2021.110539 34091378

[211] Baillès E Pintor L Torres X Fernández-Egea E de Pablo J Arroyo S . Psychiatric disease in patients with psychogenic non-epileptic seizures referred from an epilepsy unit in a general hospital. Actas Esp Psiquiatr. (2004) 32:76–81. 15042467

[212] Gargiulo ÁJ Sarudiansky M Videla A Lombardi N Korman GP Oddo S . Perceived stress, resilience, and stress coping in patients with drug resistant epilepsy and functional dissociative seizures. Seizure. (2022) 101:141–8. doi: 10.1016/j.seizure.2022.08.002 36027685

[213] Ekanayake V Kranick S LaFaver K Naz A Frank Webb A LaFrance WC Jr. . Personality traits in psychogenic nonepileptic seizures (PNES) and psychogenic movement disorder (PMD): Neuroticism and perfectionism. J Psychosom Res. (2017) 97:23–9. doi: 10.1016/j.jpsychores.2017.03.018 28606495 PMC5572831

[214] Calderbank A Gray C Morgan-Boon A Reuber M . Changes in posttraumatic stress disorder symptoms with integrative psychotherapy for functional neurological symptom disorder. J Neuropsychiatry Clin Neurosci. (2023) 35:398–403. doi: 10.1176/appi.neuropsych.21070184 37089075

[215] Paredes-Echeverri S Guthrie AJ Perez DL . Toward a possible trauma subtype of functional neurological disorder: Impact on symptom severity and physical health. Front Psychiatry. (2022) 13:1040911. doi: 10.3389/fpsyt.2022.1040911 36458126 PMC9706184

[216] Van der Feltz-Cornelis CM Allen SF Van Eck van der Sluijs JF . Childhood sexual abuse predicts treatment outcome in conversion disorder/functional neurological disorder. An observational longitudinal study. Brain Behav. (2020) 10:e01558. doi: 10.1002/brb3.1558 32031757 PMC7066336

[217] LaFrance WC Jr. Deluca M Machan JT Fava JL . Traumatic brain injury and psychogenic nonepileptic seizures yield worse outcomes. Epilepsia. (2013) 54:718–25. doi: 10.1111/epi.12053 23281644

[218] TeBockhorst SF O'Halloran MS Nyline BN . Tonic immobility among survivors of sexual assault. Psychol Trauma. (2015) 7:171–8. doi: 10.1037/a0037953 25793694

[219] Testa SM Lesser RP Krauss GL Brandt J . Personality assessment inventory among patients with psychogenic seizures and those with epilepsy. Epilepsia. (2011) 52:e84-8. doi: 10.1111/j.1528-1167.2011.03141.x 21740416

[220] Tomić A Petrović I Pešić D Vončina MM Svetel M Mišković ND . Is there a specific psychiatric background or personality profile in functional dystonia? J Psychosom Res. (2017) 97:58–62. 28606500 10.1016/j.jpsychores.2017.04.004

[221] Villagrán A Lund C Duncan R Lossius MI . The effect of attachment style on long-term outcomes in psychogenic nonepileptic seizures: Results from a prospective study. Epilepsy Behav. (2022) 135:108890. doi: 10.1016/j.yebeh.2022.108890 36037581

[222] Jalilianhasanpour R Ospina JP Williams B Mello J MacLean J Ranford J . Secure attachment and depression predict 6-month outcome in motor functional neurological disorders: A prospective pilot study. Psychosomatics. (2019) 60:365–75. doi: 10.1016/j.psym.2018.08.004 30342702

[223] Sarudiansky M Pablo Korman G Lanzillotti AI Areco Pico MM Tenreyro C Paolasini GV . Report on a psychoeducational intervention for psychogenic non-epileptic seizures in Argentina. Seizure. (2020) 80:270–7. doi: 10.1016/j.seizure.2020.04.008 32475751

[224] Chalder T Landau S Stone J Carson A Reuber M Medford N . How does cognitive behavior therapy for dissociative seizures work? A mediation analysis of the CODES trial. Psychol Med. (2024) 54:1725–34. doi: 10.1017/s0033291723003665 38197148

[225] Rusch MD Morris GL Allen L Lathrop L . Psychological treatment of nonepileptic events. Epilepsy Behav. (2001) 2:277–83. doi: 10.1006/ebeh.2001.0180 12609370

[226] Goldstein LH Robinson EJ Pilecka I Perdue I Mosweu I Read J . Cognitive-behavioural therapy compared with standardised medical care for adults with dissociative non-epileptic seizures: The CODES RCT. Health Technol Assess. (2021) 25:1–144. doi: 10.3310/hta25430 34196269 PMC8273679

[227] LaFrance WC Jr. Miller IW Ryan CE Blum AS Solomon DA Kelley JE . Cognitive behavioral therapy for psychogenic nonepileptic seizures. Epilepsy Behav. (2009) 14:591–6. doi: 10.1016/j.yebeh.2009.02.016 19233313

[228] Guy L Caceres GA Jackson T Gorman S Wilson J Hsieh Y . Routine outcomes and evaluation of an 8-week outpatient multidisciplinary rehabilitative therapy program for functional neurological disorder. J Neurol. (2024) 271:1873–84. doi: 10.1007/s00415-023-12111-4 38091087 PMC10973040

[229] LaFrance WC Jr. Ho WLN Bhatla A Baird GL Altalib HH Godleski L . Treatment of psychogenic nonepileptic seizures (PNES) using video telehealth. Epilepsia. (2020) 61:2572–82. doi: 10.1111/epi.16689 33015831

[230] Kuyk J Siffels MC Bakvis P Swinkels WA . Psychological treatment of patients with psychogenic non-epileptic seizures: An outcome study. Seizure. (2008) 17:595–603. doi: 10.1016/j.seizure.2008.02.006 18395473

[231] Demartini B Batla A Petrochilos P Fisher L Edwards MJ Joyce E . Multidisciplinary treatment for functional neurological symptoms: A prospective study. J Neurol. (2014) 261:2370–7. doi: 10.1007/s00415-014-7495-4 25239392 PMC4242999

[232] Esteban-Serna C Loewenberger A Pick S Cope SR . Psychological therapy for functional neurological disorder: Examining impact on dissociation, psychological distress and general functioning. J Trauma Dissociation. (2024) 25:516–32. doi: 10.1080/15299732.2024.2356591 38780533

[233] Ataoglu A Ozcetin A Icmeli C Ozbulut O . Paradoxical therapy in conversion reaction. J Korean Med Sci. (2003) 18:581–4. doi: 10.3346/jkms.2003.18.4.581 12923337 PMC3055095

[234] Kompoliti K Wilson B Stebbins G Bernard B Hinson V . Immediate vs. delayed treatment of psychogenic movement disorders with short term psychodynamic psychotherapy: Randomized clinical trial. Parkinsonism Relat Disord. (2014) 20:60–3. doi: 10.1016/j.parkreldis.2013.09.018 24120952

[235] Petrochilos P Elmalem MS Patel D Louissaint H Hayward K Ranu J . Outcomes of a 5-week individualised MDT outpatient (day-patient) treatment programme for functional neurological symptom disorder (FNSD). J Neurol. (2020) 267:2655–66. doi: 10.1007/s00415-020-09874-5 32410018 PMC7419475

[236] Rizvi SJ Sproule BA Gallaugher L McIntyre RS Kennedy SH . Correlates of benzodiazepine use in major depressive disorder: The effect of anhedonia. J Affect Disord. (2015) 187:101–5. doi: 10.1016/j.jad.2015.07.040 26331683

[237] Magaudda A Gugliotta SC Tallarico R Buccheri T Alfa R Laganà A . Identification of three distinct groups of patients with both epilepsy and psychogenic nonepileptic seizures. Epilepsy Behav. (2011) 22:318–23. doi: 10.1016/j.yebeh.2011.07.005 21840769

[238] Dreissen YE Lambert F Dijk JM Koelman JH Tijssen MA . Botulinum neurotoxin (BoNT) treatment in functional movement disorders: Long-term follow-up. J Neurol Neurosurg Psychiatry. (2020) 91:1120–1. doi: 10.1136/jnnp-2020-323684 32661081 PMC7509513

[239] Goldstein LH Robinson EJ Mellers JDC Stone J Carson A Reuber M . Cognitive behavioural therapy for adults with dissociative seizures (CODES): A pragmatic, multicentre, randomised controlled trial. Lancet Psychiatry. (2020) 7:491–505. doi: 10.1016/s2215-0366(20)30128-0 32445688 PMC7242906

[240] Berry AJ Yuksel M Proctor BJ Foong J . Cognitive behavior therapy for comorbid dissociative seizures in patients with epilepsy. Epilepsy Behav. (2020) 106:106993. doi: 10.1016/j.yebeh.2020.106993 32169599

[241] Kishk N Raafat O Abdou H Nawito A Shamloul RM Belal M . Psychogenic nonepileptic seizures in patients with epilepsy: A comparative study with patients with pure epilepsy. J Nerv Ment Dis. (2021) 209:196–202. doi: 10.1097/nmd.0000000000001281 33315796

[242] Jobin K Wang M du Plessis S Silverberg ND Debert CT . The importance of screening for functional neurological disorders in patients with persistent post-concussion symptoms. NeuroRehabilitation. (2023) 53:199–208. doi: 10.3233/nre-237002 37638460

[243] Salinsky M Evrard C Storzbach D Pugh MJ . Psychiatric comorbidity in veterans with psychogenic seizures. Epilepsy Behav. (2012) 25:345–9. doi: 10.1016/j.yebeh.2012.07.013 23103308

[244] Mack J Quinn JF Lobb BM O'Connor S . Functional movement disorders in U.S. veterans: Psychiatric comorbidity and health care utilization. Mov Disord. (2019) 34:755–6. doi: 10.1002/mds.27650 30812060

[245] Silva W Giagante B Saizar R D'Alessio L Oddo S Consalvo D . Clinical features and prognosis of nonepileptic seizures in a developing country. Epilepsia. (2001) 42:398–401. doi: 10.1046/j.1528-1157.2001.45299.x 11442159

[246] Naidoo L Bhigjee AI . The spectrum of functional neurological disorders: A retrospective analysis at a tertiary hospital in South Africa. S Afr J Psychiatr. (2021) 27:1607. doi: 10.4102/sajpsychiatry.v27i0.1607 33936802 PMC8063761

[247] Nisticò V Goeta D Gambini O Demartini B . The psychological impact of COVID-19 among a sample of Italian patients with functional neurological disorders: A preliminary study. Parkinsonism Relat Disord. (2020) 78:79–81. doi: 10.1016/j.parkreldis.2020.07.019 32745981 PMC7374122

[248] Taylor J Jonsson G Paruk L Philippides A . A South African review of routinely-collected health data of psychogenic nonepileptic seizure patients referred to psychiatrists in Johannesburg. Epilepsy Behav. (2021) 114:107578. doi: 10.1016/j.yebeh.2020.107578 33268018

[249] Osman AH Alsharief SM Siddig HE . Functional neurological disorder: Characteristics and outcome in a limited-resources country (Sudan). Epilepsy Behav. (2020) 111:107151. doi: 10.1016/j.yebeh.2020.107151 32698104

[250] Scheidt CE Köster B Deuschl G . Diagnosis, symptoms and follow-up of psychogenic tremor. Nervenarzt. (1996) 67:198–204. 8901277

[251] Régny P Cathébras P . Conversion disorder in an internal medicine department: A series of 37 cases. Encephale. (2016) 42:150–5. doi: 10.1016/j.encep.2014.11.004 26827119

[252] Allanson J Bass C Wade DT . Characteristics of patients with persistent severe disability and medically unexplained neurological symptoms: A pilot study. J Neurol Neurosurg Psychiatry. (2002) 73:307–9. doi: 10.1136/jnnp.73.3.307 12185165 PMC1738051

[253] Mazza M Martini A Valenti MA Scoppetta M Vaccario ML Rodriguez R . Psychopathology in epilepsy and pseudoepilepsy: Preliminary results in our experience. Clin Ter. (2006) 157:219–23. 16900847

[254] Myers L Trobliger R Bortnik K Lancman M . Are there gender differences in those diagnosed with psychogenic nonepileptic seizures? Epilepsy Behav. (2018) 78:161–5. doi: 10.1016/j.yebeh.2017.10.019 29183659

[255] Dreissen YEM Koelman J Tijssen MAJ . The auditory startle response in relation to outcome in functional movement disorders. Parkinsonism Relat Disord. (2021) 89:113–7. doi: 10.1016/j.parkreldis.2021.07.012 34274620

[256] Saunders C Bawa H Aslanyan D Coleman F Jinadu H Sigala N . Treatment outcomes in the inpatient management of severe functional neurological disorder: A retrospective cohort study. BMJ Neurol Open. (2024) 6:e000675. doi: 10.1136/bmjno-2024-000675 38979396 PMC11227748

[257] Keeley R Smith M Miller J . Somatoform symptoms and treatment nonadherence in depressed family medicine outpatients. Arch Fam Med. (2000) 9:46–54. doi: 10.1001/archfami.9.1.46 10664642

[258] Krámská L Myers L Hrešková L Krámský D Vojtěch Z . Diagnostic utility of the Minnesota multiphasic personality inventory-2 in patients diagnosed with psychogenic non-epileptic seizures in the Czech Republic. Epilepsy Behav. (2021) 115:107698. doi: 10.3233/978-1-60750-096-4-23 33385953

[259] Sojka P Lošák J Lamoš M Bareš M Kašpárek T Brázdil M . Processing of emotions in functional movement disorder: An exploratory fMRI study. Front Neurol. (2019) 10. doi: 10.3389/fneur.2019.00861 31474926 PMC6703143

[260] Teodoro T Koreki A Chen J Coebergh J Poole N Ferreira JJ . Functional cognitive disorder affects reaction time, subjective mental effort and global metacognition. Brain. (2023) 146:1615–23. doi: 10.1093/brain/awac363 36200349

[261] Schwilk N Klöppel S Schmidtke K Metternich B . Functional cognitive disorder in subjective cognitive decline-a 10-year follow-up. Int J Geriatr Psychiatry. (2021) 36:677–83. doi: 10.1002/gps.5466 33166421

[262] Andersson C Marklund K Walles H Hagman G Miley-Akerstedt A . Lifestyle factors and subjective cognitive impairment in patients seeking help at a memory disorder clinic: The role of negative life events. Dement Geriatr Cognit Disord. (2019) 48:196–206. doi: 10.1159/000505573 31982880

[263] Calma AD Heffernan J Farrell N Gelauff J O'Connell N Perez DL . The impact of depression, anxiety and personality disorders on the outcome of patients with functional limb weakness – Individual patient data meta-analysis. J Psychosomatic Res. (2023) 175:111513. doi: 10.1016/j.jpsychores.2023.111513 37832273

[264] Boyd JE Protopopescu A O’Connor C Neufeld RWJ Jetly R Hood HK . Dissociative symptoms mediate the relation between PTSD symptoms and functional impairment in a sample of military members, veterans, and first responders with PTSD. Eur J Psychotraumatol. (2018) 9. doi: 10.1080/20008198.2018.1463794 29805778 PMC5965037

[265] Baker CT . Documenting the effects of noninvasive prefrontal pIR HEG neurofeedback in the treatment of common mental health problems. NeuroRegulation. (2023) 10:207–18. doi: 10.15540/nr.10.3.207

[266] Craparo G La Rosa VL Marino G Vezzoli M Cinà GS Colombi M . Risk of post-traumatic stress symptoms in hospitalized and non-hospitalized COVID-19 recovered patients. A cross-sectional study. Psychiatry Res. (2022) 308:114353. doi: 10.1016/j.psychres.2021.114353 34968807 PMC8688161

[267] Hummel KV Schellong J Trautmann S Kummer S Hürrig S Klose M . The predictive role of hair cortisol concentrations for treatment outcome in PTSD inpatients. Psychoneuroendocrinology. (2021) 131:105326. doi: 10.1016/j.psyneuen.2021.105326 34182250

[268] Saçmacı H Cengiz GF Aktürk T . Impact of dissociative experiences in migraine and its close relationship with osmophobia. Neurological Res. (2020) 42:529–36. 10.1080/01616412.2020.1753417 32295514

[269] Grosse-Holz VM Nikendei C Andermann M Ringleb PA Friederich H-C Rizos T . Predictors of posttraumatic stress following transient ischemic attack: An observational cohort study. J Psychosomatic Res. (2020) 137:110205. doi: 10.1016/j.jpsychores.2020.110205 32768689

[270] Kolek A Prasko J Ociskova M Holubova M Vanek J Grambal A . Severity of panic disorder, adverse events in childhood, dissociation, self-stigma and comorbid personality disorders Part 2: Therapeutic effectiveness of a combined cognitive behavioural therapy and pharmacotherapy in treatment-resistant inpatients. Neuro Endocrinol Lett. (2019) 40:271–83. 32200586

[271] Pozza A Dèttore D . Was it real or did I imagine it? Perfectionistic beliefs are associated with dissociative absorption and imaginative involvement in obsessive-compulsive disorder. Psychol Res Behav Manage. (2019) 12:603–7. doi: 10.2147/prbm.s212983 31447594 PMC6682762

[272] Kolek A Prasko J Vanek J Kantor K Holubova M Slepecky M . Severity of panic disorder, adverse events in childhood, dissociation, self-stigma and comorbid personality disorders: Part 1: Relationships between clinical, psychosocial and demographic factors in pharmacoresistant panic disorder patients. Neuroendocrinol Lett. (2019) 40:233–46. 32112548

[273] Ociskova M Prasko J Vrbova K Kasalova P Holubova M Grambal A . Self-stigma and treatment effectiveness in patients with anxiety disorders- a mediation analysis. Neuropsychiatr Dis Treat. (2018) 14:383–92. doi: 10.2147/ndt.s152208 29416340 PMC5790087

[274] Soffer-Dudek N Somer E . Trapped in a daydream: Daily elevations in maladaptive daydreaming are associated with daily psychopathological symptoms. Front Psychiatry. (2018) 9. doi: 10.3389/fpsyt.2018.00194 29867613 PMC5962718

[275] Hyland P Shevlin M Fyvie C Karatzias T . Posttraumatic stress disorder and complex posttraumatic stress disorder in DSM-5 and ICD-11: Clinical and behavioral correlates. J Traumatic Stress. (2018) 31:174–80. doi: 10.1002/jts.22272 29577450

[276] Kong SS Kang DR Oh MJ Kim NH . Attachment insecurity as a mediator of the relationship between childhood trauma and adult dissociation. J Trauma Dissociation. (2018) 19:214–31. doi: 10.1080/15299732.2017.1329772 28509622

[277] Fung HW Hung SL Ling HW Lee VWP Lam SKK . A preliminary longitudinal analysis of symptom management, post-traumatic stress, and depressive symptoms in Chinese adults with dissociative symptoms. J Trauma Dissociation. (2024) 25:129–43. doi: 10.1080/15299732.2023.2231908 37394873

[278] Nester MS Nicholas AP Gavi S Bethany LB . Characteristics, methods, and functions of non-suicidal self-injury among highly dissociative individuals. J Trauma Dissociation. (2023). doi: 10.1080/15299732.2023.2181475 36803534

[279] Gewirtz-Meydan A Lassri D . Sex in the shadow of child sexual abuse: The development and psychometric evaluation of the post-traumatic sexuality (PT-SEX) scale. J Interpersonal Violence. (2023) 38:4714–41. doi: 10.1177/08862605221118969 36000712

[280] Jans T Schneck-Seif S Weigand T Schneider W Ellgring H Wewetzer C . Long-term outcome and prognosis of dissociative disorder with onset in childhood or adolescence. Child Adolesc Psychiatry Ment Health. (2008) 2:19. doi: 10.1186/1753-2000-2-19 18651951 PMC2517058

[281] Smith GC Clarke DM Handrinos D Dunsis A McKenzie DP . Consultation-liaison psychiatrists' management of somatoform disorders. Psychosomatics. (2000) 41:481–9. doi: 10.1176/appi.psy.41.6.481 11110111

[282] Golshani S Najafpour A Hashemian SS Goudarzi N Firoozabadi A Ghezelbash MS . Individuals with major depressive disorder report high scores of insecure-avoidant and insecure-anxious attachment styles, dissociative identity symptoms, and adult traumatic events. Healthcare (Basel). (2021) 9. doi: 10.3390/healthcare9091169 34574943 PMC8469763

[283] Selvi Y Kiliç S Aydin A Güzel Özdemir P . The effects of sleep deprivation on dissociation and profiles of mood, and its association with biochemical changes. Noro Psikiyatr Ars. (2015) 52:83–8. doi: 10.5152/npa.2015.7116 28360682 PMC5353008

[284] Oztürk E Sar V . Somatization as a predictor of suicidal ideation in dissociative disorders. Psychiatry Clin Neurosci. (2008) 62:662–8. doi: 10.1111/j.1440-1819.2008.01865.x 19068002

[285] Berkol TD Balcioglu YH Kirlioglu SS Erensoy H Vural M . Dissociative features of fibromyalgia syndrome. Neurosciences. (2017) 22:198–204. doi: 10.17712/nsj.2017.3.20160538 28678214 PMC5946364

[286] Möller A Söndergaard HP Helström L . Tonic immobility during sexual assault – a common reaction predicting post-traumatic stress disorder and severe depression. Acta Obstetricia Gynecologica Scandinavica. (2017) 96:932–8. doi: 10.1111/aogs.13174 28589545

[287] Akyüz F Gökalp PG Erdıman S Oflaz S Karşidağ Ç . Conversion disorder comorbidity and childhood trauma. Noropsikiyatri Arsivi. (2017) 54:15–20. doi: 10.5152/npa.2017.19184 28566953 PMC5439465

[288] Vyskocilova J Prasko J Sipek J . Cognitive behavioral therapy in pharmacoresistant obsessive–compulsive disorder. Neuropsychiatr Dis Treat. (2016) 12:625–39. doi: 10.2147/ndt.s101721 27042074 PMC4798215

[289] Saxe GN van der Kolk BA Berkowitz R Chinman G Hall K Lieberg G . Dissociative disorders in psychiatric inpatients. Am J Psychiatry. (1993) 150:1037–42. 10.1176/ajp.150.7.1037 8317573

[290] Kitamura T Nakamura M Miura I Fujinawa A . Symptoms of neuroses: Profile patterns and factor structure of clinic attenders with non-psychotic functional psychiatric disorders. Psychopathology. (1997) 30:191–9. doi: 10.1159/000285047 9239790

[291] Fink P Hansen MS Oxhøj ML . The prevalence of somatoform disorders among internal medical inpatients. J Psychosom Res. (2004) 56:413–8. doi: 10.1016/s0022-3999(03)00624-x 15094025

[292] Leonard D Brann S Tiller J . Dissociative disorders: Pathways to diagnosis, clinician attitudes and their impact. Aust N Z J Psychiatry. (2005) 39:940–6. doi: 10.1111/j.1440-1614.2005.01700.x 16168022

[293] Sar V Koyuncu A Ozturk E Yargic LI Kundakci T Yazici A . Dissociative disorders in the psychiatric emergency ward. Gen Hosp Psychiatry. (2007) 29:45–50. doi: 10.1016/j.genhosppsych.2006.10.009 17189745

[294] Gulsun M Doruk A Uzun O Turkbay T Ozsahin A . Effect of dissociative experiences on drug treatment of panic disorder. Clin Drug Investig. (2007) 27:583–90. doi: 10.2165/00044011-200727080-00007 17638399

[295] Majohr KL Leenen K Grabe HJ Jenewein J Nunez DG Rufer M . Alexithymia and its relationship to dissociation in patients with panic disorder. J Nerv Ment Dis. (2011) 199:773–9. doi: 10.1097/nmd.0b013e31822fcbfb 21964271

[296] Sar V Akyüz G Oztürk E Alioğlu F . Dissociative depression among women in the community. J Trauma Dissociation. (2013) 14:423–38. doi: 10.1080/15299732.2012.753654 23796173

[297] Ural C Belli H Tabo A Akbudak M . Open-longitudinal study of the effect of dissociative symptoms on the response of patients with panic disorder to venlafaxine. Compr Psychiatry. (2015) 57:112–6. doi: 10.1016/j.comppsych.2014.11.016 25492225

[298] Măirean C Ceobanu CM . The relationship between suppression and subsequent intrusions: the mediating role of peritraumatic dissociation and anxiety. Anxiety Stress Coping. (2017) 30:304–16. doi: 10.1080/10615806.2016.1263839 27873541

[299] Pettorruso M D’andrea G Martinotti G Cocciolillo F Miuli A Di Muzio I . Hopelessness, dissociative symptoms, and suicide risk in major depressive disorder: clinical and biological correlates. Brain Sci. (2020) 10:1–11. doi: 10.3390/brainsci10080519 32764310 PMC7465542

[300] Prasko J Ociskova M Grambal A Sigmundova Z Kasalova P Marackova M . Personality features, dissociation, self-stigma, hope, and the complex treatment of depressive disorder. Neuropsychiatr Dis Treat. (2016) 12:2539–52. doi: 10.2147/ndt.s117037 27785031 PMC5063494

[301] Ociskova M Prasko J Latalova K Kamaradova D Grambal A . Psychological factors and treatment effectiveness in resistant anxiety disorders in highly comorbid inpatients. Neuropsychiatr Dis Treat. (2016) 12:1539–51. doi: 10.2147/ndt.s104301 27445474 PMC4928674

[302] da Silva TLG Donat JC Lorenzonni PL de Souza LK Gauer G Kristensen CH . Event centrality in trauma and PTSD: relations between event relevance and posttraumatic symptoms. Psicologia: Reflexão e Crítica. (2016) 29:34. doi: 10.1186/s41155-016-0015-y 38164791

[303] Ociskova M Prasko J Kamaradova D Grambal A Kasalova P Sigmundova Z . Coping strategies, hope, and treatment efficacy in pharmacoresistant inpatients with neurotic spectrum disorders. Neuropsychiatr Dis Treat. (2015) 11:1191–201. doi: 10.2147/ndt.s80325 26028972 PMC4440432

[304] Caslini M Rivolta L Zappa LE Carrà G Clerici M . Psychotherapeutic treatment of eating disorders improve dissociative experiences and impulse regulation but not alexithymia. A case series report. Rivista di Psichiatria. (2015) 50:143–7. doi: 10.1708/1910.20798 26156820

[305] Dorahy MJ Corry M Shannon M Webb K McDermott B Ryan M . Complex trauma and intimate relationships: the impact of shame, guilt and dissociation. J Affect Disord. (2013) 147:72–9. doi: 10.1016/j.jad.2012.10.010 23141670

[306] Guidi J Fava GA Picardi A Porcelli P Bellomo A Grandi S . Subtyping depression in the medically ill by cluster analysis. J Affect Disord. (2011) 132:383–8. doi: 10.1016/j.jad.2011.03.004 21458076

[307] Rufer M Held D Cremer J Fricke S Moritz S Peter H . Dissociation as a predictor of cognitive behavior therapy outcome in patients with obsessive-compulsive disorder. Psychother Psychosom. (2006) 75:40–6. doi: 10.1159/000089225 16361873

[308] Hallings-Pott C Waller G Watson D Scragg P . State dissociation in bulimic eating disorders: an experimental study. Int J Eating Disord. (2005) 38:37–41. doi: 10.1002/eat.20146 15971242

[309] Bowman S Coons PM . The use of electroconvulsive therapy in patients with dissociative disorders. J Nervous Ment Dis. (1992) 180(8):524–8. doi: 10.1097/00005053-199208000-00008 1500935

[310] Mavissakalian MR . Phenomenology of panic attacks: responsiveness of individual symptoms to imipramine. J Clin Psychopharmacol. (1996) 16:233–7. doi: 10.1097/00004714-199606000-00007 8784655

[311] Kaplan ML Asnis GM Lipschitz DS Chorney P . Suicidal behavior and abuse in psychiatric outpatients. Compr Psychiatry. (1995) 36:229–35. doi: 10.1016/0010-440x(95)90087-c 7648848

[312] Marshall RD Schneier FR Lin SH Simpson HB Vermes D Liebowitz M . Childhood trauma and dissociative symptoms in panic disorder. Am J Psychiatry. (2000) 157:451–3. doi: 10.1176/appi.ajp.157.3.451 10698824

[313] Wise TN Mann LS Sheridan MJ . Relationship between alexithymia, dissociation and personality in psychiatric outpatients. Psychother Psychosom. (2000) 69:123–7. doi: 10.1159/000012379 10773775

[314] Prueter C Schultz-Venrath U Rimpau W . Dissociative and associated psychopathological symptoms in patients with epilepsy, pseudoseizures, and both seizure forms. Epilepsia. (2002) 43:188–92. doi: 10.1046/j.1528-1157.2002.45900.x 11903467

[315] Kellner M Yassouridis A Hua Y Wendrich M Naber D Wiedemann K . Trait dissociation affects the behavioral response to cholecystokinin tetrapeptide in healthy man. Psychiatry Res. (2002) 111:93–6. doi: 10.1016/s0165-1781(02)00144-0 12140124

[316] Agargun MY Kara H Ozer OA Selvi Y Kiran U Ozer B . Clinical importance of nightmare disorder in patients with dissociative disorders. Psychiatry Clin Neurosci. (2003) 57:575–9. doi: 10.1046/j.1440-1819.2003.01169.x 14629705

[317] Lochner C Seedat S Hemmings SM Moolman-Smook JC Kidd M Stein DJ . Investigating the possible effects of trauma experiences and 5-HTT on the dissociative experiences of patients with OCD using path analysis and multiple regression. Neuropsychobiology. (2007) 56:6–13. doi: 10.1159/000109971 17943026

[318] Giesbrecht T Merckelbach H ter Burg L Cima M Simeon D . Acute dissociation predicts rapid habituation of skin conductance responses to aversive auditory probes. J Trauma Stress. (2008) 21:247–50. doi: 10.1002/jts.20323 18404635

[319] Ebrinc S Semiz UB Basoglu C Cetin M Agargun MY Algul A . Self-mutilating behavior in patients with dissociative disorders: the role of innate hypnotic capacity. Isr J Psychiatry Relat Sci. (2008) 45:39–48. 18587168

[320] Lynch SM Forman E Mendelsohn M Herman J . Attending to dissociation: assessing change in dissociation and predicting treatment outcome. J Trauma Dissociation. (2008) 9:301–19. doi: 10.1080/15299730802139063 19042780

[321] Dedić G Djurdjević S Golubović B . Psychological assessment of persons following suicide attempt by self-poisoning. Vojnosanit Pregl. (2010) 67:151–8. doi: 10.2298/vsp1002151d 20337098

[322] La Mela C Maglietta M Castellini G Amoroso L Lucarelli S . Dissociation in eating disorders: relationship between dissociative experiences and binge-eating episodes. Compr Psychiatry. (2010) 51:393–400. doi: 10.1016/j.comppsych.2009.09.008 20579513

[323] Alexopoulos J Steinberg C Liebergesell-Kilian NE Hoeffkes B Doering S Junghöfer M . Biased emotional attention in patients with dental phobia. Eur J Neurosci. (2019) 49:290–302. doi: 10.1111/ejn.14295 30506590 PMC6590303

[324] Tanner J Zeffiro T Wyss D Perron N Rufer M Mueller-Pfeiffer C . Psychiatric symptom profiles predict functional impairment. Front Psychiatry. (2019) 10:37. doi: 10.3389/fpsyt.2019.00037 30853916 PMC6396718

[325] Didonna F Lanfredi M Xodo E Ferrari C Rossi R Pedrini L . Mindfulness-based cognitive therapy for obsessive-compulsive disorder: a pilot study. J Psychiatr Pract. (2019) 25:156–70. doi: 10.1097/pra.0000000000000377 30849066

[326] Ociskova M Prasko J Vanek J Holubova M Hodny F Latalova K . Self-stigma and treatment effectiveness in patients with SSRI non-responsive obsessive-compulsive disorder. Psychol Res Behav Manag. (2021) 14:85–97. doi: 10.2147/prbm.s287419 33574718 PMC7873032

[327] Mash HBH Ursano RJ Kessler RC Naifeh JA Fullerton CS Aliaga PA . Predictors of suicide attempt within 30 days of first medically documented major depression diagnosis in U.S. army soldiers with no prior suicidal ideation. BMC Psychiatry. (2023) 23:392. doi: 10.1186/s12888-023-04872-z 37268952 PMC10239190

[328] Seitz KI Sicorello M Schmitz M Valencia N Herpertz SC Bertsch K . Childhood maltreatment and amygdala response to interpersonal threat in a transdiagnostic adult sample: the role of trait dissociation. Biol Psychiatry Cognit Neurosci Neuroimaging. (2024) 9:626–34. doi: 10.1016/j.bpsc.2024.01.003 38280631

[329] Hinrichsen H Wright F Waller G Meyer C . Social anxiety and coping strategies in the eating disorders. Eating Behav. (2003) 4:117–26. doi: 10.1016/s1471-0153(03)00016-3 15000975

[330] Place PJ Ling S Patihis L . Full statistical mediation of the relationship between trauma and depressive symptoms. Int J Psychol. (2018) 53:142–9. doi: 10.1002/ijop.12279 27169590

[331] Sayın A Candansayar S Welkin L . Group psychotherapy in women with a history of sexual abuse: what did they find helpful? J Clin Nurs. (2013) 22:3249–58. doi: 10.1111/jocn.12168 24118587

[332] Gül AI Şimşek GG Inanir S Karaaslan O . Is there a dissociative subtype of generalized social phobia? J Psychiatry. (2014) 17(6):1000168. doi: 10.4172/Psychiatry.1000168

[333] Semiz UB Inanc L Bezgin CH . Are trauma and dissociation related to treatment resistance in patients with obsessive-compulsive disorder? Soc Psychiatry Psychiatr Epidemiol. (2014) 49:1287–96. doi: 10.1007/s00127-013-0787-7 24213522

[334] Claes L Vandereycken W Vertommen H . Eating-disordered patients with and without self-injurious behaviours: a comparison of psychopathological features. Eur Eating Disord Rev. (2003) 11:379–86. doi: 10.1002/erv.510 41531421

[335] Ray S Ray R Singh N Paul I . Dissociative experiences and health anxiety in panic disorder. Indian J Psychiatry. (2021) 63(1):70–3. doi: 10.4103/psychiatry.Indianjpsychiatry_896_20 34083823 PMC8106434

[336] Pozza A Torniai S Dèttore D . Inferential confusion moderates the effects of dissociative experiences on OCD symptoms severity in a clinical sample with obsessive-compulsive disorder. Clin Neuropsychiatry. (2016) 13:108–14.

[337] Prasko J Kamaradova D Cakirpaloglu S Talova B Diveky T Grambal A . Predicting the therapeutic response to intensive psychotherapeutic programs in patients with neurotic spectrum disorders. Activitas Nervosa Superior Rediviva. (2015) 57:30–9. doi: 10.1016/s0924-9338(12)74310-9 38826717

[338] Bersani G Moscariello MA Bersani FS Colletti C Anastasia A Prinzivalli E . Dissociative symptoms in female patients with mood and anxiety disorders: a psychopathological and temperamental investigation. Eur Rev Med Pharmacol Sci. (2014) 18(21):3217–22. 25487931

[339] Kamaradova D Prasko J Brunovsky M Grambal A Diveky T Latalova K . What are demographic and EEG differences between responding and non-responding panic disorder patients. Neuro Endocrinol Lett. (2013) 34:162–71. 23645314

[340] Hetzel-Riggin MD Wilber EL . To dissociate or suppress? Predicting automatic vs. conscious cognitive avoidance. J Trauma Dissociation. (2010) 11:444–57. doi: 10.1080/15299732.2010.495376 20938868

[341] Spitzer C Barnow S Freyberger HJ Grabe HJ . Dissociation predicts symptom-related treatment outcome in short-term inpatient psychotherapy. Aust N Z J Psychiatry. (2007) 41:682–7. doi: 10.1080/00048670701449146 17620165

[342] Kamaradova D Prasko J Sandoval A Latalova K . Therapeutic response to complex cognitive-behavioral and pharmacological treatment in patients with social phobia. Activitas Nervosa Superior Rediviva. (2014) 56:91–9.

[343] Dvir Y Ford JD Hill M Frazier JA . Childhood maltreatment, emotional dysregulation, and psychiatric comorbidities. Harv Rev Psychiatry. (2014) 22:149–61. doi: 10.1097/hrp.0000000000000014 24704784 PMC4091823

[344] Patron VG Rustomji Y Yip C Jenkins LM . Psychiatric comorbidities in functional neurologic symptom disorder. Pract Neurol (Fort Wash Pa). (2022) 21:71–5. 36644502 PMC9836030

[345] Wilkhoo HS Islam AW Reji F Sanghvi L Potdar R Solanki S . Depersonalization-derealization disorder: etiological mechanism, diagnosis and management. Discoveries (Craiova). (2024) 12:e190. doi: 10.15190/d.2024.09 40093848 PMC11910194

[346] Campbell MC Smakowski A Rojas-Aguiluz M Goldstein LH Cardeña E Nicholson TR . Dissociation and its biological and clinical associations in functional neurological disorder: systematic review and meta-analysis. BJPsych Open. (2022) 9:e2. doi: 10.1192/bjo.2022.597 36451595 PMC9798224

[347] Černis E Antonović M Kamvar R Perkins J . Depersonalisation-derealisation as a transdiagnostic treatment target: a scoping review of the evidence in anxiety, depression, and psychosis. Front Psychol. (2025) 16:1531633. doi: 10.3389/fpsyg.2025.1531633 40309203 PMC12042760

