# Supplementary material for: Co-occurrence of dissociation and anxiety and depressive disorders in adults: a scoping review
Source: Front Psychiatry. 2026 Aug 13;17:1866005. doi: 10.3389/fpsyt.2026.1866005 (PMC13518564; doi:10.3389/fpsyt.2026.1866005)
Supplement: Supplementary file 1 [file Supplementaryfile1.docx]

*Supplementary Material 1. Search strategy for the scoping review.*

Database: PubMed (MEDLINE)

(

"Dissociation"[Mesh]

OR dissociat*[tiab]

OR depersonalization[tiab]

OR derealization[tiab]

OR "depersonalization disorder"[tiab]

OR "dissociative symptoms"[tiab]

OR "dissociative experiences"[tiab]

OR "dissociative identity disorder"[tiab]

)

AND

(

"Depressive Disorder"[Mesh]

OR "Depression"[Mesh]

OR depress*[tiab]

OR "major depressive disorder"[tiab]

OR MDD[tiab]

)

AND

(

"Anxiety Disorders"[Mesh]

OR anxiety[tiab]

OR "panic disorder"[tiab]

OR "generalized anxiety disorder"[tiab]

OR GAD[tiab]

OR "social anxiety"[tiab]

)

AND

(

adult*[tiab]

OR adults[Mesh]

)

**Database: Scopus**

(

TITLE-ABS-KEY(dissociat*

OR depersonalization

OR derealization

OR "dissociative symptoms"

OR "dissociative experiences"

OR "dissociative identity disorder")

)

AND

(

TITLE-ABS-KEY(depress*

OR "major depressive disorder"

OR MDD)

)

AND

(

TITLE-ABS-KEY(anxiety

OR "anxiety disorder*"

OR "panic disorder"

OR "generalized anxiety disorder"

OR GAD

OR "social anxiety")

)

AND

(

TITLE-ABS-KEY(adult*)

)

*Supplementary Material 2. Data extraction framework and predefined variables*

| **Main category** | **Subcategories** |
| --- | --- |
| **Publication details** | Authors  Title  Year of publication  Journal  Country of origin |
| **Study characteristics** | Study type  Study design  Primary objective  Secondary objective |
| **Participant characteristics** | Number of participants  Age  Gender  Nationality  Diagnostic information |
| **Intervention characteristics (where applicable)** | Type of intervention (e.g., pharmacotherapy, psychotherapy, or both)  Duration of treatment or exposure  Follow-up period  Use of comparator  Participant adherence  Assessment tools |
| **Outcomes** | Rate of symptom improvement |
| **Findings** | Main findings  Secondary findings |
| **Study limitations** | Reported limitations |
| **Authors’ recommendations** | Recommendations for clinical practice and/or future research |
